# Spatiotemporal and ontogenetic variation, microbial selection, and predicted *Bd*-inhibitory function in the skin-associated microbiome of a Rocky Mountain amphibian

**DOI:** 10.3389/fmicb.2022.1020329

**Published:** 2022-12-13

**Authors:** Kenen B. Goodwin, Jaren D. Hutchinson, Zachariah Gompert

**Affiliations:** ^1^Department of Watershed Sciences, Utah State University, Logan, UT, United States; ^2^Department of Wildland Resources, Utah State University, Logan, UT, United States; ^3^Department of Biology, Utah State University, Logan, UT, United States

**Keywords:** temporal variation, spatial variation, bacteria, fungi, relative abundance, Bayesian analysis

## Abstract

Host-associated microbiomes play important roles in host health and pathogen defense. In amphibians, the skin-associated microbiota can contribute to innate immunity with potential implications for disease management. Few studies have examined season-long temporal variation in the amphibian skin-associated microbiome, and the interactions between bacteria and fungi on amphibian skin remain poorly understood. We characterize season-long temporal variation in the skin-associated microbiome of the western tiger salamander (*Ambystoma mavortium*) for both bacteria and fungi between sites and across salamander life stages. Two hundred seven skin-associated microbiome samples were collected from salamanders at two Rocky Mountain lakes throughout the summer and fall of 2018, and 127 additional microbiome samples were collected from lake water and lake substrate. We used 16S rRNA and ITS amplicon sequencing with Bayesian Dirichlet-multinomial regression to estimate the relative abundances of bacterial and fungal taxa, test for differential abundance, examine microbial selection, and derive alpha diversity. We predicted the ability of bacterial communities to inhibit the amphibian chytrid fungus *Batrachochytrium dendrobatidis* (*Bd*), a cutaneous fungal pathogen, using stochastic character mapping and a database of *Bd*-inhibitory bacterial isolates. For both bacteria and fungi, we observed variation in community composition through time, between sites, and with salamander age and life stage. We further found that temporal trends in community composition were specific to each combination of salamander age, life stage, and lake. We found salamander skin to be selective for microbes, with many taxa disproportionately represented relative to the environment. Salamander skin appeared to select for predicted *Bd*-inhibitory bacteria, and we found a negative relationship between the relative abundances of predicted *Bd*-inhibitory bacteria and *Bd*. We hope these findings will assist in the conservation of amphibian species threatened by chytridiomycosis and other emerging diseases.

## Introduction

Host-associated microbiomes can interact with their hosts in many ways. Specialized metabolites produced by microbes can influence various aspects of host biology (Sharon et al., 2014), and host production of antimicrobial peptides can in turn influence microbial community structure (McFall-Ngai et al., 2013). Microbial communities are increasingly recognized as providing beneficial and necessary services for their hosts (Dethlefsen et al., 2007; Grice and Segre, 2011), and maintaining and restoring healthy microbiomes can be important for host health (Tosh and McDonald, 2012). Host-associated microbiomes can inhibit pathogens or parasites through competition, the activation of host immune responses, and the production of inhibitory secondary metabolites (Lee and Mazmanian, 2010; Britton and Young, 2014; Grunseich et al., 2019). An imbalance in the host-associated microbiome can permit transient opportunistic pathogens and resident microbes with pathogenic potential to harm the host (Lee and Mazmanian, 2010).

Much attention has been given to the amphibian skin-associated microbiome’s role in innate immunity for its potential in disease management (Walke and Belden, 2016). Chytridiomycosis is a devastating amphibian skin disease caused by the fungal pathogen *Batrachochytrium dendrobatidis* (hereafter *Bd*; Longcore et al., 1999; Skerratt et al., 2007). Because numerous amphibian skin-associated bacteria have been found to inhibit the growth of *Bd*, probiotic bioaugmentation and habitat management have the potential to influence susceptibility to chytridiomycosis (Harris et al., 2009; Kueneman et al., 2016a; Grant et al., 2018). A sound understanding of host-associated microbiomes and their natural range of variation is necessary to select effective probiotics for safe and successful probiotic bioaugmentation strategies (Bletz et al., 2013).

While amphibian skin-associated microbiomes are species-specific, vary with life history stage, and are distinct from environmental microbiomes (i.e., soil, lake substrate, and lake water microbiomes), some variation in the microbiomes is attributable to location and abiotic water quality (McKenzie et al., 2011; Kueneman et al., 2013; Walke et al., 2014; Bletz et al., 2017a,b; Ellison et al., 2019). The composition of skin-associated microbial communities has been found to vary between larval and metamorphosed life stages in both frog and salamander species, with community diversity being higher in the adults of these species than their larvae (Kueneman et al., 2013, 2016b; Sabino-Pinto et al., 2017). Temperature has been found to influence operational taxonomic unit (OTU) richness and the production of antifungal metabolites in amphibian skin-associated microbiomes (Daskin et al., 2014; Muletz-Wolz et al., 2019).

Although many studies have worked to characterize species-specific and spatial variation in the amphibian skin-associated microbiome, fine-scale season-long temporal variation in natural systems remains a major gap in our knowledge of the amphibian skin-associated microbiome with few applicable studies (Sabino-Pinto et al., 2017; Bletz et al., 2017a). Since both *Bd* infection prevalence and amphibian skin-associated microbiomes show seasonal and year-to-year variation (Savage et al., 2011; Longo et al., 2015; Familiar López et al., 2017; Douglas et al., 2021; Basanta et al., 2022), season-long temporal variation in the amphibian skin-associated microbiome warrants investigation for its implications in disease management.

Using a database of amphibian skin-associated *Bd*-inhibitory bacterial isolates and their 16S rRNA gene sequences (Woodhams et al., 2015), Bletz et al. (2017a) found that despite significant changes in bacterial community structure on the skin of salamandrid newts, the relative abundances of bacteria with *Bd*-inhibitory potential did not change significantly during a 12-week sampling period nor across life history stages in two of the three species studied. Sabino-Pinto et al. (2017) found bacterial communities on the skin of two salamandrid newt species to change significantly between months, and also using the database of Woodhams et al. (2015), the study found the relative abundance of putative *Bd*-inhibitory bacteria to be higher on the skin of larvae compared to adults for one of the species. The database by Woodhams et al. (2015) contains nearly 2,000 bacterial isolates tested for *Bd*-inhibitory function *in vitro* assays, with about half of the isolates being from Central-South America. However, the application of this database to predict amphibian skin-associated microbiome *Bd*-inhibitory function is limited by our knowledge of how these bacterial isolates function on amphibian skin. Observing fungal responses to changes in bacterial abundances could assist in detecting bacterial-fungal relationships.

Despite the focus of many amphibian skin-associated microbiome studies on bacteria, few studies have examined how bacteria interact with non-*Bd* fungal taxa and other microeukaryotes on amphibian skin (Kueneman et al., 2016b, 2017; Belasen et al., 2021). For example, Kueneman et al. (2016b) found many correlations between bacterial and fungal taxa on the skin of the western toad (*Anaxyrus boreas*), and the authors proposed that larval stages of amphibians may depend on high relative abundances of antifungal bacteria to confer innate immunity before metamorphosis and the maturation of the host adaptive immune system (Rollins-Smith, 1998). Hence, the interactions between bacteria and fungi on amphibian skin may have substantial implications for host health and disease management.

Broadly, our study aims to investigate temporal variation in the amphibian skin-associated microbiome using the western tiger salamander (*Ambystoma mavortium*; hereafter salamander) as a model amphibian. In the Rocky Mountains of North America, the western tiger salamander serves as an apex predator in many fishless high alpine lakes. When the snow melts at these lakes, adult salamanders travel from upland to the lakes to breed, and some of these salamanders remain in the lakes throughout the early summer. During the summer months, eggs hatch and larval salamanders may follow several life history strategies, including metamorphosing during the same year as hatching, overwintering as larvae and metamorphosing the following year, and becoming sexually mature in the larval stage as neotenes (Sexton and Bizer, 1978). Due to their local abundance and the presence of at least one life stage throughout the warm months (June to September, hereafter warm season) at fishless high alpine lakes, the western tiger salamander is an ideal amphibian for consistently obtaining skin-associated microbiome samples throughout the warm season.

In this study, we first examine season-long temporal variation of both bacteria and fungi in the salamander skin-associated microbiome between sites and across life history stages, and we consider whether temporal trends are similar between sites and life stages. Based on these data, we identify differentially abundant microbes between salamander skin and the environment and compare the predictive ability of spatiotemporal and water quality covariates on microbial community composition. We then ask (i) whether variation in the salamander skin-associated microbiome influences predicted *Bd*-inhibitory function, and (ii) whether predicted *Bd*-inhibitory function is correlated with the relative abundance of *Bd*.

## Materials and methods

### Study sites

Salamanders were sampled from the largest of the Gibson Lakes (Franklin County, ID; 447,845 easting, 4,654,056 northing, NAD 83 UTM Zone 12; elevation: 2,579 m) and Ponds Lake (Summit County, UT; 503,020 easting, 4,503,670 northing, NAD 83 UTM Zone 12; elevation: 3,058 m). These lakes were chosen for sampling due to their differences in geology, substrate, and water conditions. We chose to sample lakes with different environmental conditions in order to investigate whether temporal trends in microbiome composition on salamander skin were similar between different lake environments. Both lakes are fishless, have no tributaries or outlets, and are located in different subranges of the Rocky Mountains. Gibson Lakes is a ~ 2.5-ha shallow lake in a limestone basin of the Bear River Mountains. Patches of submerged vegetation cover much of the lake bottom, and the lake substrate is primarily composed of soft sticky mud. Ponds Lake is a ~ 2.3-ha lake in a granitic basin of the Uinta Mountains. The lake substrate is a thick layer of loose vegetative material, and some parts of the shoreline have floating mats of vegetation. The water in Ponds Lake is stained red with dissolved organic carbon.

In 2018, access to Gibson Lakes was blocked due to snow at lower elevations until June 9th, when salamander eggs were observed attached to submerged vegetation. By the next week, when field sampling began, most of the previously observed eggs had hatched. Data from NRCS SNOTEL sites (see Supplementary material) suggest that snow melted at both lakes at about the same time in 2018, possibly within days of each other, and snow typically melts at these lakes about a week apart. Based on these data, it is likely that salamanders laid eggs in 2018 at about the same time at both lakes.

### Sampling design

**Figure 1:**
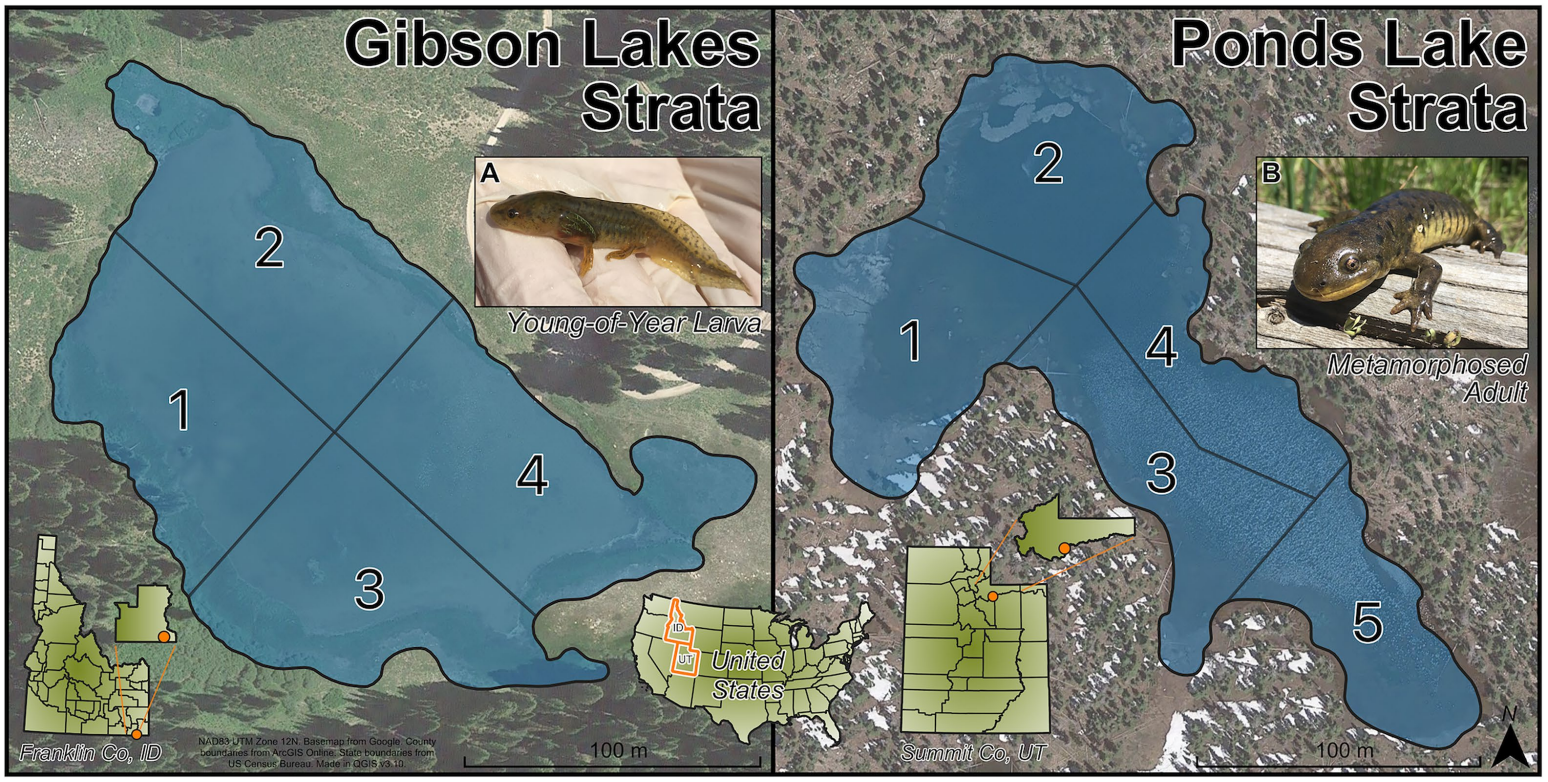
Strata for Gibson Lakes (left) and Ponds Lake (right). Images of **(A)** a young-of-year larval salamander and **(B)** a metamorphosed adult salamander.

To ensure that the sampled salamanders were distributed throughout the lakes, the lakes were sampled by strata. Gibson Lakes was assigned 4 strata and Ponds Lake was assigned 5 strata (Figure 1). The strata divide the lakes into regions based on natural landmarks for easy recognition in the field. Within a lake, all strata had roughly the same area, and their areas remained roughly the same as each other as water levels dropped throughout the warm season. Three age classes of salamanders could be distinguished based on length and weight measurements, age-0, age-1, and age-2+. These age classes were of distinctly different sizes, with the length and weight of each age class increasing throughout the warm season (Supplementary Figure S1). During each visit to a lake (hereafter sampling event), we collected up to 20 salamanders from each age class with a maximum of five and four salamanders per stratum at Gibson Lakes and Ponds Lake, respectively. Each lake was sampled every other week during the 2018 warm season. Sampling began shortly after snowmelt and continued until the lakes became too cold to safely catch salamanders. Sampling began at Gibson Lakes on June 16th and Ponds Lake on June 23rd. Gibson Lakes was too cold to sample on September 29th, marking the end of the field season.

Salamanders were considered larvae if they retained any of their larval gill structures, and salamanders were considered metamorphosed individuals once all traces of their gill structures were absorbed. For each age class, larval and metamorphosed individuals were encountered, which we refer to as life stages, and we refer to the six possible combinations of age class and life stage as stage classes. We expect most age-2+ individuals to be sexually mature adults, at which point gilled individuals are considered neotenes.

### Data collection

Upon arriving at a lake, environmental microbiome samples and water quality data were collected. During the first visit to each lake, a location was selected just offshore in each stratum to collect these samples and data. These locations were chosen to have relatively homogeneous depths across strata and to minimize the distance which the sampling location would need to move with receding water levels. Water quality data was collected prior to collecting environmental microbiome samples to minimize disturbance to the water. Water temperature, pH, electrical conductivity, and dissolved oxygen (ppm and percent) were measured just below the water surface using handheld meters (Hannah Instruments HI98129 and HI9146). For sampling the lake water microbiome, 500 ml of lake water was collected from the water surface in a laboratory Nalgene bottle. Following collection of a lake water microbiome sample, a lake substrate microbiome sample was collected from the top ~10 cm of pond substrate using a small PVC clam gun. The substrate column was deposited into a 15-ml conical tube, and excess water was decanted. The substrate was thoroughly stirred with a teasing needle, and ~ 1.5 ml of substrate was deposited into a sterile 2-ml microcentrifuge tube. The microcentrifuge tubes containing substrate samples were placed on ice in a cooler while in the field. New latex gloves were worn for each environmental microbiome sample, and the clam gun and teasing needle were rinsed with 95% ethanol between substrate samples. The clam gun and teasing needle were rinsed with 6% bleach solution followed by a thorough rinse with distilled water between sampling events. Nalgene bottles were rinsed thoroughly with distilled water and autoclaved for 20 min at 121°C between holding lake water samples. During each sampling event, the depth of a predefined rock was measured to determine relative lake elevation, the water level of the lake relative to its height at the beginning of the warm season.

After collecting environmental microbiomes and water quality data for all strata, salamanders were captured for each stratum. Salamanders were collected by hand and dip net, and salamanders were stored in 5-gallon buckets filled with lake water. For each stratum, different age classes were stored in separate 5-gallon buckets to reduce the risk of smaller salamanders being harmed from predation or aggression from larger individuals. While storing salamanders from the same age class and stratum together in 5-gallon buckets could have allowed for microbial contamination between individuals, we suspect that potential contamination between individuals was minimal for the following reasons. First, only a few individuals were stored together at a time (an average of 3.04 and maximum of five individuals). Second, the period of time which individuals were stored together was short (typically about 25 min). Finally, individuals tended to disperse themselves relatively evenly within the buckets, and contact between individuals and the ventral surfaces (the body region of interest) of others was rare.

Each salamander was handled with new latex gloves, and snout-vent length (SVL) and weight measurements were taken to verify age classes (Supplementary Figure S1). Sex was determined for age-2+ salamanders. The ventral surface of each salamander was rinsed with 50 ml of distilled water (Bletz et al., 2017a) to remove environmental material and transient microbes (Culp et al., 2007; Lauer et al., 2007), and the salamander’s ventral surface was swabbed with a sterile rayon-tipped swab (MW113 Medical Wire and Equipment, Corsham, United Kingdom). Swabbing was performed by stroking the swab across the ventral surface ten times (one time = an up and back stroke along the full length of the belly; Bletz et al., 2017a). Swabs used to sample salamander skin-associated microbiomes were stored in individual sterile 2-ml microcentrifuge tubes and placed on ice in a cooler while in the field. After processing salamanders for a stratum was complete, the salamanders were released back into the stratum, and salamander collection began at the next stratum. While it is possible that salamanders sampled in one stratum may have been sampled again in another stratum during the same sampling event, few salamanders were observed to have swum far from their point of release, which was away from stratum borders.

For each sampling event, the lake was surveyed for salamanders for a minimum of 5 person-hours divided evenly among the lake’s strata. Salamanders were processed after the stratum minimum sampling time was reached or the maximum number of individuals from all available age classes had been collected, and the search for salamanders then proceeded to the next stratum. After field sampling and while still at the lake, wet and dry negative control swabs were taken. Wet control swabs were sprayed with 50 ml of distilled water, and nothing was done to the dry control swabs. Wet and dry control swabs were placed in individual sterile 2-ml microcentrifuge tubes and stored on ice in a cooler while in the field.

Following field sampling and on the same day, lake water samples were prefiltered through a 5.0-μm prefilter membrane to remove debris followed by filtration with a 0.22-μm filter membrane to catch microbes (Millipore Sigma SVLP02500 and GSWP04700, respectively). Multiple 5.0-μm prefilter membranes were used for each water sample as necessary, whereas samples which experienced clogging on the 0.22-μm filter membrane (three samples) were discarded. Following filtration, 0.22-μm filter membranes were folded and stored in 2-ml microcentrifuge tubes. For autoclavable filtration equipment, the equipment was rinsed thoroughly with distilled water between water samples followed by autoclaving for 20 min at 121°C. Non-autoclavable filtration equipment was rinsed with 6% bleach solution followed by a thorough rinse with distilled water between water samples. Every four or five sampling events, five 500-ml distilled water samples were filtered as negative controls.

All samples were transferred to a −80°C freezer for storage, and the typical time from field collection to freezer storage was about five-and-a-half hours. Salamanders were collected, stored, handled, and released according to an approved Utah State University Institutional Animal Care and Use Committee protocol (#2798), a Utah Division of Wildlife Resources Certificate of Registration (#2COLL10232), and an Idaho Department of Fish and Game Wildlife Collection/Banding/Possession Permit (#180110).

### DNA extraction and library preparation

DNA was extracted with the DNeasy PowerSoil Pro Kit (Qiagen, Inc.) following the manufacturer’s protocol, and 12 empty extractions were performed as blank negative controls. Substrate samples were centrifuged for 30 s at 10,000 × *g*, excess liquid was removed with a pipette, and a scoopula was used to collect 250 mg of substrate from each sample for DNA extraction. Water sample filter membranes were finely diced using scissors and forceps into reagent reservoirs before being transferred to DNA extraction tubes. Swab samples were transferred to DNA extraction tubes using a different pair of forceps than that used for water samples. Pre-DNA extraction sample preparation work was performed under a fume hood, and the scoopula, scissors, and forceps were rinsed with 95% ethanol, flamed, and rinsed thoroughly with distilled water between samples. Reagent troughs were rinsed thoroughly with distilled water and autoclaved for 20 min at 121°C between water samples.

Following DNA extraction, two samples of ZymoBIOMICS Microbial Community DNA Standard (Zymo Research D6305) were added as mock community positive sequencing controls. 6 μl of a control oligo pool was added to 30 μl of full concentration DNA extract. The control oligo pool contained 0.01 pg/μl each of 16S and ITS well-specific cross contamination oligos (hereafter coligos; Hawkins et al., 2018) and 0.03 pg/μl each of 16S and ITS synthetic genes (hereafter synthgenes; Tourlousse et al., 2017). The addition of fixed amounts of 16S and ITS synthgenes to a constant volume of each sample’s DNA extract will be used later in estimating the amount of microbial DNA in each sample. Sample DNA concentrations were measured *via* absorption and normalized to 10 ng/μl with an automated liquid handler. Combinatorial dual indexing was performed on the samples with two-stage polymerase chain reaction (PCR). First stage PCR amplified the 16S rRNA and ITS genetic barcoding regions, added unique dual index combinations to each sample, and added a portion of the Illumina Nextera adapter. For each sample, two first-stage PCR replicates were performed and subsequently pooled. Second stage PCR completed Illumina adapter addition. The 16S rRNA V4 region was amplified using the primers 515F (forward; Parada et al., 2016) and 806R (reverse; Caporaso et al., 2011). The ITS1 region was amplified using the primers ITS1-F (forward; Gardes and Bruns, 1993) and ITS2 (reverse; White et al., 1990). A modified AxyPrep MagBead PCR Clean-up protocol was used to purify the amplified DNA after each PCR reaction. Library preparation occurred at the University of Wyoming Genome Technologies Laboratory (Laramie, WY). See Supplementary material for library preparation details.

### DNA sequencing and processing

Paired-end DNA sequencing of pooled amplicon product was performed on both Illumina MiSeq (v3 600-cycle kit, 2 × 300 base pair [bp] reads) and Illumina NextSeq (v2 300-cycle kit, 2 × 150 bp reads) platforms at the Utah State University Center for Integrated Biosystems (Logan, UT). Both sequencing platforms offer their own advantages for 16S and ITS amplicon sequencing, where Illumina MiSeq produces longer but fewer reads than Illumina NextSeq. The longer MiSeq sequences provide greater taxonomic resolution, and the greater number of NextSeq sequences reduces uncertainty in relative abundance estimates. We leverage the benefits of both sequencing platforms by using the longer-length MiSeq sequences as study-specific 16S and ITS reference libraries to enhance the taxonomic resolution of our shorter but more numerous NextSeq sequences. Illumina MiSeq produced 19 million paired-end reads, and Illumina NextSeq produced 187 million paired-end reads.

MiSeq reads were partitioned into 16S and ITS datasets based on their primer regions using a custom Perl script (version 5.18.1; see Data Availability for script), and index tags were removed. Since variable length index tags were used, MiSeq reads were trimmed to 290 bp using cutadapt (version 2.10; Martin, 2011) to ensure that non-overlapping sequences did not appear different simply due to read length. Using cutadapt, read pairs that contained Ns were removed, and forward primers and reverse complements of reverse primers were trimmed (with a maximum error rate of 0.15, a minimum trimmed length of 1 bp, and discarding untrimmed read pairs), with trimming the reverse primer’s reverse complement being required for 16S read pairs. Variable length index tags reduce amplicon sequencing error on Illumina platforms by increasing heterogeneity in the composition of bases called in each cycle (Fadrosh et al., 2014).

The DADA2 bioinformatics pipeline (version 3.10; Callahan et al., 2016) was used in the R statistical software program (version 4.0.2; R Core Team, 2020) for quality filtering, phiX removal, denoising, merging pairs, chimera removal, and taxonomic assignment of MiSeq reads (see Supplementary material for details). While 16S reads were of appropriate lengths for merging pairs, the variable length of the ITS region resulted in both overlapping and non-overlapping read pairs. DADA2 has the ability to work with both overlapping and non-overlapping read pairs, allowing for the retention of fungal taxa with long ITS genes. Overlapping ITS read pairs were merged, while non-overlapping ITS read pairs were retained in the pipeline as concatenated sequences with 10-N spacers, which DADA2’s implementation of the naïve Bayesian classifier is designed to work with. DADA2’s naïve Bayesian classifier (Wang et al., 2007) was used to classify unique sequences in the MiSeq 16S and ITS datasets using Silva (version 138; Quast et al., 2012) and UNITE (general dynamic FASTA release for fungi; version 8.2; Nilsson et al., 2019) reference libraries, respectively. To create study-specific 16S and ITS reference libraries, NextSeq-length forward and reverse reads were created from the classified MiSeq 16S and ITS sequences, and consensus taxonomies and MiSeq-length sequences (for predicting *Bd*-inhibitory function) were generated for duplicate reference read pairs (see Supplementary material for details). Integers were appended to reference taxa names to differentiate each amplicon sequence variant (ASV) associated with a taxon.

NextSeq reads were assigned to PCR replicate, barcode region, and sample (i.e., reads were demultiplexed) using Perl while allowing 1 bp mismatches in the index tags (index tags were designed to differ by at least 2 bp). Allowing 1 bp mismatches in the index tags allows reads which experience sequencing error in the index tag regions to be retained if the index tags can still be uniquely identified. We note that allowing sequencing errors in index tag regions is not uncommon during demultiplexing. For example, demultiplexing in cutadapt and QIIME 2 (qiime cutadapt demux-paired command; version 2022.8; Bolyen et al., 2019) allow for 10% mismatches in index tags by default. Following demultiplexing, phiX reads were discarded and index tags were removed using Perl. The following steps were performed sequentially on the NextSeq reads using cutadapt: reads were trimmed to 140 bp to make all reads the same length, read pairs with Ns were removed, forward primers and reverse complements of reverse primers were trimmed (with the same settings as the MiSeq data but without requiring trimming of the reverse primers’ reverse complements).

Using exact matching in R, 21.4 million of 54.3 million NextSeq 16S reads were identified to 15,792 reference sequences, and 60.4 million of 113.9 million NextSeq ITS reads were identified to 3,488 reference sequences. Of the identified NextSeq sequences, 17.0% of 16S sequences were coligos or the synthgene, and 79.5% of ITS sequences were coligos or the synthgene. All samples were checked for between-well cross contamination through use of the coligos. Three salamander samples and one blank control sample were removed from the 16S dataset due to high amounts of between-well contamination (having a ratio of any contaminant coligo to non-contaminant coligo greater than 0.1 after summing coligo read counts across PCR replicates). Two salamander samples were removed from the ITS dataset due to lack of detection of any non-synthgene and non-coligo sequences in both PCR replicates. Coligos were removed from the datasets for all subsequent analyses. In the mock community samples, we observed strong amplification bias in the ITS data (Supplementary Figure S2), and one fungal taxon was split into three substantial ASVs. In an effort to mitigate the potential impact of fungal taxa being split into multiple ASVs, we merged fungal ASVs which were assigned the same taxonomy into the same taxa. We chose to forego rarefaction of our samples as it increases uncertainty in relative abundances (McMurdie and Holmes, 2014).

We performed principal component analyses (PCAs) on the proportional abundances of taxa across PCR replicate and sample type (Supplementary Figures S3–S5). Taxa proportional abundances within samples were similar across PCR replicates, so read counts were summed across PCR replicates for each sample. There were ten salamander samples which grouped closely with wet swab and dry swab negative controls in the 16S PCAs on sample type, so these samples were removed from the 16S data for all subsequent analyses. Following Harrison et al. (2021), we used synthgene read counts to calculate the amount of microbial DNA in each sample relative to the synthgene (i.e., microbial read count divided by synthgene read count), and we compared the amount of microbial DNA in field samples to their associated negative controls (Supplementary Figure S6). Synthgenes were subsequently removed from the datasets. The final datasets for our field samples contained 15,690 bacterial taxa (6,529 for salamander, 8,873 for water, and 14,591 for substrate) and 469 fungal taxa (289 for salamander, 224 for water, and 413 for substrate).

### Water quality between sites and through time

To examine how water quality changed throughout the warm season, we fit a linear mixed-effects model for each water quality parameter (i.e., temperature, pH, conductivity, and dissolved oxygen [ppm and %]) using the lmerTest R package (version 3.1.3; Kuznetsova et al., 2017). In these linear mixed-effects models, stratum was treated as a random effect, and site, week, and their interaction were included as fixed effects. Stratum was coded with nine values representing the four strata in Gibson Lakes and the five strata in Ponds Lake. Site was treated as a categorical predictor, and week was treated as a continuous predictor. Week represented the number of weeks since June 9th, 2018. Water quality measurements are displayed in Supplementary Figure S7.

### Predicting *Bd*-inhibitory function

A database of amphibian skin-associated microbiome *Bd*-inhibitory bacterial isolates (Woodhams et al., 2015) was used to predict which bacteria observed in our datasets exhibit *Bd*-inhibitory properties (see Supplementary material for details). We trimmed sequences in the database of Woodhams et al. (2015) to the 16S rRNA V4 region using our 16S amplification primers with R, and we aligned the MiSeq 16S sequences of taxa detected in our NextSeq 16S field samples with the Woodhams et al. (2015) sequences using Clustal Omega (version 1.2.4; Sievers et al., 2011). We used FastTree 2 (version 2.1.11; Price et al., 2010) to create a phylogenetic tree, and we used stochastic character mapping with the make.simmap function in the phytools package (version 0.7.70; Revell, 2012) to predict the *Bd*-inhibition statuses of our observed taxa. Stochastic character mapping extends ancestral state reconstruction to probabilistically predict unobserved traits at the tips of a phylogenetic tree (Bollback, 2006). While existing applications of the Woodhams et al. (2015) database tend to employ local alignment or clustering methods to classify bacterial taxa as “potentially” *Bd*-inhibitory (e.g., Kueneman et al., 2016b; Bletz et al., 2017a; Kruger, 2020), stochastic character mapping provides the benefit of yielding probabilistic predictions that bacterial taxa are actually *Bd*-inhibitory. We further note that extended ancestral trait reconstruction is commonly applied in predicting the metabolic function of gut microbiomes (Langille et al., 2013). We visualized our phylogenetic tree with the posterior probabilities of our taxa being *Bd*-inhibitory using the Interactive Tree of Life (Supplementary Figure S8; version 6.5.4; Letunic and Bork, 2021).

The vast majority of our taxa had low confidence in their *Bd*-inhibition statuses (99.5% of posterior probabilities were between 47.9 and 52.5%), whereas most posterior probabilities which were < 40% or > 60% were also ≤10% or ≥ 90% (33 of 39). Therefore, we considered our bacterial taxa to be *Bd*-inhibitory if their posterior probabilities of *Bd*-inhibition were ≥ 90%, and we considered our bacterial taxa to be non-*Bd*-inhibitory if their posterior probabilities of *Bd*-inhibition were ≤ 10%. Otherwise, we considered our bacterial taxa to have an uncertain *Bd*-inhibition status.

### Microbial composition modeling

For both bacterial and fungal communities, we fit Bayesian Dirichlet-multinomial regression models to the salamander, water, and substrate microbiome data to identify differentially abundant microbes and to evaluate differences in overall community composition. Bayesian Dirichlet-multinomial regression estimates the effect of covariates on a set of proportions which sum to one (i.e., a simplex) and uses a set of counts as a multivariate response. In the context of microbiome data, the model uses read counts to estimate the expected proportional abundances of microbial taxa in the community, and the model considers the underlying uncertainty in each sample’s composition, which is dictated by the sample’s total read count (i.e., its sampling effort). Bayesian Dirichlet-multinomial models outperform other analyses of compositional data in detecting differences in community composition, and the model further allows for the identification of the taxa responsible for those differences (i.e., the model allows for differential abundance testing; Harrison et al., 2020).

Our Bayesian Dirichlet-multinomial regression model was adapted from the Bayesian Dirichlet regression model of Sennhenn-Reulen (2018), and we used backwards variable selection by widely applicable information criterion (WAIC) to optimize predictive accuracy. WAIC is a Bayesian analog to AIC and approximates the predictive accuracy of leave-one-out cross-validation (Gelman et al., 2014). In our model, sample read counts are distributed according to the Dirichlet-multinomial distribution. Each taxon receives a linear predictor combination, and the softmax function (a multivariate inverse logit) normalizes linear predictor combinations for all taxa into expected proportions. The last taxon serves as a reference category, and its intercept and regression coefficients are set to zero to allow for model identifiability. A precision parameter controls the degree of overdispersion relative to the multinomial distribution. See Supplementary material for model details.

Our Bayesian Dirichlet-multinomial regression models were computationally intensive to fit, with the number of model parameters and model run time increasing with the number of taxa included. To keep model run-times practical, we opted to select the 100 most proportionally abundant taxa from the salamander samples, plus an “other” category, for inclusion in the composition models. To select these taxa, we calculated the proportion of reads of each taxon in each salamander sample. For each barcode region (i.e., 16S or ITS), we then averaged each taxon’s proportion of reads by combinations of site and life stage. We then averaged across these averages, and we took the 100 taxa with the highest averaged proportion of reads for each barcode region for use in modeling. Other taxa which were not included in the top 100 for each barcode region had their read counts merged into an “other” category. By weighting each combination of site and life stage equally (i.e., by taking averages of averages) in selecting the top 100 taxa, we ensured that the top 100 taxa were not dominated by taxa from one site or life stage simply due to differences in sample size. Including the “other” category in the models ensures that the proportional abundances of the top 100 taxa remain unbiased. We chose to select the top 100 taxa because these comprise the vast majority of reads in salamander samples (93.1% of bacterial and 98.6% of fungal reads). As such, we expect variation in the composition of these taxa to represent most of the variation in community composition. Additionally, following initial testing, we deemed including 101 categories in the composition models (the top 100 taxa plus the “other” category) to be near the upper reasonable limit of our computing capacity on a high-performance computing cluster. Ultimately, our Bayesian Dirichlet-multinomial regression models, coupled with our backwards variable selection approach, took 785 CPU days to run. Datasets used in the modeling of water and substrate microbial communities included the same taxa as used for the salamander modeling, plus their own “other” categories. Proportional abundance estimates from Bayesian Dirichlet-multinomial regression models for water and substrate are later used to identify microbes which are disproportionately abundant on salamander skin relative to the environment. Since not all top microbial taxa in the salamander samples were detected in the water and substrate samples, the water and substrate datasets used in modeling had fewer than 101 taxa.

As water quality was highly correlated with space and time (Supplementary Figure S7), we fit models with two different sets of predictors. One predictor set included spatiotemporal covariates, while the other predictor set substituted spatiotemporal covariates with water quality. The spatiotemporal predictor set included four-way interactions between age, life stage, site, and a second-degree polynomial for week, all lower-level interactions, and the individual predictors. Stratum was also included as a predictor and treated as a hierarchical effect. The water quality predictor set included a five-way interaction between age, life stage, temperature (°C), pH, and dissolved oxygen (ppm), all lower-level interactions, and the individual predictors. Models for water and substrate lacked age and life stage predictors. Site and life stage were treated as categorical predictors, and age and week were treated as continuous predictors. Age took whole integers from zero (age-0) to two (age-2+), and week represented the number of weeks since June 9th, 2018.

Bayesian Dirichlet-multinomial regression models were fit in Stan (version 2.21.0; Carpenter et al., 2017) using the rstan R interface (version 2.21.2; Stan Development Team, 2020) with four Hamiltonian Monte Carlo (HMC) chains, 500 warmup iterations, 500 sampling iterations, and no thinning. Stan was chosen for its efficient HMC algorithm, and HMC chains were run in parallel on a University of Utah high-performance computing cluster. Gelman-Rubin convergence diagnostics ($\overset{\wedge }{R}$) and trace plots of the posteriors were used to assess model convergence. Since interpreting the effect of Dirichlet-multinomial regression coefficients on proportional abundances is not straightforward (see Supplementary material for a discussion), we opted for a graphical interpretation of the best-fit models (i.e., the models selected by backwards variable selection). We generated posterior predictions of proportional abundances for each combination of non-stratum predictors observed in the datasets, where predictions were for the average stratum (see Supplementary material for prediction details). The posterior predictions of taxa proportional abundances were summarized with 95% credible intervals, 50% credible intervals, and their median values.

For salamander microbiomes, Hill’s diversity index with *α* = 2 (Haegeman et al., 2013) was derived from the posterior predictions of taxa proportional abundances. By treating Hill’s diversity as a derived parameter from the Bayesian Dirichlet-multinomial regression models, we propagated the uncertainty associated with taxa proportional abundances to our diversity index. We chose Hill’s diversity (*α* = 2) as our alpha diversity index because it is insensitive to the many rare taxa expected in microbial communities, and can therefore be robustly estimated from microbiome data (Haegeman et al., 2013). Because of its insensitivity to rare taxa, we expected our grouping of rare taxa into an “other” category in the Bayesian Dirichlet-multinomial regression models to have a negligible impact on Hill’s diversity. We tested this expectation in the context of our data as follows. By grouping rare taxa into an “other” category, we created a situation of maximum unevenness within the group (i.e., the entire abundance of the “other” category was composed of a single taxon). We evaluated the sensitivity of Hill’s diversity to rare taxa by considering how evenly distributing the abundance of the “other” category across all of its member taxa influenced the index. For each HMC sample, we split the proportional abundance predictions of the “other” category into its individual members with uniform proportional abundances. We then re-derived Hill’s diversity and compared the posterior medians with the original values. After excluding a single combination of stage class and sampling event for bacterial communities, we observed a very strong correlation in Hill’s diversity estimates between the two methods (Pearson correlation coefficient of >0.999 for both bacteria and fungi). The excluded combination of stage class and sampling event was age-0 metamorphosed salamanders at Gibson Lakes on September 15th, which had a high proportional abundance of “other” bacterial taxa (posterior median of 0.196) and whose diversity estimate was found to be sensitive to the grouping of rare taxa into an “other” category (Hill’s diversity increased by 226.6% in the described test). We removed this combination of stage class and sampling event from our Hill’s diversity estimates for bacteria so that all remaining diversity estimates were reliable. The posterior distributions of Hill’s diversity were summarized with 95% credible intervals, 50% credible intervals, and their median values.

To estimate the *Bd*-inhibitory function of bacterial communities, we summed the read counts of bacteria belonging to each *Bd*-inhibition category within each sample, and we fit additional Bayesian Dirichlet-multinomial regression models for salamander, water, and substrate samples with three response categories representing the *Bd*-inhibition statuses (i.e., *Bd*-inhibitory, non-*Bd*-inhibitory, and uncertain *Bd*-inhibition status). These models used the spatiotemporal predictor set, and backwards variable selection by WAIC was again used to optimize predictive accuracy. We again generated posterior predictions of proportional abundances for each combination of non-stratum predictors observed in the datasets, where predictions were for the average stratum. We summarized the proportional abundances of each *Bd*-inhibition category with 95% credible intervals, 50% credible intervals, and their median values.

To examine which taxa and *Bd*-inhibition categories were disproportionately abundant on salamander skin relative to the environment, we considered the proportional abundance of microbes that salamanders experience in their environments to be a mixture between water and substrate proportional abundances, with the mixing ratio being a product of salamander behavior. Although we do not know this ratio, we expect that the result of this mixture is between the lower of the 0.025 quantiles (the lower ends of the 95% credible intervals) and the upper of the 0.975 quantiles (the upper ends of the 95% credible intervals) of the proportional abundance posterior predictions from water and substrate Bayesian Dirichlet-multinomial regression models, and we consider this range to represent the proportional abundance of a taxon or *Bd*-inhibition category in the environment. In determining this range, if a taxon was not detected in the water or substrate samples, and therefore was not included in the Bayesian Dirichlet-multinomial regression modeling for that sample type, it was considered to have 0.025 and 0.975 quantiles of proportional abundance predictions for that sample type of zero. The end result is that, if the proportional abundance of a taxon on salamander skin is higher than this range, then the taxon is disproportionately more abundant on salamander skin compared to both water and substrate. Conversely, if the proportional abundance of a taxon on salamander skin is lower than this range, then the taxon is disproportionately more abundant in both water and substrate than on salamander skin.

### Relationship between *Bd*-inhibitory bacteria and *Bd*

We detected *Bd* from ITS amplicon sequencing (i.e., there were fungal microbiome reads which were classified as *Bd*) on salamander skin at both lakes, and since *Bd* was absent in all negative control samples, we are confident that this was not the result of contamination. To verify that fungal microbiome reads which the naïve Bayesian classifier assigned to *Bd* were likely classified correctly, we performed an online nucleotide BLAST search (Zhang et al., 2000) with default settings for each of the 25 *Bd* ASVs which were previously merged into the *Bd* taxon. For each *Bd* ASV, the best-matching BLAST hit (the match with the lowest *E*-value) was a reference sequence belonging to *Bd*. 92% of *Bd* ASVs had 96.7% similarity or greater compared to their best-matching *Bd* reference sequence, and all ASVs had at least 94.5% similarity compared to their best-matching *Bd* reference sequence.

We tested for a relationship between the relative abundances of *Bd*-inhibitory bacteria and *Bd* on the skin of metamorphosed salamanders by fitting a Bayesian beta-binomial regression model with a logit link. We restricted this analysis to metamorphosed individuals because cutaneous *Bd* infections do not typically produce disease in larval amphibians (Marantelli et al., 2004). In our samples, *Bd* was detected on the skin of only 2.2% of larval or neotenic individuals (3 of 139) compared to 57.6% of metamorphosed individuals (38 of 66). The Bayesian beta-binomial regression model can be viewed as a univariate version of our earlier Bayesian Dirichlet-multinomial regression model. The Bayesian beta-binomial regression accounts for uncertainty in response values (i.e., the proportional abundance of *Bd* in fungal communities) by considering *Bd* read counts to be beta-binomially distributed, and a precision parameter controls the degree of overdispersion relative to the binomial distribution. Our model additionally accounts for uncertainty in the predictor values (i.e., the proportional abundance of *Bd*-inhibitory bacteria in bacterial communities) by estimating predictor values within the model from *Bd*-inhibitory read counts. Within the model, *Bd*-inhibitory read counts are beta-binomially distributed, and the logit linearizes *Bd*-inhibitory proportional abundance estimates for use as predictor values. See Supplementary material for model details. The model was fit in JAGS (version 4.3.0; Plummer, 2003) using the rjags R interface (version 4.10; Plummer, 2019) with three Markov chain Monte Carlo (MCMC) chains, 20,000 adaptation iterations, 20,000 warmup iterations, 100,000 sampling iterations, and no thinning. Gelman-Rubin convergence diagnostics and trace plots of the posteriors were used to assess model convergence.

## Results

### Field sampling

**Figure 2:**
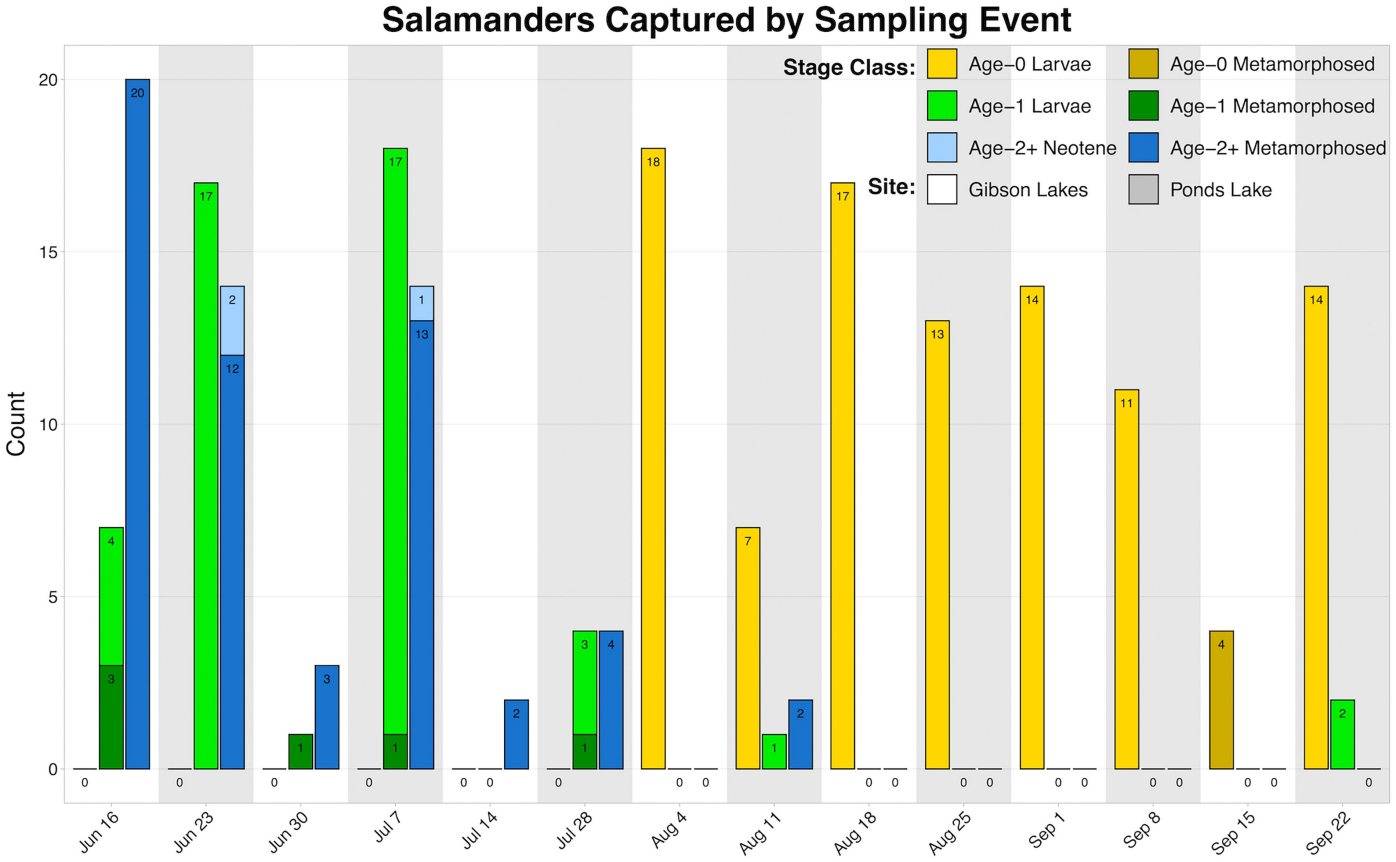
Counts of salamander skin-associated microbiome samples through time.

We observed higher amounts of microbial DNA in field samples compared to their associated negative controls (Supplementary Figure S6). Total sample counts are included in Table 1, and a breakdown of salamander skin-associated microbiome samples are displayed in Figure 2. The residency of different salamander age classes varied through time, and age-0 salamanders were too small to sample during the early warm season.

**Table 1:** Microbiome sample counts.

Format	Type	Count
Swab	Salamander	207
	Wet negative control	11
	Dry negative control	13
Water	Sample	60
	Negative control	20
Substrate	Sample	67
Blank	Negative control	12
Mock community	Positive control	2
Total		392

Based on observed sizes of males and females, most age-2+ salamanders – and only age-2+ salamanders – are thought to have been adults. Males develop swollen cloacas once sexually mature (Stebbins, 2003), and only one non-male age-2+ salamander (83 mm SVL; assumed to be female) had an SVL less than the smallest male (84 mm), with other small age-2+ salamanders in the range of 85 to 87 mm SVL being a mix of males (3) and females (4). Given the overlap in size between males and females, few subadults are expected to have been included in the age-2+ age class since male salamanders of this size were showing clear signs of sexual maturity. The absence of swollen cloacas from all age-0 and age-1 individuals suggests that only age-2+ individuals were sexually mature. 23 of 55 (41.8%) of sexed age-2+ salamanders were male (36.0% for Gibson Lakes and 46.7% for Ponds Lake).

Parameter estimates, test statistics, and *p*-values from the linear mixed-effects models for water quality are reported in Supplementary Table S1. These models included stratum as a random effect, and site, week, and their interaction were included as fixed effects. From the linear mixed-effects models, all water quality parameters (i.e., temperature, pH, conductivity, and dissolved oxygen [ppm and %]) changed significantly throughout the warm season (values of *p* ≤ 0.05 for all regression coefficients for week). We found a significant effect of site for all water quality parameters besides temperature, and we found significant interactions between site and week for conductivity and dissolved oxygen (both ppm and %). These results suggest that we were unable to detect differences in water temperature between Gibson Lakes and Ponds Lake, and temperature at both lakes decreased throughout the warm season (*β*_week_ = −0.713, value of *p* < 0.001). pH was lower at Ponds Lake compared to Gibson Lakes (*β*_site_ = −1.048, value of *p* < 0.001) and increased throughout the warm season at both lakes (*β*_week_ = 0.053, value of *p* < 0.001; non-significant interaction between site and week, value of *p* = 0.147). For conductivity and dissolved oxygen (both ppm and %), temporal trends were dependent on the lake (*p*-values ≤0.05 for all site, week, and interaction regression coefficients). Field measurements of water quality are shown in Supplementary Figure S7.

### Predictions of *Bd*-inhibitory function

Only 33 of the 15,690 taxa detected in our NextSeq 16S field samples were classified as *Bd*-inhibitory or non-*Bd*-inhibitory (i.e., posterior probabilities ≥90% or ≤ 10%; Supplementary Table S2). Of the 872 Woodhams et al. (2015) sequences which were used in the alignment, there were 361 unique sequences, and 79 of these unique sequences occurred across multiple bacterial isolates. We note that 41 of these 79 sequences (51.9%) had inconsistent *Bd*-inhibition statuses (i.e., statuses varied across isolates associated with the same sequence). We also note that the aligned Woodhams et al. (2015) sequences provided limited phylogenetic coverage of the bacterial taxa detected in our field samples (Supplementary Figure S8), with only 5,330 (34.0%) of our bacterial taxa belonging to phyla included in the Woodhams et al. (2015) database.

### Microbial composition

**Figure 3:**
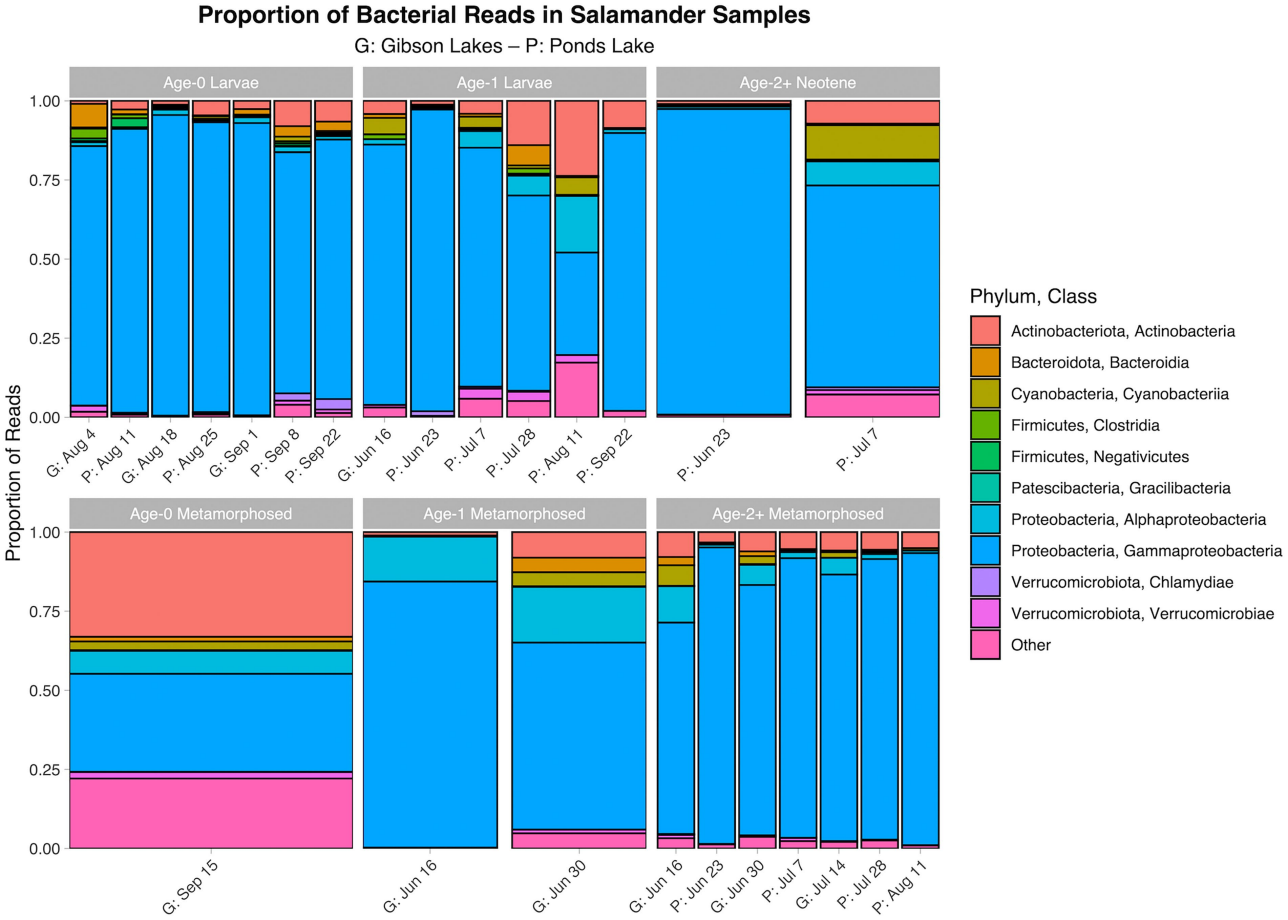
Proportion of bacterial reads in salamander samples belonging to each class. The ten classes with the highest number of salamander sample reads are displayed along with a category for the other classes. A stacked bar chart is displayed for each combination of stage class and sampling event, and each stacked bar chart represents reads pooled across samples belonging to the combination of stage class and sampling event. A “G” proceeds the dates of sampling events at Gibson Lakes, and a “P” proceeds the dates of sampling events at Ponds Lake.

**Figure 4:**
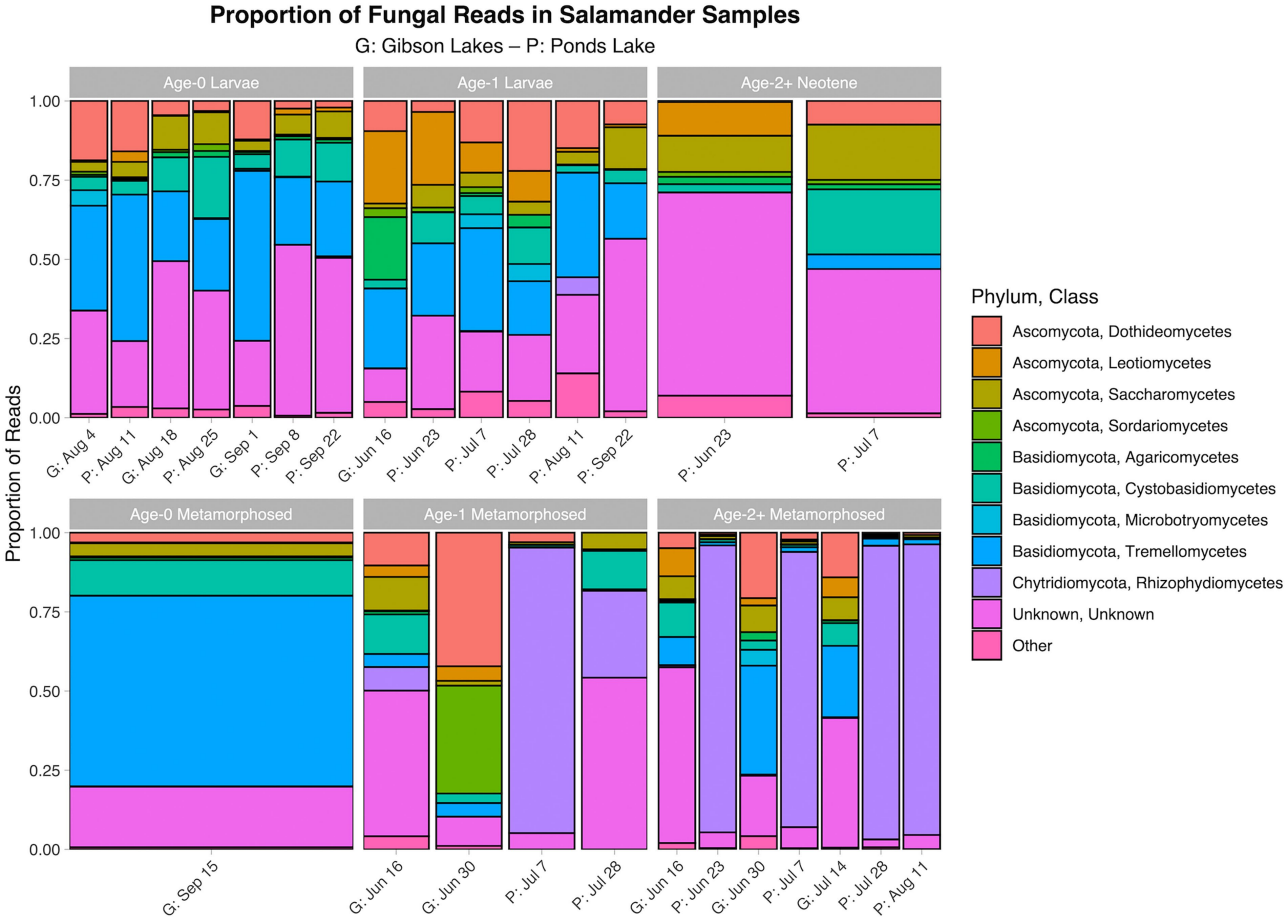
Proportion of fungal reads in salamander samples belonging to each class. The ten classes with the highest number of salamander sample reads are displayed along with a category for the other classes. A stacked bar chart is displayed for each combination of stage class and sampling event, and each stacked bar chart represents reads pooled across samples belonging to the combination of stage class and sampling event. A “G” proceeds the dates of sampling events at Gibson Lakes, and a “P” proceeds the dates of sampling events at Ponds Lake.

The proportion of reads in salamander samples belonging to each microbial class for each combination of stage class and sampling event are displayed in Figures 3, 4 for bacterial and fungal communities, respectively. Salamander bacterial communities were dominated by members of the phylum Proteobacteria and class Gammaproteobacteria (Figure 3), which comprised 89.8 and 86.7% of reads, respectively. The proportion of reads belonging to each fungal class (Figure 4) were more balanced than bacteria. Among salamander fungal reads, 41.6% belonged to class Rhizophydiomycetes, 21.4% were unidentified fungi, and 15.2% belonged to class Tremellomycetes.

Bayesian Dirichlet-multinomial regression models with spatiotemporal predictors fit better than models with water quality predictors for salamander samples (Table 2), suggesting that our spatiotemporal predictors were better able to predict salamander microbial composition. All but two of the best-fitting Bayesian Dirichlet-multinomial regression models with spatiotemporal predictors included stratum as a predictor, suggesting compositional variation in microbial communities within the lakes, with the models for salamander fungal communities and water *Bd*-inhibition categories being the exceptions. Except for stratum in the aforementioned models, all best-fitting spatiotemporal Bayesian Dirichlet-multinomial regression models included all individual predictors or their interactions, suggesting that all of our measured variables contributed to our ability to predict the composition of microbial communities.

**Table 2:** Predictors included in the best-fitting Bayesian Dirichlet-multinomial regression models for microbiome composition.

Sample type	Microbial community	Predictor set	Best-fit model predictors	Best-fit model WAIC	Full model WAIC	Number of models fit	Number of taxa included	Total CPU days
Salamander	Bacterial	Spatiotemporal	Stratum + Age + Life Stage + Site + Age:Life Stage + Age:Site + Life Stage:Site + Life Stage:Week + Site:Week + Site:Week^2^ + Age:Life Stage:Site + Age:Life Stage:Week + Age:Site:Week + Life Stage:Site:Week^2^ + Age:Life Stage:Site:Week^2^	140041.5	140505.6	196	101	105.7
		Water quality	Age + Life Stage + Life Stage:Temperature + Life Stage:pH + Temperature:pH + Life Stage:DO (ppm) + Age:Life Stage:pH + Age:pH:DO (ppm) + Age:Life Stage:Temperature:DO (ppm) + Life Stage:Temperature:pH:DO (ppm)	140706.1	141839.3	452	101	253.9
	Fungal	Spatiotemporal	Life Stage + Life Stage:Site + Age:Week^2^ + Site:Week^2^	120600.2	121624.9	295	101	161.9
		Water quality	Age + Life Stage + Life Stage:Temperature + Life Stage:DO (ppm) + Age:Life Stage:pH + Age:Life Stage:pH:DO (ppm) + Age:Temperature:pH:DO (ppm)	121168.6	122711.0	476	101	257.1
	*Bd*-inhibition categories	Spatiotemporal	Stratum + Life Stage + Week + Week^2^ + Age:Life Stage + Age:Site + Life Stage:Site + Age:Week + Age:Week^2^ + Life Stage:Week + Life Stage:Week^2^ + Site:Week + Site:Week^2^ + Age:Life Stage:Site + Age:Life Stage:Week + Age:Life Stage:Week^2^ + Age:Site:Week + Age:Site:Week^2^ + Life Stage:Site:Week + Life Stage:Site:Week^2^ + Age:Life Stage:Site:Week + Age:Life Stage:Site:Week^2^	4998.5	5005.0	70	3	1.6
Water	Bacterial	Spatiotemporal	Stratum + Site + Week + Site:Week + Site:Week^2^	44272.9	44329.3	12	87	0.7
	Fungal	Spatiotemporal	Stratum + Week + Week^2^ + Site:Week + Site:Week^2^	29359.9	29392.3	12	68	0.6
	*Bd*-inhibition categories	Spatiotemporal	Site + Week + Site:Week + Site:Week^2^	1673.2	1676.1	16	3	0.1
Substrate	Bacterial	Spatiotemporal	Stratum + Site + Week + Site:Week + Site:Week^2^	34202.2	34440.6	12	83	1.0
	Fungal	Spatiotemporal	Stratum + Week + Site:Week + Site:Week^2^	51644.8	51924.2	16	92	2.1
	*Bd*-inhibition categories	Spatiotemporal	Stratum + Site + Week + Site:Week + Site:Week^2^	1391.7	1400.6	12	3	0.1

Best-fit models are those selected by backwards variable selection by WAIC, and the full model is the initial model fit during backwards variable selection which includes all predictors. DO is dissolved oxygen.

The ten Bayesian Dirichlet-multinomial regression models from the backwards variable selection process with the lowest WAIC values for each sample type (i.e., salamander, water, or substrate), predictor set (i.e., spatiotemporal or water quality), and microbial community type (i.e., bacterial community, fungal community, or *Bd*-inhibition categories) are included in Supplementary Tables S3–S7. Four best-fitting Bayesian Dirichlet-multinomial regression models had other models within two WAIC (i.e., the model for substrate bacterial community composition and models for salamander, water, and substrate *Bd*-inhibition categories). With one exception, all other models within two WAIC contained the same predictors or their interactions as the best-fitting models. For example, a model with age as a predictor and another model with an interaction between age and site both contain age. The exception was one model within two WAIC of the best-fitting model for *Bd*-inhibition categories in lake water, which included stratum as a predictor while the best-fitting model excluded it.

Throughout our results, we consider non-overlapping 95% credible intervals of posterior predictions to represent differences in taxa proportional abundances, alpha diversity, or the proportional abundances of *Bd*-inhibition categories, depending on the analysis. These posterior predictions are all from the best-fitting Bayesian Dirichlet-multinomial regression model (the model with the lowest WAIC) for salamander, water, or substrate samples for bacterial communities, fungal communities, or *Bd*-inhibition categories. The 95% credible intervals of posterior predictions are presented in the referenced figures.

**Figure 5:**
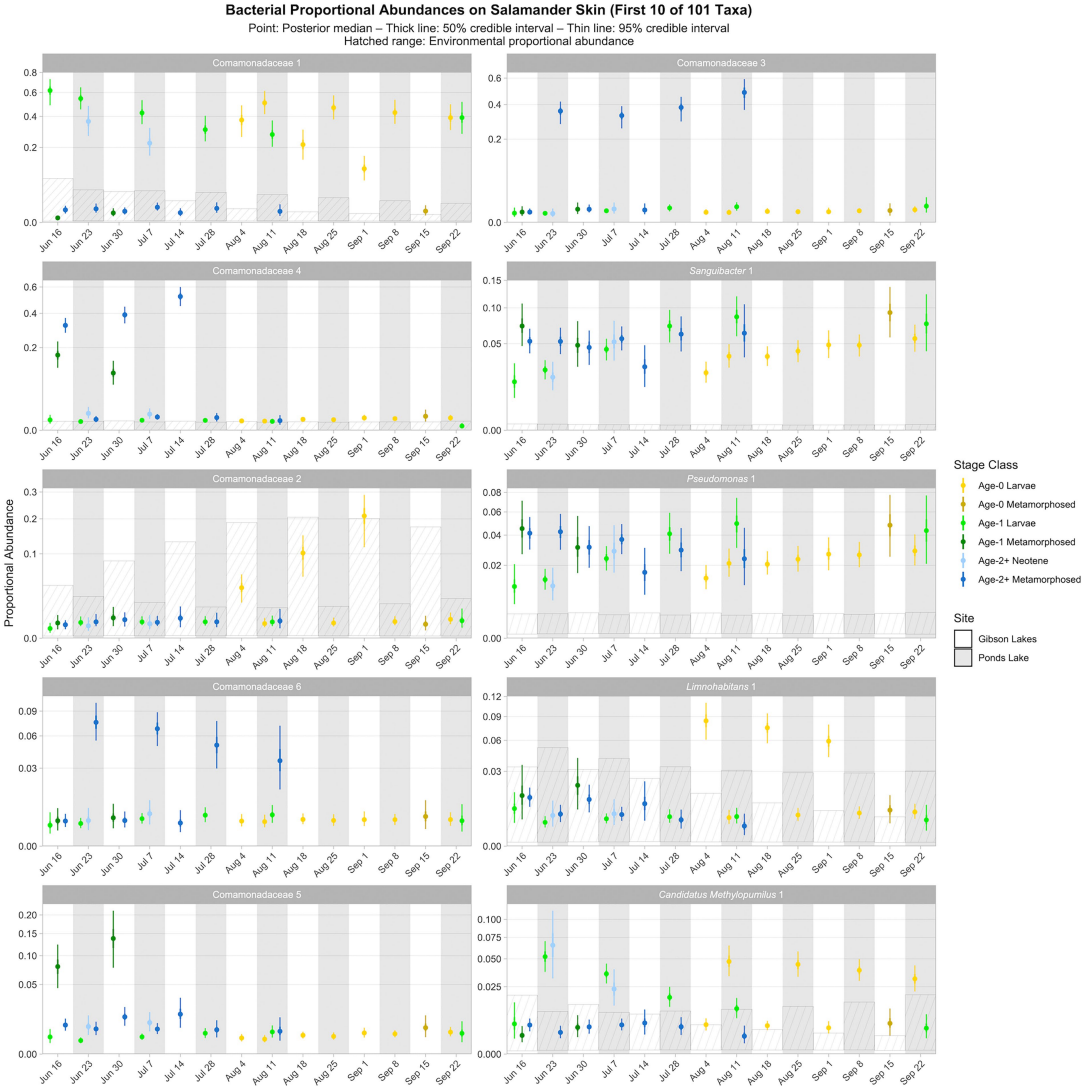
Proportional abundance predictions from Bayesian Dirichlet-multinomial regression models for the first ten bacterial taxa from the top 100. Points, thick lines, and thin lines represent posterior medians, 50% credible intervals, and 95% credible intervals, respectively. Hatched ranges represent environmental proportional abundances. Taxa without hatched ranges were not detected in either water or substrate. Note the square root scale on the *y*-axis. Taxa are ordered (left to right, top to bottom) by descending average proportion of reads in salamander samples in which each combination of site and life stage receives equal weight. Proportional abundance prediction plots for the remaining top 100 bacterial taxa can be found in the Supplementary material.

The following results are from the posterior predictions of the best-fitting Bayesian Dirichlet-multinomial regression model for bacterial community composition on salamander skin. We observed temporal, spatial, and ontogenetic variation in the proportional abundances of the top 100 bacterial taxa (Figure 5; Supplementary Figures S9–S13), with the degree of variation depending on the taxon. Proportional abundance trends were often taxon-specific, although patterns were observed across some taxa. Examples of temporal variation include an increase in the proportional abundance of Comamonadaceae 2 (i.e., the second ASV classified as Comamonadaceae) through time in Gibson Lakes age-0 larvae and a decrease in the proportional abundance of *Candidatus Methylopumilus* 1 through time in Ponds Lake age-1 larvae. Ontogenetic variation is apparent among many of the top 100 bacterial taxa. For example, the proportional abundances of Comamonadaceae 3 and 6 in Ponds Lake were consistently higher for age-2+ metamorphosed salamanders than other stage classes. In Gibson Lakes age-0 individuals, we observed higher proportional abundances of certain bacterial taxa on larvae followed by a sharp decline post-metamorphosis (e.g., Comamonadaceae 2, *Limnohabitans* 1, *Methylotenera* 7, *Methylotenera* 2, and *Sphingorhabdus rigui* 1).

Ninety of the top 100 bacterial taxa detected in salamander samples were also detected in environmental samples. We detected 86 and 82 of the top 100 bacterial taxa in water and substrate samples, respectively. From the posterior predictions of the best-fitting salamander, water, and substrate Bayesian Dirichlet-multinomial regression models for bacterial community composition, 32 of the top 100 bacterial taxa were disproportionately more abundant on salamander skin relative to the environment for the majority of combinations of stage class and sampling event (Figure 5; Supplementary Figures S9–S13), including *Sanguibacter* 1 and Gracilibacteria 3. Taxa detected exclusively on salamander skin include *Pseudochrobactrum kiredjianiae* 1 and *Roseomonas* 2. None of the top 100 bacterial taxa had lower proportional abundances on salamander skin than in the environment for the majority of combinations of stage class and sampling event.

**Figure 6:**
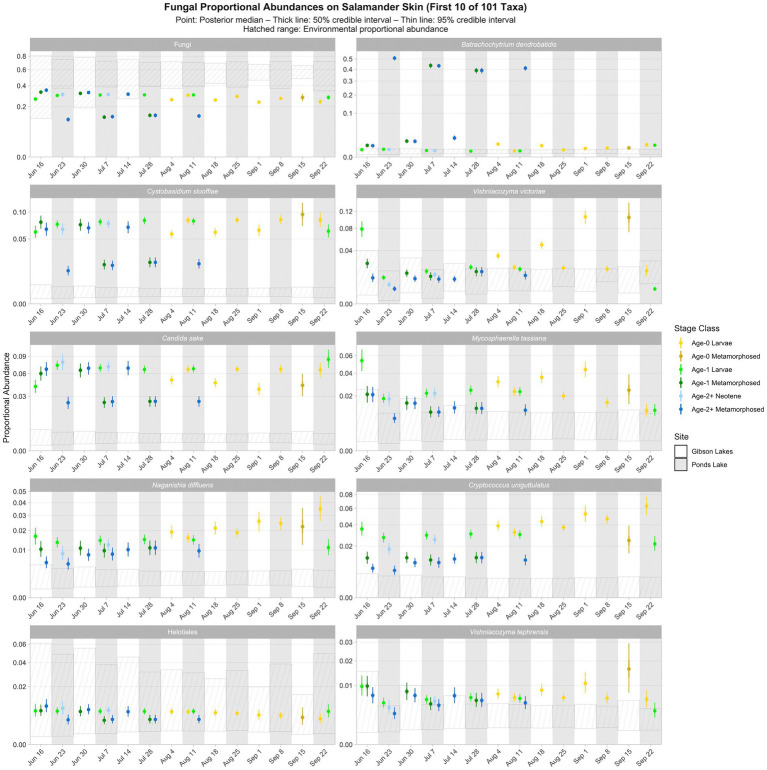
Proportional abundance predictions from Bayesian Dirichlet-multinomial regression models for the first ten fungal taxa from the top 100. Points, thick lines, and thin lines represent posterior medians, 50% credible intervals, and 95% credible intervals, respectively. Hatched ranges represent environmental proportional abundances. Note the square root scale on the *y*-axis. Taxa are ordered (left to right, top to bottom) by descending average proportion of reads in salamander samples in which each combination of site and life stage receives equal weight. Proportional abundance prediction plots for the remaining top 100 fungal taxa can be found in the Supplementary material.

As for bacteria, we observed spatiotemporal and ontogenetic variation in the proportional abundances of the top 100 fungal taxa based on the posterior predictions of the best-fitting Bayesian Dirichlet-multinomial regression model for fungal community composition on salamander skin (Figure 6; Supplementary Figures S14–S18). *Naganishia diffluens* experienced an increase in proportional abundance through time for age-0 and age-2+ salamanders at both lakes, and the proportional abundance of *Vishniacozyma victoriae* increased through time for Gibson Lakes age-0 larvae. Notably, the proportional abundance of *Bd* in Ponds Lake metamorphosed individuals was very high (between 35 and 55%) compared to Ponds Lake larvae and either life stage at Gibson Lakes (all <2.5%; Figure 6). In contrast, metamorphosed individuals at Ponds Lake had lower proportional abundances of *Cystobasidium slooffiae* compared to Ponds Lake larvae and either life stage at Gibson Lakes, the opposite of the pattern observed for *Bd*.

Ninety-three of the top 100 fungal taxa detected in salamander samples were also detected in environmental samples. We detected 67 and 91 of the top 100 fungal taxa in water and substrate samples, respectively. From the posterior predictions of the best-fitting salamander, water, and substrate Bayesian Dirichlet-multinomial regression models for fungal community composition, 27 of the top 100 fungal taxa were disproportionately more abundant on salamander skin relative to the environment for the majority of combinations of stage class and sampling event (Figure 6; Supplementary Figures S14–S18), including *Candida sake*, *Wallemia muriae*, and *Vishniacozyma*. Taxa detected exclusively on salamander skin include Pleosporales, *Melanodiplodia tianschanica*, and *Buckleyzyma aurantiaca*. Four of the top 100 fungal taxa had lower proportional abundances on salamander skin than in the environment for the majority of combinations of stage class and sampling event, including Ascomycota, Basidiomycota, and Rozellomycota.

**Figure 7:**
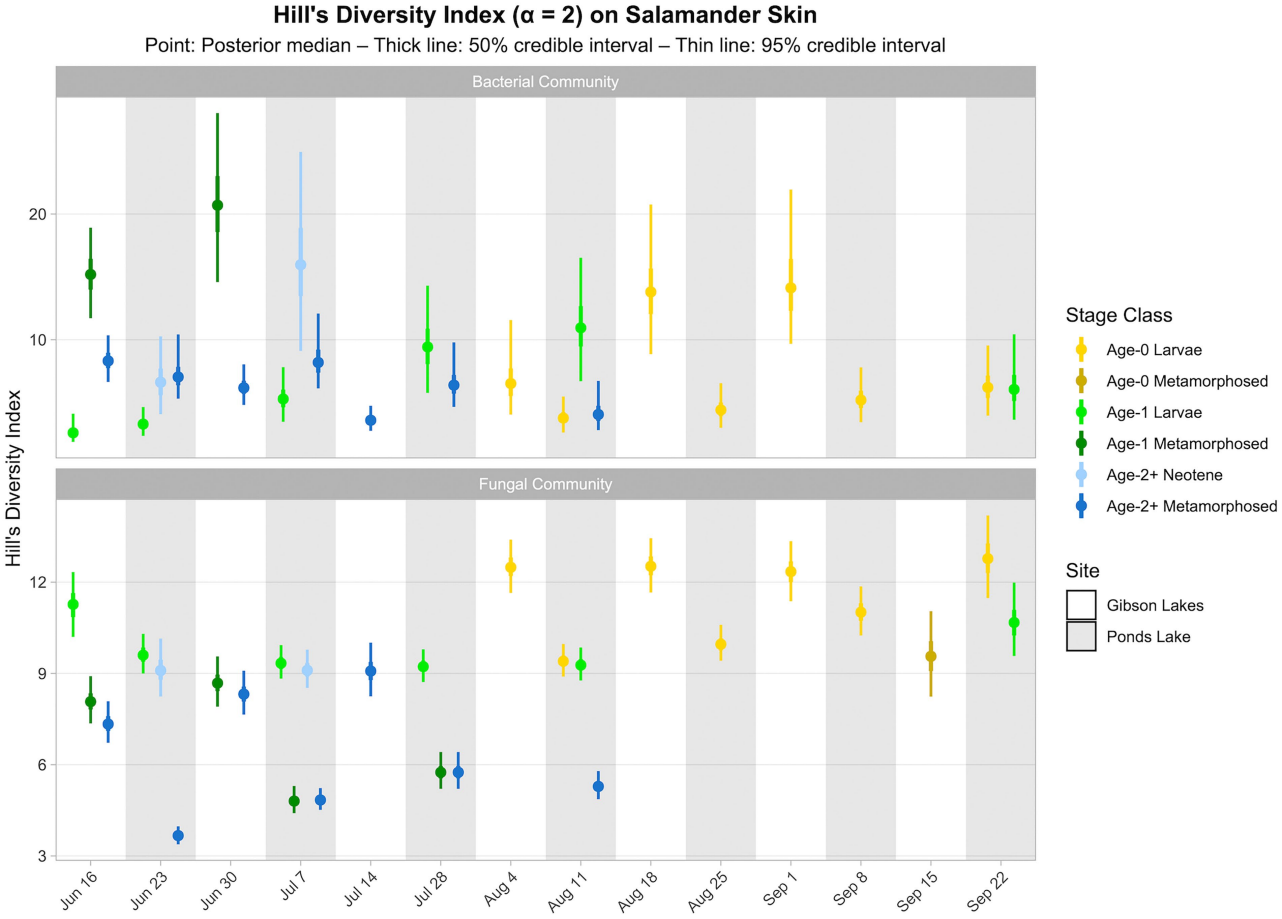
Hill’s diversity index (*α* = 2) for bacterial and fungal communities on salamander skin. Diversity estimates were derived from proportional abundance predictions of bacterial and fungal taxa from Bayesian Dirichlet-multinomial regression models. Points, thick lines, and thin lines represent posterior medians, 50% credible intervals, and 95% credible intervals, respectively. Note that bacterial diversity for age-0 metamorphosed salamanders at Gibson Lakes on September 15th is omitted because, for this combination of stage class and sampling event only, we found Hill’s diversity to be sensitive to our grouping of rare taxa (i.e., those not belonging to the top 100) into an “other” category in the Bayesian Dirichlet-multinomial regression models.

Salamander bacterial and fungal diversity (i.e., Hill’s diversity with *α* = 2 derived from the posterior predictions of the best-fitting Bayesian Dirichlet-multinomial regression models for salamander bacterial and fungal communities, respectively) are displayed in Figure 7. Salamander bacterial diversity had the highest values in early-season age-1 metamorphosed individuals at Gibson Lakes, age-2+ neotenes at Ponds Lake in July, and age-0 larvae after mid-August at Gibson Lakes (Figure 7). Bacterial diversity for age-1 larvae increased throughout the early warm season at Ponds Lake when most of this stage class was observed. Bacterial diversity in age-2+ metamorphosed individuals tended to decrease through time at both lakes. In late August and early September, bacterial diversity was higher for age-0 larvae at Gibson Lakes than Ponds Lake. Patterns of microbial diversity for fungi differed than those for bacteria. Salamander fungal diversity was highest among age-0 larvae at Gibson Lakes, age-1 larvae at the beginning of the warm season at Ponds Lake, and late-season larvae at Ponds Lake (Figure 7). Fungal diversity increased throughout the early warm season for metamorphosed individuals at both lakes, with metamorphosed individuals at Ponds Lake having lower fungal diversity than at Gibson Lakes (Figure 7). The lower fungal diversity for metamorphosed individuals at Ponds Lake compared to Gibson Lakes may be due to the high proportional abundance of a single fungal taxon – reducing species evenness – on Ponds Lake metamorphosed salamanders (the posterior medians of this taxon, *Bd*, ranged from 0.390 to 0.508; Figure 6).

**Figure 8:**
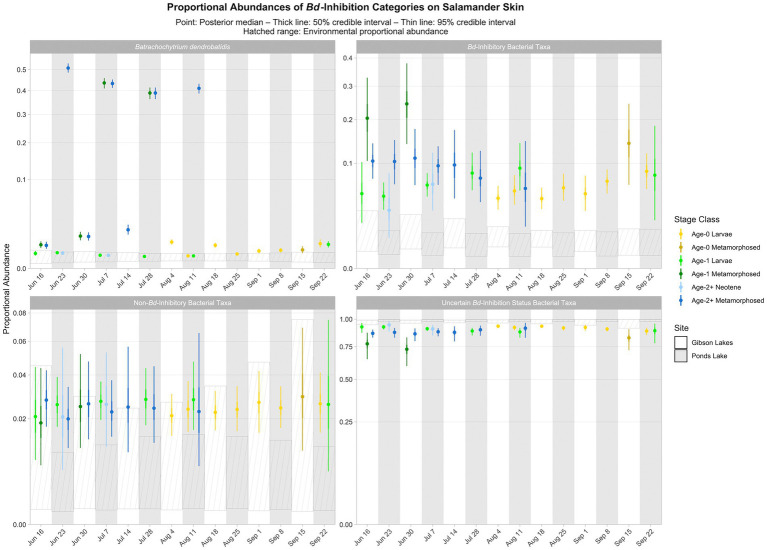
Proportional abundance predictions of *Batrachochytrium dendrobatidis* (*Bd*) and *Bd*-inhibitory bacterial taxa from Bayesian Dirichlet-multinomial regression models. Points, thick lines, and thin lines represent posterior medians, 50% credible intervals, and 95% credible intervals, respectively. Hatched ranges represent environmental proportional abundances. Note the square root scale on the *y*-axis.

Based on the best-fitting Bayesian Dirichlet-multinomial regression models for the composition of bacterial *Bd*-inhibition categories (i.e., *Bd*-inhibitory, non-*Bd*-inhibitory, and uncertain *Bd*-inhibition status), *Bd*-inhibitory taxa were disproportionately more abundant on salamander skin relative to the environment for most combinations of stage class and sampling event (23 of 25; Figure 8). Non-*Bd*-inhibitory bacterial taxa were disproportionately more abundant on salamander skin relative to the environment for most combinations of stage class and sampling event at Ponds Lake (10 of 15), but we were unable to detect differences in the proportional abundances of non-*Bd*-inhibitory bacterial taxa between salamander skin and the environment for any combination of stage class and sampling event at Gibson Lakes (Figure 8). Bacterial taxa of uncertain *Bd*-inhibition status were disproportionately more abundant in the environment compared to salamander skin for most combinations of stage class and sampling event (23 of 25; Figure 8).

## Relationship between *Bd*-inhibitory bacteria and *Bd*

**Figure 9:**
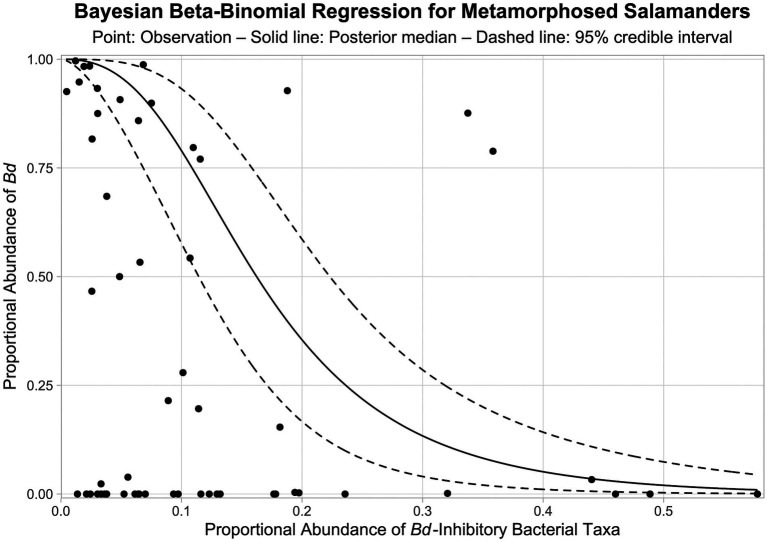
Bayesian beta-binomial regression between the proportional abundances of *Bd*-inhibitory bacteria and *Bd* in bacterial and fungal communities, respectively, on the skin of metamorphosed salamanders. Solid and dashed lines represent the medians and 95% credible intervals of posterior predictions, respectively. Points represent observations from metamorphosed salamanders. Note that there is uncertainty associated with both the response and predictor values (i.e., proportional abundances), and the Bayesian beta-binomial regression considers this uncertainty within the model.

From our Bayesian beta-binomial regression model, there is a > 99.9% probability that a negative relationship exists between the proportional abundance of *Bd*-inhibitory bacteria in bacterial communities and the proportional abundance of *Bd* in fungal communities on the skin of metamorphosed salamanders (i.e., > 99.9% of MCMC samples for the regression coefficient were negative). The posterior median of the regression coefficient was −2.402, and the 95% credible interval was −3.386 to −1.586. The modeled relationship between the relative abundances of *Bd*-inhibitory bacteria and *Bd* on the skin of metamorphosed salamanders is shown in Figure 9.

## Discussion

We observed spatiotemporal and ontogenetic variation in the relative abundances and microbial diversity of bacterial and fungal taxa in the skin-associated microbiome of the western tiger salamander at two high alpine Rocky Mountain lakes. Our best-fitting Bayesian Dirichlet-multinomial regression models for microbial community composition included all predictors or their interactions except for the models of fungal communities on salamander skin and *Bd*-inhibition categories in lake water, for which the stratum predictor was excluded (Table 2). Because rare taxa (i.e., not members of the top 100) were grouped into an “other” category in our Bayesian Dirichlet-multinomial regression models, variation in the relative abundances of these rare taxa were masked within changes in the relative abundance of the “other” category. Therefore, our results should be considered conservative estimates of the variation in microbial community composition on tiger salamander skin because variation within the “other” category was not considered. Because our top 100 taxa comprise the vast majority of reads in salamander samples (93.1% of bacterial and 98.6% of fungal reads), we expect variation in the composition of these taxa, plus the “other” category, to represent most of the variation in salamander microbiomes. When viewed conservatively, our models already suggest that all of our covariates (except for stratum in the aforementioned cases) contribute to our ability to predict microbial community composition, so we expect this outcome would be largely unaffected by the inclusion of additional taxa outside of the “other” category.

Our findings of variation in microbial community composition between sites, across life stages, and through time is consistent with the results of other studies (Kueneman et al., 2013; Longo et al., 2015; Bletz et al., 2017a,b). The inclusion of stratum as a predictor in the best-fitting composition model of bacteria on salamander skin, as well as for composition models of bacteria and fungi in the environment, suggests that we observed spatial variation in microbial community composition within lakes in addition to between lakes. Furthermore, the inclusion of salamander age as a predictor in the best-fitting models of bacterial and fungal community composition on salamander skin suggests that, within life stages, we also observed variation in microbial community composition with salamander age. For salamander skin-associated bacterial and fungal communities, composition was better explained by spatiotemporal than water quality covariates. In agreement with other amphibian skin-associated microbiome studies, we found that the skin of the western tiger salamander is a selective environment with taxa disproportionately represented compared to their relative abundances in water and substrate (Kueneman et al., 2013; Walke et al., 2014; Bletz et al., 2017a).

Time or its interactions were included as predictors in all best-fitting Bayesian Dirichlet-multinomial regression models for microbial community composition (Table 2), suggesting that microbial communities changed throughout the warm season. Notably, an interaction between time, salamander age, life stage, and lake was included in the best-fitting model for bacterial communities on salamander skin. Additionally, the best-fitting model for fungal communities on salamander skin included both an interaction between time and salamander age and an interaction between time and lake. This suggests that temporal trends in salamander bacterial and fungal community composition varied by lake and salamander stage class. While time was included as a predictor in the best-fitting model for *Bd*-inhibition categories on salamander skin, we failed to detect changes in the relative abundances of *Bd*-inhibitory bacteria through time for any stage class (i.e., within a stage class, all 95% credible intervals overlapped; Figure 8). This concurs with the findings of Bletz et al. (2017a), which found changes in bacterial community composition through time but stability in predicted *Bd*-inhibitory function. We are hesitant to draw the same conclusion, however, both because failing to detect change does not mean that change has not occurred, and because we could only confidently predict the *Bd*-inhibition status of a small minority of observed bacterial taxa. We did detect changes in the relative abundances of common bacteria (e.g., Comamonadaceae 2 and *Candidatus Methylopumilus* 1; Figure 5) and fungi (e.g., *Naganishia diffluens* and *Vishniacozyma victoriae*; Figure 6) through time on salamander skin. We also detected changes in bacterial and fungal diversity through time for stage classes at both lakes (Figure 7).

Similar to time, life stage or its interactions were also included as predictors in all best-fitting Bayesian Dirichlet-multinomial regression models for microbial community composition (Table 2). Notably, the proportional abundance of the bacterial taxon Comamonadaceae 1 was higher on the skin of non-metamorphosed salamanders (i.e., larval or neotenic individuals) than metamorphosed salamanders (i.e., 95% credible intervals did not overlap) throughout the warm season at both lakes (Figure 5). This taxon was very abundant on the skin of non-metamorphosed salamanders, typically comprising more than 20% of the bacterial community, and sometimes exceeding 40% (Figure 5). Kueneman et al. (2013) also observed a very high relative abundance (>65%) of a single member of Comamonadaceae on a life stage of the Cascades frog (*Rana cascadae*), but the taxon dominated the skin of metamorphosed frogs instead of tadpoles. In our study, Comamonadaceae 1 was also disproportionately more abundant on the skin of non-metamorphosed salamanders relative to the environment, whereas we were unable to detect differences in the proportional abundance of this taxon between the environment and the skin of metamorphosed salamanders (Figure 5). While we compared the proportional abundances of microbial taxa on salamander skin to environmental proportional abundances in lake water and lake substrate, we note that metamorphosed salamanders – although caught from the water – may have also had access to terrestrial sources of microbiota (e.g., soil) which we did not sample. For fungi, both the proportional abundance of *Cryptococcus uniguttulatus* and community diversity were higher on the skin of non-metamorphosed salamanders at every time point where both metamorphosed and non-metamorphosed salamanders were observed (Figures 6, 7). This contrasts with the findings of Kueneman et al. (2016b), in which microeukaryote diversity was higher on adult western toads (*Anaxyrus boreas*) than tadpoles.

We detected *Bd* on salamander skin at both lakes, with the relative abundance of *Bd* being highest for age-1 and age-2+ metamorphosed salamanders at Ponds Lake (Figure 6). The higher abundance of *Bd* on the skin of metamorphosed compared to larval amphibians is supported by other studies and is thought to be the result of increased keratin, a substrate for *Bd*, in amphibian skin following metamorphosis, during which structural changes to the skin occur (Berger et al., 1998; Marantelli et al., 2004; Frost et al., 2006). We are unsure why differences in the relative abundance of *Bd* was much less pronounced between larval and metamorphosed individuals at Gibson Lakes. Since *Bd* was absent in all negative control samples, we are confident that *Bd* was present at Gibson Lakes and that its detection was not the result of contamination from Ponds Lake samples.

We observed that *Bd*-inhibitory bacterial taxa were disproportionately more abundant on salamander skin relative to the environment for most combinations of stage class and sampling event (Figure 8). If bacterial taxa for which we have high confidence in their *Bd*-inhibition statuses can be considered a random sample from both salamander skin and the environment, then this could be taken as evidence that salamander skin selects for *Bd*-inhibitory bacteria. However, salamander skin also appeared to select for non-*Bd*-inhibitory bacteria at one lake, and for both lakes, bacteria of uncertain *Bd*-inhibition status were disproportionately more abundant in the environment than on salamander skin. Since environmental bacteria are not the focus of the Woodhams et al. (2015) database, we suspect that bacteria in this reference database are more likely to be common on amphibian skin than in the environment. This could result in more environmental bacteria having an uncertain *Bd*-inhibition status, and *Bd*-inhibitory and non-*Bd*-inhibitory bacteria would subsequently appear to be disproportionately more abundant on salamander skin than in the environment. Still, the apparent selection for *Bd*-inhibitory bacteria on salamander skin is stronger than for non-*Bd*-inhibitory bacteria (Figure 8), suggesting that selection for *Bd*-inhibitory bacteria may indeed be occurring. Despite harboring *Bd*, tiger salamanders have been found to tolerate chytridiomycosis (Davidson et al., 2003), and we suggest that selection for *Bd*-inhibitory bacteria by tiger salamander skin may contribute to this disease tolerance.

When viewed across combinations of stage class and sampling event, we did not observe any noticeable patterns between the relative abundances of *Bd*-inhibitory bacteria and *Bd* (Figure 8). That is, across combinations of stage class and sampling event, the relative abundance of *Bd* was not high or low when the relative abundance of *Bd*-inhibitory bacteria was high or low. We did, however, observe a negative pattern between the relative abundances of *Bd* and the fungal taxon *Cystobasidium slooffiae* (i.e., the relative abundance of *Bd* was low when the relative abundance of *Cystobasidium slooffiae* was high; Figure 6). Conversely, we observed positive patterns between the relative abundances of *Bd* and Comamonadaceae 3 and 6 (i.e., the relative abundance of *Bd* was high when the relative abundances of these taxa were high; Figures 5, 6). Comamonadaceae has been found to be abundant on the skin of multiple amphibian species, including the tiger salamander (McKenzie et al., 2011), and some members show evidence of *Bd*-inhibition or negative co-occurrence with fungal taxa (Woodhams et al., 2015; Kueneman et al., 2016b). Despite this, Walke et al. (2015) found a very weak correlation between a member of Comamonadaceae and *Bd*, and we found positive patterns between the relative abundances of members of Comamonadaceae and *Bd*. While we were unable to confidently predict the *Bd*-inhibition statuses of Comamonadaceae 3 and 6, we did predict one member of Comamonadaceae to be *Bd*-inhibitory (Comamonadaceae 5). Still, we observed no pattern between the relative abundances of this taxon and *Bd* (Figures 5, 6).

Within metamorphosed salamanders, we found strong evidence (> 99.9% probability from a Bayesian beta-binomial regression) of a negative relationship between the relative abundances of *Bd*-inhibitory bacteria and *Bd* in bacterial and fungal communities, respectively (Figure 9). We caution, however, that the mechanism behind this relationship is unclear from our data. We do not know whether *Bd*-inhibitory bacteria inhibit *Bd* growth, or if the opposite is true. Infection with *Bd* can lead to the restructuring of microbial communities on amphibian skin (Jani and Briggs, 2014; Jani and Briggs, 2018), and it is possible that *Bd* infection may directly or indirectly inhibit the growth of *Bd*-inhibitory bacteria. Our use of microbiome read counts to test for a relationship between *Bd*-inhibitory bacteria and *Bd* produced comparable results to studies which used quantitative PCR to detect and quantify the abundance of *Bd*. For example, Jiménez et al. (2022) found that *Bd* infection intensity significantly decreased on the skin of the eastern newt (*Notophthalmus viridescens*) as the relative abundance of putative *Bd*-inhibitory bacteria increased. Similarly, Flechas et al. (2019) found lower *Bd* infection prevalence within post-metamorphic life stages which also had high relative abundances of *Bd*-inhibitory bacteria in two frog species.

An analysis between the absolute abundances of *Bd*-inhibitory bacteria and *Bd*, instead of the relative abundances, would be of greater interest biologically. Following DNA extraction and prior to PCR, fixed amounts of 16S and ITS synthgenes (i.e., synthetic gene spike-ins) were added to a constant volume of each sample’s DNA extract. The synthgene read counts provide a benchmark to compare taxon read counts with, and can serve as the basis for absolute abundance estimation (Harrison et al., 2021). While we used synthgenes to estimate the amount of microbial DNA in our samples relative to negative controls, we were unable to use the synthgenes for estimating the densities (i.e., count per unit area) of microbial taxa on salamander skin because, as we were not aware of synthgenes at the time, we did not measure swabbed area in the field. Furthermore, a length-weight regression suggested that salamanders grow allometrically (i.e., the body does not grow proportionally in all dimensions; see Supplementary material), so an assumption about salamander growth would have to be made in order to derive surrogates of swabbed area from length measurements (i.e., SVL squared could not be used as a surrogate for swabbed area). We also considered limitations in our swabbing protocol. Since our study focused on variation in microbiome composition, we adopted the swabbing protocol of Bletz et al. (2017a), in which a swab is stroked across the ventral surface of the amphibian ten times (one time = an up and back stroke along the full length of the belly). While swabbing, the ten strokes along the length of the belly were distributed across the belly’s width. Due to the fixed size of the swab, this means that the same belly area was swabbed more times for smaller salamanders than for larger salamanders. This implies that even if we had measured swabbed area, we would have to assume that the number of microbes collected asymptotes after a certain swabbing intensity, and we must have further assumed that we reached this threshold of swabbing intensity. We suggest that the need for these assumptions can be avoided by using a different swabbing protocol. For example, instead of stroking a swab across the ventral surface a certain number of times while covering an area of interest, one could swab the full area of interest (e.g., the belly) a certain number of times and measure the swabbed area, a method which is already applied in studies of *Bd* load (North and Alford, 2008). If such a swabbing protocol were applied, we suggest that taxon density on amphibian skin could be modeled using a negative binomial regression for rates (i.e., taxon read count per unit “time”) – where rate represents taxon density, and synthgene read count and swabbed area serve as measures of “time.”

A key aim of amphibian skin-associated microbiome studies relates to understanding what role microbial communities play in protecting their hosts against cutaneous diseases such as chytridiomycosis. While DNA metabarcoding is commonly employed to characterize the composition of microbial communities, we experienced challenges relating community composition to functional activity. Using 16S rRNA gene sequences, we were unable to predict *Bd*-inhibition statuses for the vast majority of our bacterial taxa with any reasonable certainty. This is not surprising given that, after trimming to our amplicon region, the majority of sequences in the Woodhams et al. (2015) database which were shared across multiple bacterial isolates had variable *Bd*-inhibition statuses, and the isolates included in the database provided limited phylogenetic coverage of our bacterial taxa. Similarly, Becker et al. (2015) found bacterial congeners to frequently range from complete inhibition to facilitation of *Bd*. Another approach to exploring the functional activity of microbial communities involves metatranscriptomics, the sequencing of RNA within a microbiome to investigate gene expression (Nichols and Davenport, 2021). With a metatranscriptomics approach to exploring functional activity, antifungal secondary metabolite production by microbes experiencing real-world biotic and abiotic conditions on salamander skin could be observed, and a precise knowledge of community composition, while still informative, would not be a pre-requisite for inference.

Our study emphasizes two traditionally understudied areas of amphibian skin-associated microbial ecology, temporal variation in community composition and expanding our view of the microbiome to include fungi in addition to bacteria. Temporal variation in community composition could prove challenging for studies examining spatial variation, where temporal and spatial variation may be confounded. We also identified additional sources of variation in community composition which are not typically considered. Within life stages, we identified additional variation with salamander age, and within lakes, we identified additional variation between strata. Furthermore, we observed that the relationships between community composition and spatiotemporal and stage class covariates are interdependent, complex, and best described using interactions.

Through this study, we have gained a greater understanding of microbial ecology on amphibian skin through the examination of season-long temporal variation of bacterial and fungal communities. In addition to identifying further sources of variation in community composition, we have identified differentially abundant taxa, have examined microbial selection by salamander skin, have investigated alpha diversity, and have tested for a relationship between predicted *Bd*-inhibitory function and *Bd*. Ultimately, we hope our findings will assist in the conservation of amphibian species threatened by chytridiomycosis.

## Data availability statement

Raw sequence reads are deposited in the Sequence Read Archive (BioProject PRJNA843333). Sample metadata are available on DataDryad (doi: 10.5061/dryad.nzs7h44tw). Scripts used in the analyses are available on GitHub (https://github.com/Urodelan/2022_Salamander_Microbiome).

## Ethics statement

The animal study was reviewed and approved by the Utah State University Institutional Animal Care and Use Committee.

## Author contributions

KG and ZG designed the research. KG and JH conducted field sampling and wrote the manuscript with revisions from all authors. KG performed the lab work and analyses. ZG provided guidance, support, and feedback at all stages of the project. All authors contributed to the article and approved the submitted version.

## Funding

This project was supported with funding from the Utah State University (USU) Quinney College of Natural Resources Undergraduate Research Grant, the USU Office of Research Undergraduate Research and Creative Opportunities Grant, the USU Honors Program Honors Research Fund, the Society for the Study of Amphibians and Reptiles Roger Conant Undergraduate Research Grant in Herpetology, and the National Science Foundation (DEB 1638768 to ZG).

## Conflict of interest

The authors declare that the research was conducted in the absence of any commercial or financial relationships that could be construed as a potential conflict of interest.

## Publisher’s note

All claims expressed in this article are solely those of the authors and do not necessarily represent those of their affiliated organizations, or those of the publisher, the editors and the reviewers. Any product that may be evaluated in this article, or claim that may be made by its manufacturer, is not guaranteed or endorsed by the publisher.
