# Supplementary material for: Spatiotemporal and ontogenetic variation, microbial selection, and predicted Bd-inhibitory function in the skin-associated microbiome of a Rocky Mountain amphibian
Source: Front Microbiol. 2022 Dec 13;13:1020329. doi: 10.3389/fmicb.2022.1020329 (PMC9792605; doi:10.3389/fmicb.2022.1020329)
Supplement: Supplementary file 1 [file Data_Sheet_1.pdf]

Supplementary Material

Table of contents

Methods 2
SNOTEL snowmelt timing data 2
Salamander length and weight 2
Library preparation 2
DADA2 bioinformatics pipeline 3
Creating study-specific reference libraries 4
Mock community visualization 5
Quality assessment of microbiome data 5
Predicting *Bd*-inhibitory function 9
Microbial composition modeling 10
Microbial composition predictions 13
Relationship between the relative abundances of *Bd*-inhibitory bacteria and *Bd* 14
Salamander length-weight regression 15
Results 15
Water quality 15
Predictions of *Bd*-inhibitory function 18
Top ten Bayesian Dirichlet-multinomial regression models 20
16S proportional abundance plots 24
ITS proportional abundance plots 29
Model code 34
Stan code for Bayesian Dirichlet-multinomial regression model 34
JAGS code for Bayesian beta-binomial regression model 37
References 38

Methods

SNOTEL snowmelt timing data

Although Ponds Lake was not visited early enough to observe salamander eggs, snowmelt data suggests that salamanders likely laid eggs at about the same time at both study sites. Data from a NRCS SNOTEL site (#828) within ~1.2 km distance and ~50 m elevation from Ponds Lake first reported a 2018 snow water equivalent of zero on May 24th, and data from a SNOTEL site (#484) within ~2.9 km and ~80 m elevation from Gibson Lakes first reported a 2018 snow water equivalent of zero on May 23rd. The 30-year median snow water equivalent reaches zero at the SNOTEL sites near Ponds Lake and Gibson Lakes on June 11th and June 4th, respectively. Taken together, this suggests that snow melted at these lakes at about the same time in 2018, possibly within days of each other, and snow typically melts at these lakes about a week apart.

Salamander length and weight

Salamander length and weight measurements were taken to verify age classes (Figure S1).

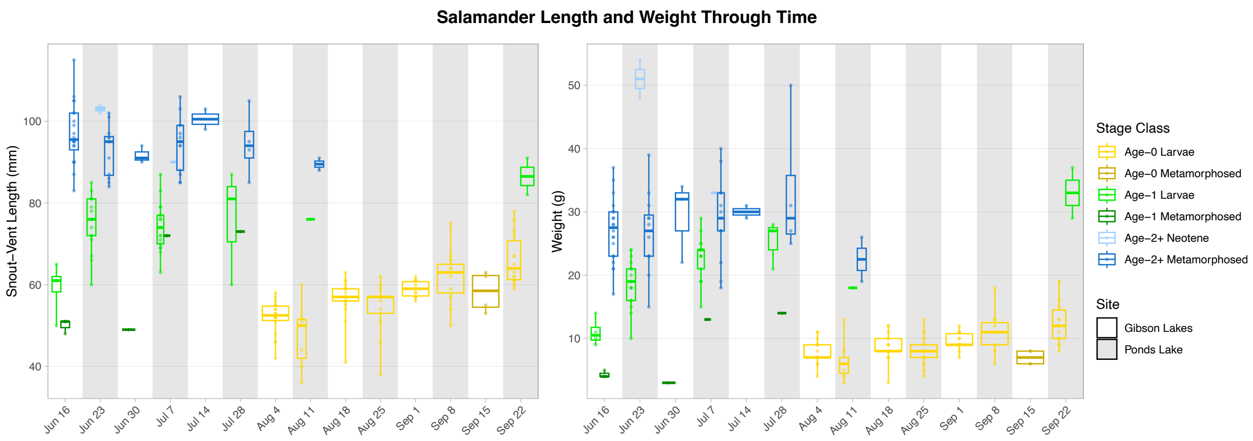

Figure S1. Salamander length and weight through time.

Library preparation

Library preparation for sequencing the 16S rRNA and ITS genetic barcoding regions for bacteria and fungi involved two-stage polymerase chain reaction (PCR) with MagBead cleanups in between. First stage PCR amplified the 16S and ITS genes, added unique dual index combinations to each sample, and added a portion of the Illumina Nextera adapter. Second stage PCR completed Illumina adapter addition. The 16S rRNA V4 region was amplified using the primers 515F (forward; Parada *et al.* 2016) and 806R (reverse; Caporaso 2011). The ITS1 region was amplified using the primers ITS1-F (forward; Gardes & Bruns 1993) and ITS2 (reverse; White *et al.* 1990). First stage PCR was a 15 µL reaction with 3 µL 5X Phusion HF Buffer, 0.45 µL 10M dNTPs, 0.3 µL Kapa HiFi HotStart DNA Polymerase, 3.25 µL high-performance liquid chromatography-grade water, 6 µL appropriate 0.25 µM paired primers, and 2 µL template DNA. First stage PCR was performed with the settings: 95 °C for 3 min, 15 cycles at 98 °C for 30 s, 15

cycles at 62 °C for 30 s, 15 cycles at 72 °C for 30 s, 72 °C for 5 min, and 4 °C for ever. Two first-stage PCR replicates were performed for each sample. Following first stage PCR, replicates were pooled together for each sample (*i.e.*, 15 µL of one replicate was transferred to the other) and purified using a modified manual AxyPrep MagBead PCR Clean-up protocol. For this protocol, the MagBead bottle was gently shaken to resuspend particles and equilibrated to room temperature. 24 µL of MagBead solution was added to each sample and pipette mixed 10 times followed by a 5-min incubation at room temperature. Samples were placed on a magnet plate and incubated for 5 min at room temperature or until the solutions were clear. The solutions were aspirated and discarded, and the beads were rinsed twice with 100 µL fresh 80% ethanol. The rinse involved adding ethanol, incubating for 30 s at room temperature, and aspirating and discarding the ethanol. Samples were re-aspirated to assure maximum ethanol removal and air dried for 7 min. Samples were removed from the magnet plate, 40 µL TE buffer was added to each sample and pipette mixed 10 times, and samples were incubated for 2 min at room temperature. Samples were placed back on the magnet plate for 5 min or until the solutions were clear, and 10 µL of solution was transferred for use as template DNA in the second stage PCR reaction.

Second stage PCR was a 15 µL reaction with 3 µL 5X Phusion HF Buffer, 0.45 µL 10M dNTPs, 0.3 µL Kapa HiFi HotStart DNA Polymerase, 0.5 µL 10 µM forward and reverse flow cell primers, 0.75 µL high-performance liquid chromatography-grade water, and 10 µL template DNA. Second stage PCR was performed with the settings: 95 °C for 3 min, 19 cycles at 98 °C for 30 s, 19 cycles at 55 °C for 30 s, 19 cycles at 72 °C for 30 s, 72 °C for 5 min, and 4 °C for ever. Following second stage PCR, 15 µL water was added to each sample. Samples were then purified using the same modified AxyPrep MagBead PCR Clean-up protocol as used before, except for 40 µL of solution (instead of 10 µL) was transferred during the final step to serve as the cleaned amplicon product.

Amplicon product concentrations were measured via absorption with Synergy HTX Take 3 Trio. Since amplicon product concentrations were of relatively similar concentrations (within an order of magnitude), 2 µL of each sample was combined across 8 tubes. The 8 tubes were vortexed and spun before 50 µL of each was combined into a final pooled tube.

DADA2 bioinformatics pipeline

Read pairs were quality filtered using DADA2's (version 3.10; Callahan *et al.* 2016) filterAndTrim function (truncating reads at the first instance of a quality score ≤ 2, discarding reads with more than 2 expected errors after truncation, discarding reads of less than 10 bp after truncation, and discarding reads which match against the phiX genome). DADA2's error-rate learning (learnErrors) and application of the core sample inference algorithm (dada) were performed using default settings. For the MiSeq 16S dataset, read pairs were merged using DADA2's mergePairs function (minimum overlap of 12 bp and allowing for no mismatches in the overlap region). For the MiSeq ITS dataset, which has variable length amplicon sequences whose read pairs may not overlap, mergePairs was applied as above but with rejects returned (returnRejects = TRUE). For unsuccessfully merged MiSeq ITS pairs, read pairs were concatenated (mergePairs with justConcatenate = TRUE) with a 10-N spacer between them if the overlap region was less than 12 bp (the DADA2 pipeline is made to work with these 10-N spacers), and unsuccessfully merged pairs with mismatches in an overlap region of at least 12 bp

were discarded (following DADA2's default behavior, we consider 12 bp to be a true overlap). Concatenated MiSeq ITS read pairs which were of forward or reverse reads which were previously truncated were discarded (to ensure unique non-overlapping sequences do not appear different because of differing lengths). To remove potential partial overlap between the remaining concatenated MiSeq ITS read pairs, the maximum possible length of the overlap region (the sum of the number of matches, number of mismatches, and number of indels in the overlap region from the mergePairs output) for each of these read pairs was trimmed from the reverse read segment on the end which could potentially overlap with the forward read. MiSeq sequences which were the synthgenes, coligos, or had lengths between or equal to 250 and 256 bp for 16S and greater than 125 bp for ITS were kept. Chimeric reads were removed for each of the MiSeq 16S and ITS datasets using DADA2's removeBimeraDenovo function with default settings, and synthgene and coligo sequences were subsequently removed. A naïve Bayesian classifier (Wang *et al.* 2007) was used to classify each of the MiSeq 16S and ITS datasets with DADA2's assignTaxonomy function using Silva (formatted by DADA2 with down to genus-level taxonomies; version 138; Quast *et al.* 2012) and UNITE (general dynamic FASTA release for fungi; version 8.2; Nilsson *et al.* 2019) reference libraries with default settings. For the MiSeq 16S dataset, species-level assignments were made through exact matching against a DADA2-maintained species-level Silva database using DADA2's addSpecies function with default settings. The MiSeq 16S dataset was subsetted to sequences which were classified as bacteria, and the MiSeq ITS dataset was subsetted to sequences which were classified as fungi.

Creating study-specific reference libraries

To create study-specific 16S and ITS reference libraries for classifying the NextSeq sequences, forward and reverse reads were created from the classified MiSeq 16S and ITS sequences which were the same lengths of the forward and reverse NextSeq 16S and ITS reads. The Biostrings R package (version 2.54.0; Pagès *et al.* 2019) was used to generate reverse complements of the MiSeq sequences during the creation of reverse reads. For the MiSeq 16S data, most truncated reference sequences were still unique after trimming reads to NextSeq lengths, with 104 out of 16,845 reference sequences becoming duplicates of each other. In R, these 104 non-unique truncated reference sequences were collapsed into 52 unique sequences and assigned a consensus taxonomy and consensus MiSeq-length sequence (for later use in alignment and prediction of *Bd*-inhibitory function). The consensus taxonomy was the most precise agreed upon taxonomy of the duplicated reference sequences. The consensus MiSeq-length sequences were created by replacing inconsistencies in the full-length reference sequences with the most parsimonious ambiguous nucleotide (all differences were substitutions). For the MiSeq ITS data, the same process was applied for creating consensus taxonomies for 87 non-unique truncated reference sequences out of 3,954 total. Since a reference taxon can occur multiple times in the reference libraries (being associated with multiple amplicon sequence variants [ASVs]), reference taxon names were appended with integers so that all reference taxa names were unique. Forward and reverse reference reads were also created for the 16S and ITS coligos and sythgenes, which were added to their respective study-specific reference libraries.

Mock community visualization

The proportional abundances (excluding the synthgenes from the calculations and after summing read counts across PCR replicates) of taxa detected in the mock community samples were visualized (Figure S2). The actual DNA composition of the mock communities were 12% *Listeria monocytogenes* (bacterium), 12% *Pseudomonas aeruginosa* (bacterium), 12% *Bacillus subtilis* (bacterium), 12% *Escherichia coli* (bacterium), 12% *Salmonella enterica* (bacterium), 12% *Lactobacillus fermentum* (bacterium), 12% *Enterococcus faecalis* (bacterium), 12% *Staphylococcus aureus* (bacterium), 2% *Saccharomyces cerevisiae* (fungus), and 2% *Cryptococcus neoformans* (fungus). Given this composition, we expected eight bacterial taxa in the 16S mock community data with uniform proportional abundances, and we expected two fungal taxa in the ITS mock community data also with uniform proportional abundances. We observed some amplification bias in the 16S data, and two 16S taxa (*Bacillus* and *Salmonella*) were split into two ASVs each (with the degree of the split being more pronounced for *Bacillus*). In our ITS mock community data, we observed much more extreme amplification bias, and one taxon (*Saccharomyces cerevisiae*) was split into three substantial ASVs. In an effort to mitigate the potential impact of fungal taxa being split into multiple ASVs, we merged fungal ASVs which were assigned the same taxonomy into the same taxa (and trailing integers in the ITS taxa names were removed).

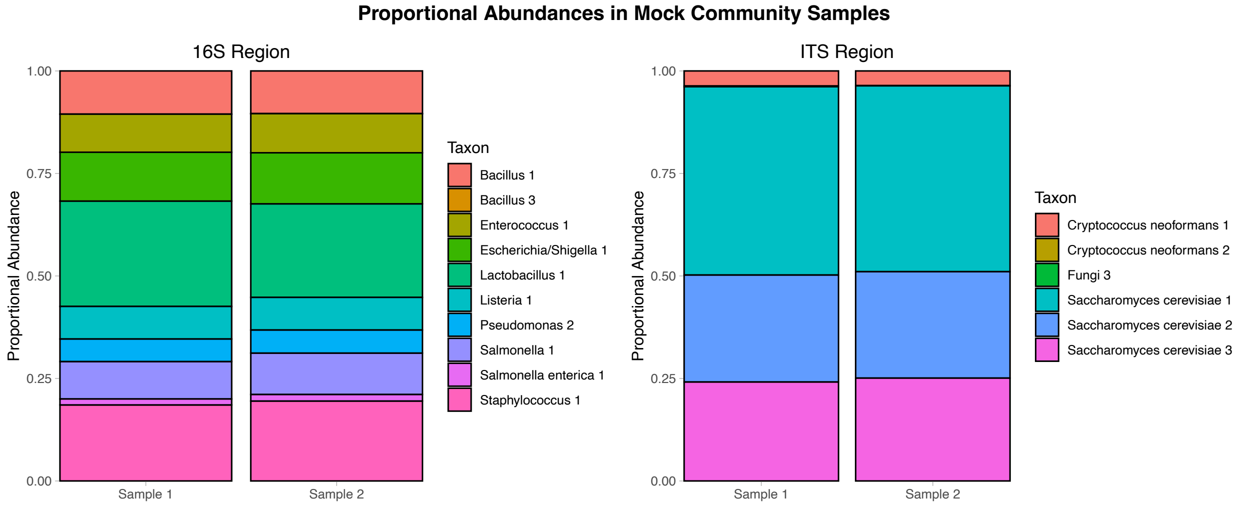

Figure S2. Observed composition of mock community samples.

Quality assessment of microbiome data

We performed principal component analyses (PCAs) on the proportional abundances of sample taxa (excluding the synthgenes from the calculations) across PCR replicates (Figure S3). The PCA plots on PCR replicate for both 16S and ITS samples suggest that taxa proportional abundances in both PCR replicates were similar, and read counts were summed across PCR replicates for each sample. We then performed PCAs on the proportional abundances of sample taxa (excluding the synthgenes from the calculations) across sample types (*i.e.*, salamander, wet swab, dry swab, blank, water, water control, substrate, and mock community samples) to check

for cross contamination between samples preceding library preparation. We performed separate 16S and ITS PCAs with random subsets of salamander samples (Figures S4 & S5) so the number of salamander, water, and substrate samples included in the PCAs was similar (51 to 67 of each type). There were 10 salamander samples which grouped closely with wet swab and dry swab negative controls in the 16S PCA plots, so these samples were removed from the 16S data for all subsequent analyses.

Following Harrison *et al.* (2021), we used synthgene read counts to calculate the amount of microbial DNA in each sample relative to the synthgene (i.e., microbial read count divided by sythgene read count) in each of the 16S and ITS datasets. There were 13 samples in which the synthgene was not detected (all were non-control water and substrate samples), and the synthgene read counts for these samples were set to one for the purpose of estimating the amount of microbial DNA (this only affects the following visualization). We visualized the amount of microbial DNA along with total read counts (excluding the synthgene) within each sample (Figure S6), and we observed higher amounts of microbial DNA within field samples (*i.e.*, non-control salamander, water, and substrate samples) compared to their associated negative controls.

While we observed 16S sequences classified as mitochondrial and chloroplast DNA, the composition of these sequences was minor (2.6% of salamander sample reads). We used online nucleotide BLAST (default settings; Zhang *et al.* 2000) to evaluate whether the mitochondrial and chloroplast sequences with the most salamander sample reads could be bacterial. A BLAST of the MiSeq-length Mitochondria 1 sequence yielded four top hits with > 97% identity. Two hits were for uncultured bacteria while the others were for *Aphanomyces* water molds. Similarly, a BLAST of the MiSeq-length Chloroplast 7 sequence yielded exact hits to uncultured bacteria in addition to plant chloroplast. Since amphibian skin microbiome studies do not typically remove mitochondrial or chloroplast sequences (*e.g.*, Longo *et al.* 2015; Kueneman *et al.* 2016b; Bletz *et al.* 2017a; Kruger 2020), and because it is possible that these sequences represent uncultured bacteria, we retain these sequences in our analyses.

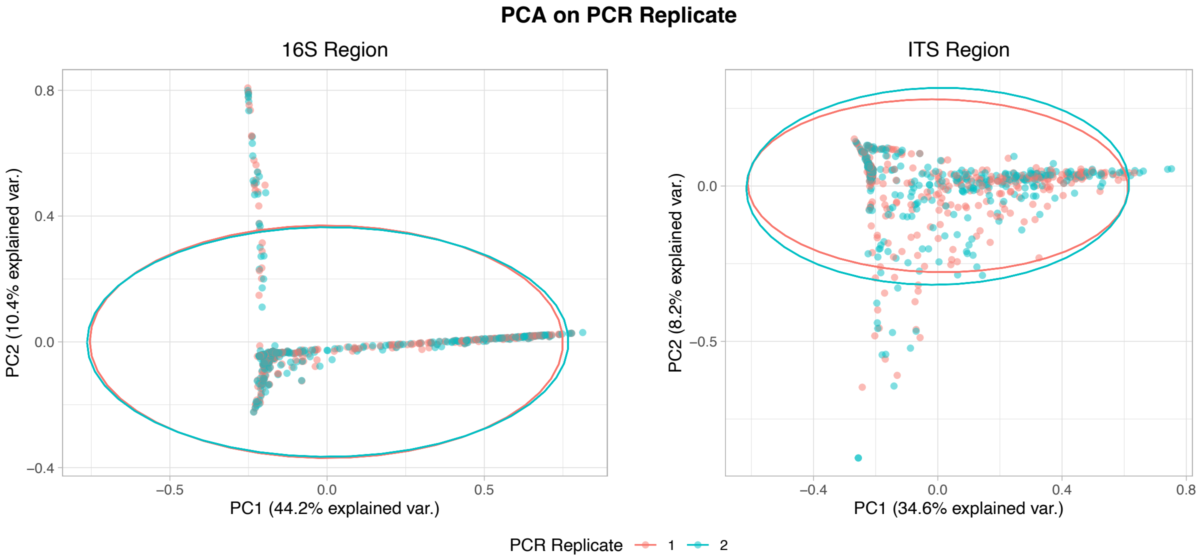

Figure S3. Principal component analyses (PCAs) on PCR replicate with 95% ellipses.

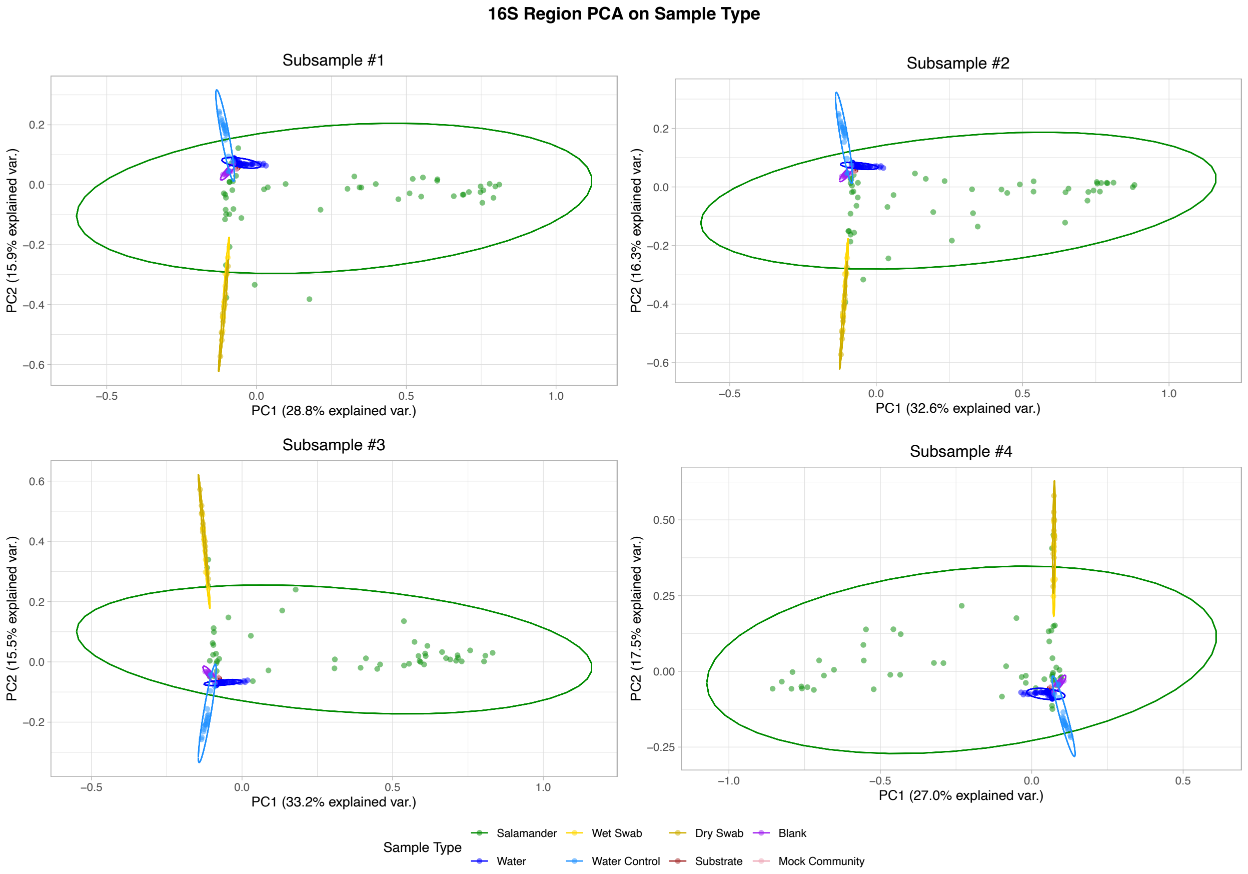

Figure S4. Principal component analyses (PCAs) on sample type with 95% ellipses for 16S data. The PCA in each plot contains a random subset of salamander samples.

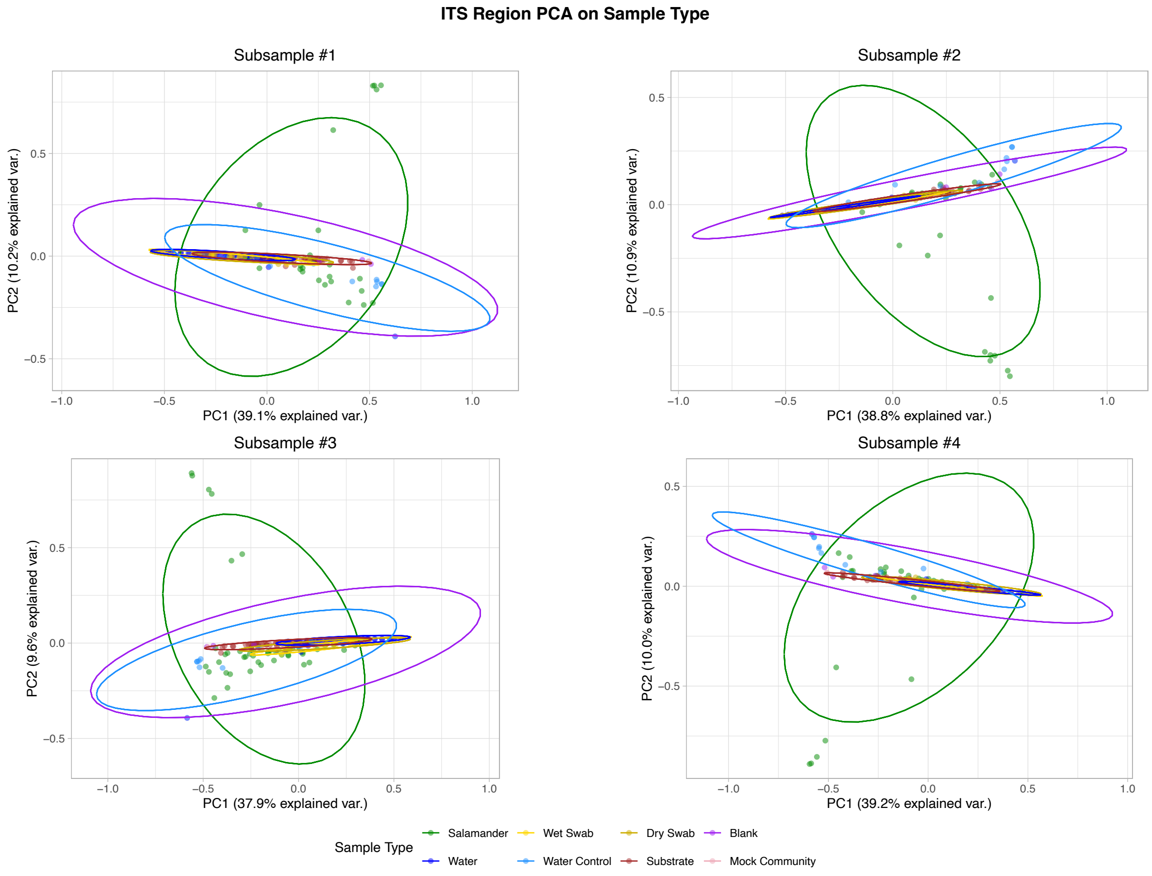

Figure S5. Principal component analyses (PCAs) on sample type with 95% ellipses for ITS data. The PCA in each plot contains a random subset of salamander samples.

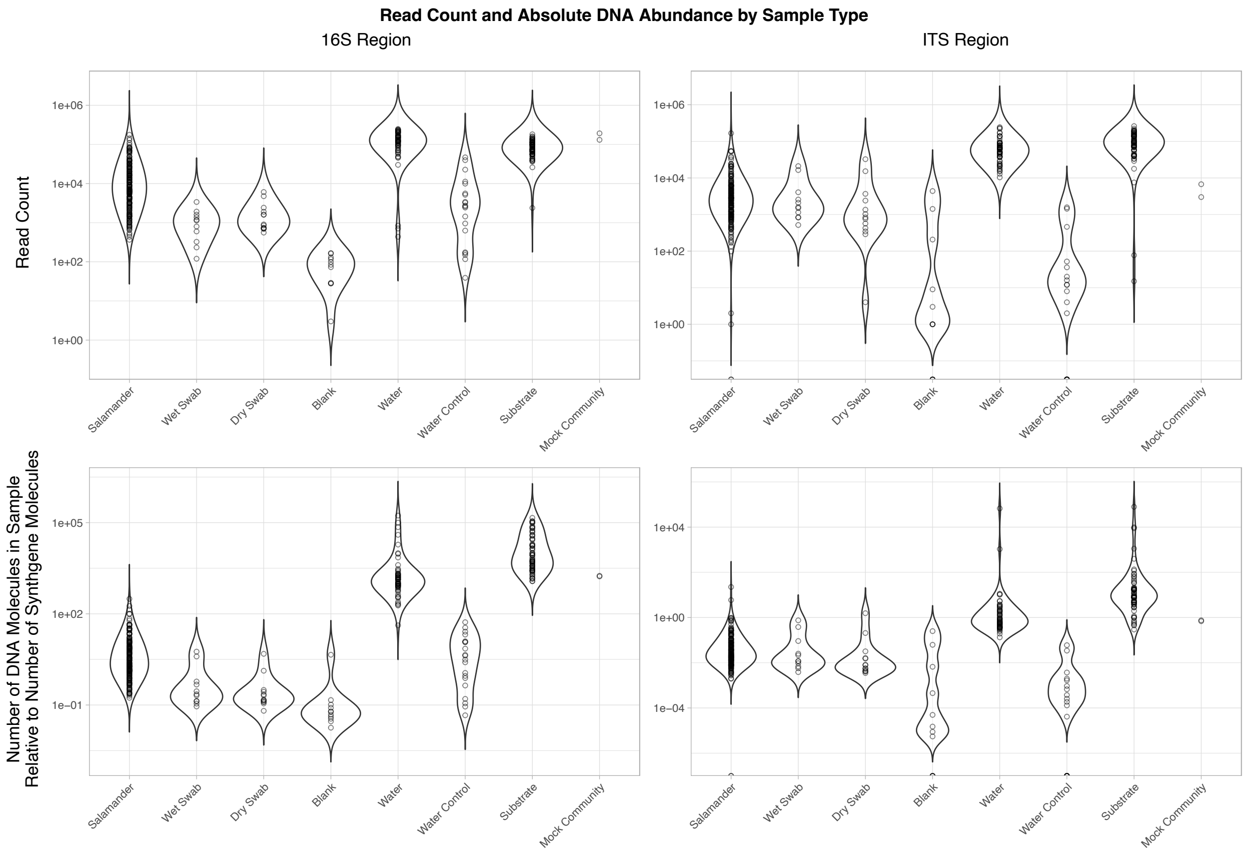

Figure S6. Sample read counts and amount of microbial DNA by sample type and barcode region. Note the $\log_{10}$ scale on the y-axis.

Predicting Bd-inhibitory function

The Woodhams *et al.* (2015) database of amphibian skin-associated microbiome *Batrachochytrium dendrobatidis* (*Bd*)-inhibitory bacterial isolates was subsetted to just bacteria (using the UCLUST taxonomies provided in the database), and taxa with an unknown *Bd*-inhibition status were removed. The remaining Woodhams *et al.* (2015) taxa that belonged to *Bd*-inhibition statuses that were not *Bd*-inhibitory were reclassified as non-inhibitory. Woodhams *et al.* (2015) sequences were trimmed to the 16S rRNA V4 region using our 16S amplification primers, and sequences without matches for both forward and reverse amplification primers were discarded. Trimmed Woodhams *et al.* (2015) sequences which were outside the range of 250 to 256 bp were discarded. This left 872 Woodhams *et al.* (2015) sequences available for alignment of the 1,944 sequences originally in the Woodhams *et al.* (2015) database.

We used Clustal Omega (version 1.2.4; Sievers *et al.* 2011) with default settings to align the MiSeq 16S sequences of taxa detected in our NextSeq 16S non-control samples (*i.e.*, salamander, water, and substrate samples) with the Woodhams *et al.* (2015) sequences, and we used FastTree 2 (version 2.1.11; Price *et al.* 2010) with a GTR+CAT model to create a phylogenetic tree. The tree was midpoint-rooted using midpoint.root in the phytools package (version 0.7.70; Revell 2012), and polytomies were randomly resolved into dichotomies (with

zero-length branches) using multi2di in the ape package (version 5.4.1; Paradis & Schliep 2019). Edges of zero length were set to 10^{-6} times the total tree length. An equal-rates transition matrix was estimated with fitMk (phytools package), and a stochastic character mapping model was fit with this transition matrix using make.simmap (phytools package) and mclapply (parallel package) to simulate 5,000 phylogenetic trees with mapped *Bd*-inhibition statuses. Our NextSeq 16S taxa received a prior probability of being *Bd*-inhibitory of 0.5, while the Woodhams *et al.* (2015) taxa received a zero (non-inhibitory) or a one (inhibitory). The posterior probabilities of our taxa being *Bd*-inhibitory were extracted from the fitted trees using describe.simmap in the phytools package.

Microbial composition modeling

Following Harrison *et al.* (2020), we added one to all read counts in the datasets (after the selection of the top 100 taxa and the creation of "other" categories) to avoid errors which occur when Dirichlet parameters in the model approach zero. Our Bayesian Dirichlet-multinomial regression model is adapted from the Bayesian Dirichlet regression model of Sennhenn-Reulen (2018), and our model takes the form:

Sample read counts c_{i_k} are distributed according to the Dirichlet-multinomial distribution with expected proportions p_{i_k} and precision parameter θ, which controls the degree of overdispersion relative to the multinomial distribution.

$$c_{i_1} \dots c_{i_K} \sim \text{Dirichlet-multinomial}\left(p_{i_1} \dots p_{i_K} * e^{\theta}, \sum_{k=1}^{K} c_{i_k}\right)$$

The softmax function normalizes linear predictor combinations η_{i_k} into proportions p_{i_k}.

$$p_{i_1} \dots p_{i_K} = \text{softmax}(\eta_{i_1} \dots \eta_{i_K})$$

$$\text{softmax}(\eta_{i_1} \dots \eta_{i_K}) = \frac{e^{\eta_{i_1}}}{\sum_{k=1}^{K} e^{\eta_{i_k}}} \cdots \frac{e^{\eta_{i_K}}}{\sum_{k=1}^{K} e^{\eta_{i_k}}}$$

$$\eta_{i_k} = \beta_{0_k} + B_{\text{Stratum}_k} * X_{\text{Stratum}_i}^{\top} + B_{\text{Non-stratum}_k} * X_{\text{Non-stratum}_i}^{\top}$$

$$B_{\text{Stratum}_k} = \left[\beta_{\text{Stratum}_{1_k}} \cdots \beta_{\text{Stratum}_{S_k}}\right]$$

$$B_{\text{Non-stratum}_k} = \left[\beta_{\text{Non-stratum}_{1_k}} \cdots \beta_{\text{Non-stratum}_{Q_k}}\right]$$

$$X_{\text{Stratum}_i} = \left[x_{\text{Stratum}_{1_i}} \cdots x_{\text{Stratum}_{S_i}}\right]$$

$$X_{\text{Non-stratum}_i} = \left[x_{\text{Non-stratum}_{1_i}} \cdots x_{\text{Non-stratum}_{Q_i}}\right]$$

The intercept terms β_0, stratum regression coefficients β_{Stratum_s}, and non-stratum regression coefficients $\beta_{\text{Non-stratum}_q}$ of the last taxon K are set to zero to allow for the model to be identifiable.

$$\beta_{0_K} = 0$$

$$\beta_{\text{Stratum}_{s_K}} = 0$$

$$\beta_{\text{Non-stratum}_{q_K}} = 0$$

A normal prior is used on precision θ.

$$\theta \sim \text{normal}(0, 1)$$

Except for the last taxon K, weakly informative priors are used on the intercept terms β_{0_k} and non-stratum regression coefficients $\beta_{\text{Non-stratum}_{q_k}}$.

$$\beta_{0_{k \neq K}} \sim \text{normal}(0, 1)$$

$$\beta_{\text{Non-stratum}_{q_{k \neq K}}} \sim \text{normal}(0, 1)$$

Stratum is treated as a hierarchical effect. For each taxon except the last taxon K, stratum regression coefficients $\beta_{\text{Stratum}_{s_k}}$ share a common variance term $\sigma^2{}_{\text{Stratum}_k}$, except for the last stratum regression coefficient $\beta_{\text{Stratum}_{S_k}}$, which is set to the negative sum of the preceding stratum regression coefficients $\beta_{\text{Stratum}_{s \neq S_k}}$. This applies sum-to-zero constraints to allow for identifiability.

$$\beta_{\text{Stratum}_{s \neq S_{k \neq K}}} \sim \text{normal}\left(0, \sigma^2{}_{\text{Stratum}_k}\right)$$

$$\sigma^2{}_{\text{Stratum}_k} \sim \text{inverse-gamma}(0.01, 0.01)$$

$$\beta_{\text{Stratum}_{S_{k \neq K}}} = -1 * \sum_{s=1}^{S-1} \beta_{\text{Stratum}_{s_k}}$$

Where c_{i_k} is the read count,
$i = 1 \ldots N$ is the sample number,
$k = 1 \ldots K$ is the taxon number,
$s = 1 \ldots S$ is the stratum number,
$x_{\text{Stratum}_{s_i}}$ is the stratum predictor value,
$q = 1 \ldots Q$ is the non-stratum predictor number,
and $x_{\text{Non-stratum}_{q_i}}$ is the non-stratum predictor value.

For the salamander samples, stratum, site, and life stage were treated as categorical predictors and were coded as binary dummy variables. Stratum was coded as nine dummy variables to represent the four strata in Gibson Lakes and the five strata in Ponds Lake. These categorical predictors were mean-centered to aid in model convergence. Since mean centering does not affect the range in the dummy predictor values (*i.e.*, the range remains one), the interpretation of modeled effects is not impacted (Gelman 2008) and the sum-to-zero constraints on hierarchical stratum effects remain unaffected. Age and time were treated as continuous predictors. These predictors were mean-centered and scaled by one standard deviation to aid in model convergence and to reduce predictor collinearity. Age was coded from zero (age-0) to two (age-2+). Time was coded as the number of weeks since June 9th, 2018 and was also given a quadratic term. After centering, scaling, or both, interactions between all of the non-stratum predictors were created such that the non-stratum predictor matrix included four-way interactions between age, life stage, site, and a second-degree polynomial for week, all lower-level interactions, and the individual predictors.

Backwards variable selection by WAIC operated by fitting an initial model with all 24 predictors and calculating the model's WAIC (using method 2 in Gelman *et al.* 2014, as

recommended). The ddirmnom function in the extraDistr package (version 1.8.11; Wolodzko 2019) was used to compute log probability masses during WAIC calculations. A set of 23 models were subsequently fit by removing a single predictor from the initial model. WAICs were calculated for these models and compared to the initial model's WAIC. If the lowest WAIC in the new model set was less than or equal to the initial model's WAIC, then the model in the model set with the lowest WAIC was considered to be the new benchmark for best predictive accuracy. The process repeated by fitting another set of models with a single predictor removed from the benchmark model and comparing WAICs. When removing additional predictors failed to reduce or match WAIC, or after an intercept-only model was fit and no more predictors could be removed, then the variable selection process was finished and the model with the lowest WAIC of all the models fit was considered to have the best predictive accuracy. We considered the model with the best predictive accuracy to be the best-fit model. During backwards variable selection, the stratum dummy variables were considered to be a single predictor and were removed as a group.

The same Bayesian Dirichlet-multinomial regression model and backwards variable selection approach were used to fit models to water and substrate read counts. Models for water samples were treated separately from models for substrate samples. For both water and substrate models, the non-stratum predictor matrix included site, a second-degree polynomial for week, and all interactions between these predictors. Centering and scaling of categorical and continuous predictors was performed in the same way as for the salamander models.

These same steps were performed for data from each of the 16S and ITS barcode regions, and for salamander samples, additional models were fit which substituted spatiotemporal covariates (*i.e.*, stratum, site, and time) with water quality predictors. The predictor matrix for the water quality models included a five-way interaction between age, life stage, temperature (°C), pH, and dissolved oxygen (ppm), all lower-level interactions, and the individual predictors. Conductivity (μS) was excluded from the analysis due to being highly colinear with pH (Pearson correlation coefficient of 0.817). We missed dissolved oxygen measurements (both ppm and percent) on August 11th at Ponds Lake due to a faulty dissolved oxygen meter. For this analysis, we estimated dissolved oxygen (ppm) on this day by taking the average of the dissolved oxygen (ppm) measurements at each stratum for the weeks sampled immediately before and after at Ponds Lake. Quadratic terms were not introduced for the water quality models both because the Bayesian Dirichlet-multinomial regression model is already quite flexible and because including such terms would have greatly increased the run-time required to perform backwards variable selection. Water quality predictors were mean-centered and scaled by one standard deviation prior to the creation of interaction terms. For the water quality models, parts of the Bayesian Dirichlet-multinomial regression model associated with stratum were removed, and the predictor matrix was treated as the non-stratum predictor matrix.

The Bayesian Dirichlet-multinomial regression models were fit in Stan (version 2.21.0; Carpenter *et al.* 2017) using the rstan R interface (version 2.21.2; Stan Development Team 2020) for Stan's Hamiltonian Monte Carlo (HMC) No-U-Turn sampler. Each model had four HMC chains run in parallel on four cores, and each chain had 500 warmup iterations and 500 sampling iterations without thinning. The average probability of accepting a posterior draw was set to 95%, and maximum tree depth was set to 20. When models were fit which excluded stratum predictors, which excluded all non-stratum predictors, or both, appropriate Stan models were used in which

model parts associated with these excluded predictors were removed. The number of $\hat{R}$ convergence diagnostics in each model which were > 1.05 (disregarding the $\hat{R}$s of fixed parameters set to zero) were monitored, and for the final models selected by backwards variable selection, trace plots of the posteriors were used to assess convergence for parameters with $\hat{R}$s > 1.05. The vast majority of all models fit during the backwards variable selection process had zero $\hat{R}$s > 1.05, and many of these models had thousands of parameters each. The selected spatiotemporal models for the water 16S region, salamander ITS region, and substrate ITS region each had at least one parameter with $\hat{R}$ > 1.05. The selected water 16S region model had 19 parameters with $\hat{R}$s > 1.05, and six parameters with $\hat{R}$s > 1.1 (all < 1.11). Examining the trace plots of parameters with $\hat{R}$s > 1.05 revealed that these parameters had some spurious draws which quickly returned towards the other chains, and otherwise convergence appeared fine. The selected salamander ITS region model had 46 parameters with $\hat{R}$s > 1.05, and two parameters with $\hat{R}$s > 1.1 (1.107 and 1.127). The trace plots of parameters with $\hat{R}$s > 1.05 appeared fine. The selected substrate ITS region model had 3 parameters with $\hat{R}$s > 1.05 (all < 1.08), and the trace plots of these parameters appeared fine. The selected water quality salamander models for both barcode regions had no parameters with $\hat{R}$s > 1.05. The selected *Bd*-inhibition models also had no parameters with $\hat{R}$s > 1.05.

Microbial composition predictions

Due to the softmax function and the compositional nature of the Bayesian Dirichlet-multinomial regression model, the effect of regression coefficients on proportional abundances is difficult to interpret. While taxa with the same regression coefficients within a predictor experience the same percent change in proportional abundances with changes in that predictor, resulting in the same shape of relationship, additional interpretation is limited since the effect of one regression coefficient on proportional abundances is dependent on the values of all other regression coefficients in the model. The modeled proportional abundances do not change linearly with changes in predictor values, and changes in predictor values elsewhere in the model can change whether proportional abundances are increasing or decreasing at a given value of another predictor. Furthermore, the regression coefficients do not represent changes in underlying absolute abundances since a value of zero represents the regression coefficient values of the reference taxon, the selection of which is arbitrary.

Instead of attempting a direct interpretation of the Bayesian Dirichlet-multinomial regression coefficients, we opted for a graphical interpretation of the best-fit models using predicted proportional abundances. For the spatiotemporal salamander, water, and substrate models, we generated posterior proportional abundance predictions for each combination of non-stratum predictors observed in the datasets. Posterior predictions were also performed for best-fitting salamander, water, and substrate *Bd*-inhibition models. For water, proportional abundance predictions were also made for July 14th at Gibson Lakes, a sampling event for which water samples had to be discarded. Non-stratum predictor matrices were prepared as in the compositional modeling section with the following differences. Mean centering and standard deviation scaling were performed using the same mean and standard deviation values used to center and scale the models' original predictors, and non-stratum predictors were subsetted to just those included in the best-fit models. If stratum was included as a predictor in the best-fit model,

then a stratum predictor matrix was prepared as described above, and all dummy variables were set to zero prior to centering with the same mean values used to center the model's original stratum predictors. Due to the sum-to-zero constraint on all stratum regression coefficients within a taxon, the average effect of stratum for a taxon is zero, and setting stratum predictor values to either all zero or all one prior to centering results in predictions for the average stratum. This is analogous to leaving out random effects from frequentist models to achieve group-level predictions. These predictions represent the posterior distributions of taxa proportional abundances.

Relationship between the relative abundances of Bd-inhibitory bacteria and Bd

Our Bayesian beta-binomial regression model between the relative abundances of *Bd*-inhibitory bacteria and *Bd* on the skin of metamorphosed salamanders takes the form:

Bd read counts c_{Bd_i} are distributed according to the beta-binomial distribution with total fungal read counts f_i and shape parameters α_{Bd_i} and β_{Bd_i}. A precision parameter ϕ controls the degree of overdispersion relative to the binomial distribution.

$$c_{\mathrm{Bd}_i} \sim \text{beta-binomial}\left(f_i, \alpha_{\mathrm{Bd}_i}, \beta_{\mathrm{Bd}_i}\right)$$
$$\alpha_{\mathrm{Bd}_i} = p_{\mathrm{Bd}_i} * \phi$$
$$\beta_{\mathrm{Bd}_i} = \left(1 - p_{\mathrm{Bd}_i}\right) * \phi$$

A logit link is used for the proportional abundance of *Bd* in fungal communities p_{Bd_i}, and the logit linearizes the proportional abundance of *Bd*-inhibitory bacteria in bacterial communities p_{Inhib_i} for use as a predictor.

$$\mathrm{logit}\left(p_{\mathrm{Bd}_i}\right) = \beta_0 + \beta_1 * \mathrm{logit}\left(p_{\mathrm{Inhib}_i}\right)$$

For each sample, the proportional abundance of *Bd*-inhibitory bacteria in bacterial communities p_{Inhib_i} is estimated within the model using the beta-binomial distribution with the read count of *Bd*-inhibitory bacteria c_{Inhib_i}, the total bacterial read count b_i, and shape parameters $\alpha_{\mathrm{Inhib}_i}$ and β_{Inhib_i}. The precision parameter ϕ again controls the degree of overdispersion relative to the binomial distribution.

$$c_{\mathrm{Inhib}_i} \sim \text{beta-binomial}\left(b_i, \alpha_{\mathrm{Inhib}_i}, \beta_{\mathrm{Inhib}_i}\right)$$
$$\alpha_{\mathrm{Inhib}_i} = p_{\mathrm{Inhib}_i} * \phi$$
$$\beta_{\mathrm{Inhib}_i} = \left(1 - p_{\mathrm{Inhib}_i}\right) * \phi$$

For each sample, a uniform prior between zero and one is used on the proportional abundance of *Bd*-inhibitory bacteria in bacterial communities p_{Inhib_i}.

$$p_{\mathrm{Inhib}_i} \sim \mathrm{beta}(1,1)$$

Weakly informative normal priors are used on the intercept term β_0 and regression coefficient β_1.

$$\beta_0 \sim \mathrm{normal}(0, 10^6)$$
$$\beta_1 \sim \mathrm{normal}(0, 10^6)$$

A weakly informative gamma prior is used on precision ϕ.

$$\phi \sim \mathrm{gamma}(0.01,0.01)$$

Where c_{Bd_i} is the read count of *Bd*,
c_{Inhib_i} is the read count of *Bd*-inhibitory bacteria,
f_i is the total fungal read count,
b_i is the total bacterial read count,
and $i = 1 \dots N$ is the sample number.

The Bayesian beta-binomial regression model was fit in JAGS (version 4.3.0; Plummer 2003) using the rjags R interface (version 4.10; Plummer 2019) with three Markov chain Monte Carlo (MCMC) chains, 20,000 adaptation iterations, 20,000 warmup iterations, 100,000 sampling iterations, and no thinning. Gelman-Rubin convergence diagnostics and trace plots of the posteriors were used to assess model convergence.

Salamander length-weight regression

We performed a regression between salamander length and weight to evaluate whether length squared could be used as a surrogate for swabbed area in absolute abundance estimation using the sythgenes. Snout-vent length (SVL) can be related to weight via a power function (Hile 1936) which takes the form:

$$\text{Weight (g)} = a * \text{SVL (mm)}^b$$

Where *a* and *b* are constants. If *b* = 3, then the organism exhibits isometric growth where the body grows proportionally, and the organism exhibits allometric growth otherwise. The relationship can be linearized by taking the logarithm of both sides:

$$\log(\text{Weight (g)}) = \log(a) + b * \log(\text{SVL (mm)})$$

Fitting the linearized model between salamander SVL and weight while including an interaction term between SVL and site yielded an insignificant interaction p-value of 0.166. Since there was insufficient evidence to suggest a unique length-weight relationship for each site, we fit a common length-weight relationship for both sites by excluding the interaction term, and we performed a t-test to determine whether *b* was significantly different than 3 using the hoCoef function in the FSA package (version 0.8.30; Ogle *et al.* 2020). A t-test p-value of < 0.001 suggested that *b* differed significantly from 3 and that salamander growth was allometric. Because salamander growth was allometric, SVL squared could not be used as a surrogate for swabbed area.

Results

Water quality

The field season finished on September 29th when Gibson Lakes became too cold to safely sample salamanders. Water quality and environmental microbiomes were still taken at Gibson Lakes on September 29th despite not catching any salamanders. The water level at Gibson Lakes on September 29th was too low to measure relative lake elevation.

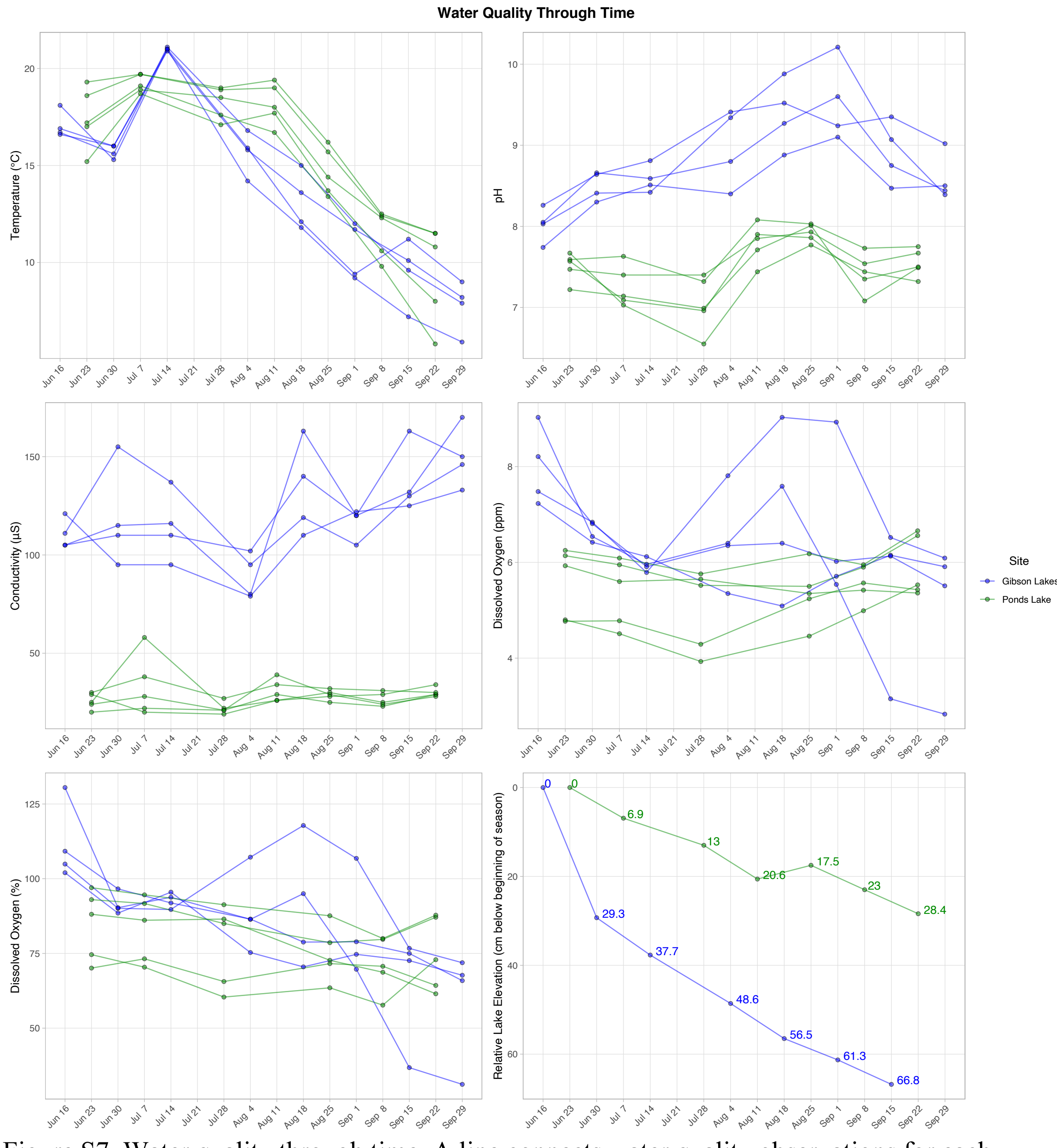

Figure S7. Water quality through time. A line connects water quality observations for each stratum.

Table S1. Parameter estimates from linear mixed-effects models of water quality. Site represents the effect of Ponds Lake relative to Gibson Lakes.

Water Quality Parameter	Model Parameter	Regression Coefficient	Standard Error	Degrees of Freedom	t-statistic	p-value
Temperature	Intercept	19.955	0.836	40.713	23.864	< 0.001*
	Site	1.536	1.216	47.295	1.264	0.212
	Week	-0.713	0.080	55.696	-8.918	< 0.001*
	Interaction	0.030	0.119	55.696	0.253	0.801
pH	Intercept	8.356	0.160	32.273	52.189	< 0.001*
	Site	-1.048	0.231	38.382	-4.544	< 0.001*
	Week	0.053	0.014	56.187	3.713	< 0.001*
	Interaction	-0.031	0.021	56.187	-1.472	0.147
Conductivity	Intercept	102.268	5.373	49.939	19.035	< 0.001*
	Site	-74.822	7.861	55.163	-9.519	< 0.001*
	Week	2.197	0.535	56.687	4.107	< 0.001*
	Interaction	-2.120	0.793	56.687	-2.673	0.010*
Dissolved Oxygen (ppm)	Intercept	7.487	0.408	18.946	18.350	< 0.001*
	Site	-2.233	0.580	22.837	-3.853	< 0.001*
	Week	-0.126	0.031	51.074	-4.031	< 0.001*
	Interaction	0.151	0.046	51.074	3.254	0.002*
Dissolved Oxygen (%)	Intercept	109.717	5.505	15.299	19.930	< 0.001*
	Site	-23.884	7.749	18.119	-3.082	0.006*
	Week	-2.835	0.384	50.892	-7.380	< 0.001*
	Interaction	1.901	0.570	50.892	3.336	0.002*

* p-value less than 0.05.

Predictions of Bd-inhibitory function

Table S2. Predicted *Batrachochytrium dendrobatidis* (*Bd*)-inhibition statuses of bacterial taxa. Bacterial taxa not listed here have an uncertain *Bd*-inhibition status.

Predicted *Bd*-Inhibition Status	Taxon	Probability of *Bd*-Inhibition
Inhibitory	*Aeromonas* 1	99.9%
	Allorhizobium-Neorhizobium-Pararhizobium-Rhizobium 11	99.9%
	Bacillus 3	99.9%
	Brevibacterium 1	99.9%
	Comamonadaceae 5	99.8%
	Duganella 1	> 99.9%
	Duganella zoogloeoides 1	99.9%
	Flavobacterium 23	99.9%
	Flavobacterium 24	99.8%
	Hafnia-Obesumbacterium 1	99.8%
	Janthinobacterium 2	99.9%
	Massilia 1	99.7%
	Micrococcus 1	99.9%
	Paenibacillus 3	99.8%
	Paucibacter 2	99.9%
	Pseudarthrobacter 1	99.9%
	Pseudomonas 1	99.9%
	Pseudomonas 2	99.9%
	Pseudomonas 3	99.9%
	Pseudomonas 5	99.9%
	Salmonella 1	99.9%
	Staphylococcus 2	99.9%
	Stenotrophomonas 1	99.9%
	Streptomyces 5	99.8%
	Variovorax 1	99.8%
Non-Inhibitory	*Acinetobacter* 6	< 0.1%
	Acinetobacter 7	0.1%
	Chryseobacterium 1	0.1%
	Chryseobacterium 3	0.1%
	Enterobacterales 1	0.1%
	Flavobacterium 2	0.2%
	Streptomyces 3	0.1%
	Yersinia 1	0.2%

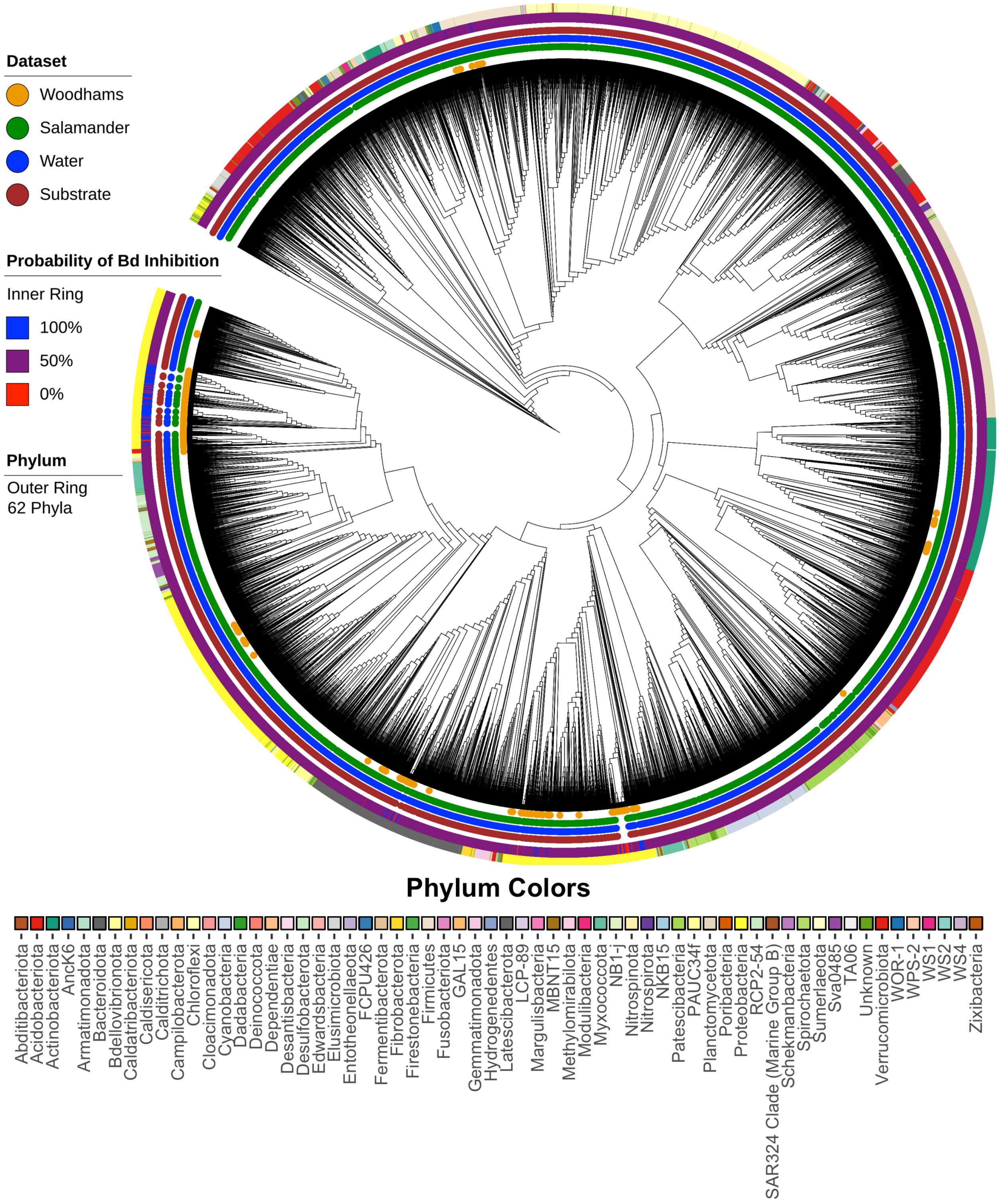

Figure S8. A phylogenetic tree of bacterial taxa observed in field samples and the Woodhams *et al.* (2015) database. Displayed with the tree is which sample type or dataset each taxon was observed in, each taxon's posterior probability of being *Batrachochytrium dendrobatidis* (*Bd*)-

inhibitory, and each taxon's phylum. Phyla for the Woodhams *et al.* (2015) taxa are from the UCLUST taxonomies provided in the database (Bacteroidetes and Actinobacteria were changed to their synonyms Bacteroidota and Actinobacteriota to match the taxonomy of our Silva reference database). As there are many phyla, some have similar colors, and one phylum color represents taxa whose phyla are unknown.

Top ten Bayesian Dirichlet-multinomial regression models

Table S3. The ten Bayesian Dirichlet-multinomial regression models from the backwards variable selection process with the lowest widely applicable information criterion (WAIC) values for bacterial communities on salamander skin. Ten models are displayed for each predictor set. DO is dissolved oxygen.

Predictor Set	Predictors	ΔWAIC
Spatiotemporal	Stratum + Age + Life Stage + Site + Age:Life Stage + Age:Site + Life Stage:Site + Life Stage:Week + Site:Week + Site:Week^2 + Age:Life Stage:Site + Age:Life Stage:Week + Age:Site:Week + Life Stage:Site:Week^2 + Age:Life Stage:Site:Week^2	0
	Stratum + Life Stage + Site + Age:Life Stage + Age:Site + Life Stage:Site + Life Stage:Week + Site:Week + Site:Week^2 + Age:Life Stage:Site + Age:Life Stage:Week + Age:Site:Week + Life Stage:Site:Week + Life Stage:Site:Week^2 + Age:Life Stage:Site:Week^2	12.1
	Stratum + Age + Life Stage + Age:Life Stage + Age:Site + Life Stage:Site + Life Stage:Week + Site:Week + Site:Week^2 + Age:Life Stage:Site + Age:Life Stage:Week + Age:Site:Week + Life Stage:Site:Week^2 + Age:Life Stage:Site:Week^2	13.5
	Stratum + Age + Life Stage + Site + Age:Life Stage + Age:Site + Life Stage:Week + Site:Week + Site:Week^2 + Age:Life Stage:Site + Age:Life Stage:Week + Age:Site:Week + Life Stage:Site:Week^2 + Age:Life Stage:Site:Week^2	14.8
	Stratum + Age + Life Stage + Site + Age:Life Stage + Life Stage:Site + Life Stage:Week + Site:Week + Site:Week^2 + Age:Life Stage:Site + Age:Life Stage:Week + Age:Site:Week + Life Stage:Site:Week^2 + Age:Life Stage:Site:Week^2	22.1
	Stratum + Life Stage + Site + Age:Life Stage + Age:Site + Life Stage:Site + Life Stage:Week + Site:Week + Site:Week^2 + Age:Life Stage:Site + Age:Life Stage:Week + Age:Site:Week + Life Stage:Site:Week^2 + Age:Life Stage:Site:Week^2	27.6
	Stratum + Age + Life Stage + Site + Age:Site + Life Stage:Site + Life Stage:Week + Site:Week + Site:Week^2 + Age:Life Stage:Site + Age:Life Stage:Week + Age:Site:Week + Life Stage:Site:Week^2 + Age:Life Stage:Site:Week^2	28.2
	Stratum + Age + Site + Age:Life Stage + Age:Site + Life Stage:Site + Life Stage:Week + Site:Week + Site:Week^2 + Age:Life Stage:Site + Age:Life Stage:Week + Age:Site:Week + Life Stage:Site:Week^2 + Age:Life Stage:Site:Week^2	32.1
	Stratum + Age + Life Stage + Site + Age:Life Stage + Age:Site + Life Stage:Site + Life Stage:Week + Site:Week + Site:Week^2 + Age:Life Stage:Site + Age:Life Stage:Week + Age:Site:Week + Life Stage:Site:Week + Life Stage:Site:Week^2 + Age:Life Stage:Site:Week^2	32.8
	Stratum + Age + Life Stage + Site + Age:Life Stage + Age:Site + Life Stage:Site + Life Stage:Week + Site:Week^2 + Age:Life Stage:Site + Age:Life Stage:Week + Age:Site:Week + Life Stage:Site:Week + Life Stage:Site:Week^2 + Age:Life Stage:Site:Week^2	32.9
Water Quality	Age + Life Stage + Life Stage:Temperature + Life Stage:pH + Temperature:pH + Life Stage:DO (ppm) + Age:Life Stage:pH + Age:pH:DO (ppm) + Age:Life Stage:Temperature:DO (ppm) + Life Stage:Temperature:pH:DO (ppm)	0
	Age + Life Stage + Life Stage:Temperature + Life Stage:pH + Temperature:pH + Life Stage:DO (ppm) + Age:Life Stage:pH + Age:pH:DO (ppm) + Life Stage:Temperature:pH:DO (ppm)	8.6
	Age + Life Stage + Life Stage:Temperature + Life Stage:pH + Temperature:pH + Life Stage:DO (ppm) + Age:Life Stage:pH + Age:Life Stage:Temperature:DO (ppm) + Life Stage:Temperature:pH:DO (ppm)	17.4
	Age + Life Stage + Life Stage:Temperature + Life Stage:pH + Temperature:pH + Life Stage:DO (ppm) + Age:Life Stage:pH + Age:Life Stage:Temperature:DO (ppm) + Life Stage:Temperature:pH:DO (ppm) + Age:Life Stage:Temperature:pH:DO (ppm)	35.4
	Age + Life Stage + Life Stage:Temperature + Life Stage:pH + Temperature:pH + Life Stage:DO (ppm) + Age:Life Stage:pH + Age:pH:DO (ppm) + Age:Life Stage:Temperature:DO (ppm) + Life Stage:Temperature:pH:DO (ppm) + Age:Life Stage:Temperature:pH:DO (ppm)	48.3
	Age + Life Stage + Life Stage:Temperature + Life Stage:pH + Temperature:pH + Life Stage:DO (ppm) + pH:DO (ppm) + Age:Life Stage:pH + Age:pH:DO (ppm) + Age:Life Stage:Temperature:DO (ppm) + Age:Life Stage:Temperature:pH:DO (ppm)	49.3
	Age + Life Stage + Life Stage:Temperature + Life Stage:pH + Temperature:pH + Life Stage:DO (ppm) + Age:Life Stage:pH + Age:pH:DO (ppm) + Life Stage:Temperature:pH:DO (ppm) + Age:Life Stage:Temperature:pH:DO (ppm)	52.6
	Age + Life Stage + Life Stage:Temperature + Life Stage:pH + Temperature:pH + Life Stage:DO (ppm) + pH:DO (ppm) + Age:Life Stage:pH + Age:pH:DO (ppm) + Age:Life Stage:Temperature:DO (ppm) + Life Stage:Temperature:pH:DO (ppm) + Age:Life Stage:Temperature:pH:DO (ppm)	57.3
	Age + Life Stage + Life Stage:Temperature + Life Stage:pH + Temperature:pH + Life Stage:DO (ppm) + pH:DO (ppm) + Age:Life Stage:pH + Age:pH:DO (ppm) + Age:Life Stage:Temperature:DO (ppm) + Life Stage:Temperature:pH:DO (ppm)	62.4
	Age + Life Stage + Life Stage:Temperature + Life Stage:pH + Temperature:pH + Life Stage:DO (ppm) + Age:Life Stage:pH + Age:pH:DO (ppm) + Age:Life Stage:Temperature:DO (ppm) + Age:Life Stage:Temperature:pH:DO (ppm)	63.2

Table S4. The ten Bayesian Dirichlet-multinomial regression models from the backwards variable selection process with the lowest widely applicable information criterion (WAIC) values for fungal communities on salamander skin. Ten models are displayed for each predictor set. DO is dissolved oxygen.

Predictor Set	Predictors	ΔWAIC
Spatiotemporal	Life Stage + Life Stage:Site + Age:Week^2 + Site:Week^2	0
	Life Stage + Life Stage:Site + Site:Week^2	6.1
	Life Stage + Life Stage:Site + Age:Week^2 + Site:Week^2 + Age:Life Stage:Site	61.9
	Life Stage + Life Stage:Site + Site:Week^2 + Age:Life Stage:Site	103.6
	Life Stage + Life Stage:Site + Age:Week^2 + Age:Life Stage:Site	103.7
	Life Stage + Life Stage:Site + Age:Week^2 + Site:Week^2 + Life Stage:Site:Week	118.0
	Life Stage + Life Stage:Site + Age:Week^2 + Site:Week^2 + Age:Life Stage:Site + Life Stage:Site:Week	149.3
	Life Stage + Life Stage:Site + Age:Week + Age:Week^2 + Site:Week^2 + Age:Life Stage:Site	153.5
	Life Stage + Life Stage:Site + Site:Week^2 + Age:Life Stage:Site + Life Stage:Site:Week	176.1
	Life Stage + Life Stage:Site + Age:Week + Age:Week^2 + Site:Week^2 + Age:Life Stage:Site + Life Stage:Site:Week	194.8
Water Quality	Age + Life Stage + Life Stage:Temperature + Life Stage:DO (ppm) + Age:Life Stage:pH + Age:Life Stage:pH:DO (ppm) + Age:Temperature:pH:DO (ppm)	0
	Age + Life Stage + Life Stage:Temperature + Life Stage:DO (ppm) + Age:Life Stage:pH + Age:Life Stage:pH:DO (ppm)	30.4
	Age + Life Stage + Life Stage:Temperature + Life Stage:DO (ppm) + Temp:DO (ppm) + Age:Life Stage:pH + Age:Life Stage:pH:DO (ppm) + Age:Temperature:pH:DO (ppm)	47.4
	Age + Life Stage:Temperature + Life Stage:DO (ppm) + Temp:DO (ppm) + Age:Life Stage:pH + Age:Life Stage:pH:DO (ppm) + Age:Temperature:pH:DO (ppm)	66.7
	Age + Life Stage + Life Stage:Temperature + Life Stage:DO (ppm) + Temp:DO (ppm) + Age:Life Stage:pH + Age:Life Stage:pH:DO (ppm)	90.2
	Age + Life Stage + Life Stage:Temperature + Life Stage:DO (ppm) + Temp:DO (ppm) + Age:Life Stage:pH + Temperature:pH:DO (ppm) + Age:Temperature:pH:DO (ppm)	92.0
	Age + Life Stage + Life Stage:Temperature + Life Stage:DO (ppm) + Temp:DO (ppm) + Age:Life Stage:pH + Temperature:pH:DO (ppm) + Age:Life Stage:pH:DO (ppm)	93.8
	Age + Life Stage + Life Stage:Temperature + Life Stage:DO (ppm) + Temp:DO (ppm) + Age:Life Stage:pH + Age:Temperature:pH:DO (ppm)	95.1
	Age + Life Stage + Life Stage:Temperature + Life Stage:DO (ppm) + Age:Life Stage:pH + Temp:pH:DO (ppm) + Age:Life Stage:pH:DO (ppm) + Age:Temperature:pH:DO (ppm)	97.8
	Age + Life Stage + Life Stage:Temperature + Life Stage:DO (ppm) + Temperature:DO (ppm) + Age:Life Stage:pH + Temperature:pH:DO (ppm) + Age:Life Stage:pH:DO (ppm) + Age:Temperature:pH:DO (ppm)	130.9

Table S5. The ten Bayesian Dirichlet-multinomial regression models from the backwards variable selection process with the lowest widely applicable information criterion (WAIC) values for *Bd*-inhibition categories on salamander skin.

Predictors	ΔWAIC
Stratum + Life Stage + Week + Week^2 + Age:Life Stage + Age:Site + Life Stage:Site + Age:Week + Age:Week^2 + Life Stage:Week + Life Stage:Week^2 + Site:Week + Site:Week^2 + Age:Life Stage:Site + Age:Life Stage:Week + Age:Life Stage:Week^2 + Age:Site:Week + Age:Site:Week^2 + Life Stage:Site:Week + Life Stage:Site:Week^2 + Age:Life Stage:Site:Week + Age:Life Stage:Site:Week^2	0
Stratum + Life Stage + Week + Week^2 + Age:Life Stage + Age:Site + Life Stage:Site + Age:Week^2 + Life Stage:Week + Life Stage:Week^2 + Site:Week + Site:Week^2 + Age:Life Stage:Site + Age:Life Stage:Week + Age:Life Stage:Week^2 + Age:Site:Week + Age:Site:Week^2 + Life Stage:Site:Week + Life Stage:Site:Week^2 + Age:Life Stage:Site:Week + Age:Life Stage:Site:Week^2	0.3
Stratum + Life Stage + Week^2 + Age:Life Stage + Age:Site + Life Stage:Site + Age:Week + Age:Week^2 + Life Stage:Week + Life Stage:Week^2 + Site:Week + Site:Week^2 + Age:Life Stage:Site + Age:Life Stage:Week + Age:Life Stage:Week^2 + Age:Site:Week + Age:Site:Week^2 + Life Stage:Site:Week + Life Stage:Site:Week^2 + Age:Life Stage:Site:Week + Age:Life Stage:Site:Week^2	0.8
Stratum + Life Stage + Week + Week^2 + Age:Life Stage + Age:Site + Life Stage:Site + Age:Week + Age:Week^2 + Life Stage:Week + Life Stage:Week^2 + Site:Week + Site:Week^2 + Age:Life Stage:Site + Age:Life Stage:Week + Age:Life Stage:Week^2 + Age:Site:Week^2 + Life Stage:Site:Week + Life Stage:Site:Week^2 + Age:Life Stage:Site:Week + Age:Life Stage:Site:Week^2	0.9
Stratum + Life Stage + Week + Week^2 + Age:Life Stage + Age:Site + Life Stage:Site + Age:Week + Age:Week^2 + Life Stage:Week + Life Stage:Week^2 + Site:Week + Site:Week^2 + Age:Life Stage:Week + Age:Life Stage:Week^2 + Age:Site:Week + Age:Site:Week^2 + Life Stage:Site:Week + Life Stage:Site:Week^2 + Age:Life Stage:Site:Week + Age:Life Stage:Site:Week^2	0.9
Stratum + Life Stage + Week + Week^2 + Age:Life Stage + Age:Site + Life Stage:Site + Age:Week + Age:Week^2 + Life Stage:Week + Life Stage:Week^2 + Site:Week + Site:Week^2 + Age:Life Stage:Site + Age:Life Stage:Week + Age:Life Stage:Week^2 + Age:Site:Week + Age:Site:Week^2 + Life Stage:Site:Week + Age:Life Stage:Site:Week + Age:Life Stage:Site:Week^2	1.5
Stratum + Life Stage + Week + Week^2 + Age:Life Stage + Age:Site + Life Stage:Site + Age:Week + Age:Week^2 + Life Stage:Week + Life Stage:Week^2 + Site:Week + Age:Life Stage:Site + Age:Life Stage:Week + Age:Life Stage:Week^2 + Age:Site:Week + Age:Site:Week^2 + Life Stage:Site:Week + Life Stage:Site:Week^2 + Age:Life Stage:Site:Week + Age:Life Stage:Site:Week^2	1.6
Stratum + Life Stage + Week + Week^2 + Age:Life Stage + Age:Site + Life Stage:Site + Age:Week + Age:Week^2 + Life Stage:Week + Life Stage:Week^2 + Site:Week + Site:Week^2 + Age:Life Stage:Site + Age:Life Stage:Week^2 + Age:Site:Week + Age:Site:Week^2 + Life Stage:Site:Week + Life Stage:Site:Week^2 + Age:Life Stage:Site:Week + Age:Life Stage:Site:Week^2	1.7
Stratum + Life Stage + Week + Week^2 + Age:Life Stage + Age:Site + Life Stage:Site + Age:Week + Age:Week^2 + Life Stage:Week^2 + Site:Week + Site:Week^2 + Age:Life Stage:Site + Age:Life Stage:Week + Age:Life Stage:Week^2 + Age:Site:Week + Age:Site:Week^2 + Life Stage:Site:Week + Life Stage:Site:Week^2 + Age:Life Stage:Site:Week + Age:Life Stage:Site:Week^2	1.9
Stratum + Life Stage + Week + Week^2 + Age:Life Stage + Age:Site + Life Stage:Site + Age:Week + Age:Week^2 + Life Stage:Week + Life Stage:Week^2 + Site:Week + Site:Week^2 + Age:Life Stage:Site + Age:Life Stage:Week + Age:Life Stage:Week^2 + Age:Site:Week + Age:Site:Week^2 + Life Stage:Site:Week + Life Stage:Site:Week^2 + Age:Life Stage:Site:Week^2	2.2

Table S6. The ten Bayesian Dirichlet-multinomial regression models from the backwards variable selection process with the lowest widely applicable information criterion (WAIC) values for lake water samples. Ten models are displayed for each microbial community type.

Microbial Community	Predictors	ΔWAIC
Bacterial	Stratum + Site + Week + Site:Week + Site:Week^2	0
	Site + Week + Site:Week + Site:Week^2	52.6
	Stratum + Site + Week + Week^2 + Site:Week + Site:Week^2	56.4
	Stratum + Site + Week + Week^2 + Site:Week	85.4
	Stratum + Week + Site:Week + Site:Week^2	100.0
	Site + Week + Week^2 + Site:Week + Site:Week^2	115.0
	Stratum + Site + Week + Site:Week^2	147.1
	Stratum + Week + Week^2 + Site:Week + Site:Week^2	166.9
	Stratum + Site + Site:Week + Site:Week^2	186.8
	Stratum + Site + Week^2 + Site:Week + Site:Week^2	248.3
Fungal	Stratum + Week + Week^2 + Site:Week + Site:Week^2	0
	Stratum + Site + Week + Week^2 + Site:Week + Site:Week^2	32.4
	Stratum + Site + Week + Site:Week + Site:Week^2	44.1
	Stratum + Site + Week + Week^2 + Site:Week	63.5
	Stratum + Week + Site:Week + Site:Week^2	64.7
	Stratum + Week + Week^2 + Site:Week	91.4
	Stratum + Week + Week^2 + Site:Week^2	116.9
	Stratum + Site + Week + Week^2 + Site:Week^2	121.9
	Stratum + Week^2 + Site:Week + Site:Week^2	222.3
	Stratum + Site + Week^2 + Site:Week + Site:Week^2	247.9
Bd-Inhibition Categories	Site + Week + Site:Week + Site:Week^2	0
	Site + Week + Week^2 + Site:Week + Site:Week^2	1.4
	Stratum + Site + Week + Site:Week + Site:Week^2	1.9
	Site + Site:Week + Site:Week^2	2.3
	Stratum + Site + Week + Week^2 + Site:Week + Site:Week^2	2.9
	Site + Week + Week^2 + Site:Week	4.3
	Site + Week^2 + Site:Week + Site:Week^2	4.4
	Stratum + Site + Week + Week^2 + Site:Week	5.6
	Site + Week + Week^2 + Site:Week^2	5.6
	Week + Site:Week + Site:Week^2	6.1

Table S7. The ten Bayesian Dirichlet-multinomial regression models from the backwards variable selection process with the lowest widely applicable information criterion (WAIC) values for lake substrate samples. Ten models are displayed for each microbial community type.

Microbial Community	Predictors	ΔWAIC
Bacterial	Stratum + Site + Week + Site:Week + Site:Week^2	0
	Stratum + Week + Site:Week + Site:Week^2	1.9
	Stratum + Site + Week + Site:Week	76.7
	Stratum + Week + Week^2 + Site:Week + Site:Week^2	220.0
	Stratum + Site + Week + Week^2 + Site:Week + Site:Week^2	238.4
	Stratum + Site + Week + Week^2 + Site:Week	242.1
	Stratum + Site + Week + Site:Week^2	268.4
	Stratum + Site + Site:Week + Site:Week^2	353.9
	Stratum + Site + Week + Week^2 + Site:Week^2	483.9
	Stratum + Site + Week^2 + Site:Week + Site:Week^2	544.3
Fungal	Stratum + Week + Site:Week + Site:Week^2	0
	Stratum + Site + Week + Site:Week + Site:Week^2	13.6
	Stratum + Week + Site:Week	90.2
	Stratum + Site + Week + Site:Week	107.2
	Stratum + Week + Week^2 + Site:Week + Site:Week^2	242.9
	Stratum + Site + Week + Week^2 + Site:Week + Site:Week^2	279.4
	Stratum + Site + Week + Week^2 + Site:Week	305.0
	Stratum + Site:Week + Site:Week^2	331.9
	Stratum + Site + Site:Week + Site:Week^2	368.4
	Stratum + Week + Site:Week^2	386.4
Bd-Inhibition Categories	Stratum + Site + Week + Site:Week + Site:Week^2	0
	Stratum + Week + Site:Week + Site:Week^2	0.9
	Stratum + Site + Week + Site:Week	5.0
	Stratum + Site + Week + Week^2 + Site:Week	7.6
	Stratum + Week + Week^2 + Site:Week + Site:Week^2	7.7
	Stratum + Site + Week + Week^2 + Site:Week + Site:Week^2	8.9
	Stratum + Site + Week + Site:Week^2	29.8
	Stratum + Site + Site:Week + Site:Week^2	30.6
	Stratum + Site + Week^2 + Site:Week + Site:Week^2	32.4
	Stratum + Site + Week + Week^2 + Site:Week^2	32.9

16S proportional abundance plots

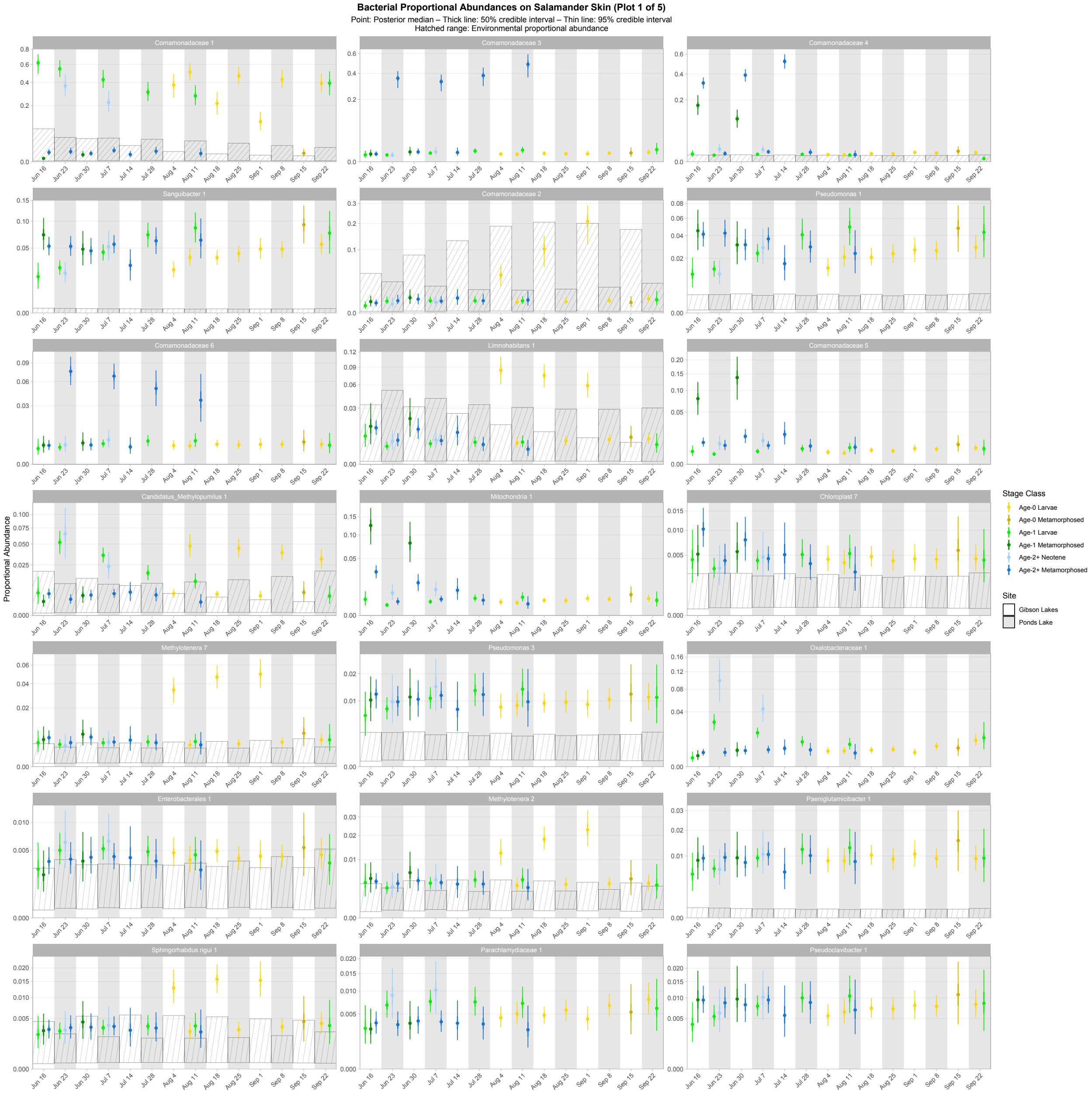

Figure S9. First plot of proportional abundance predictions from Bayesian Dirichlet-multinomial regression models for the top 100 bacterial taxa. Points, thick lines, and thin lines represent posterior medians, 50% credible intervals, and 95% credible intervals, respectively. Hatched ranges represent environmental proportional abundances. Taxa without hatched ranges were not detected in either water or substrate. Note the square root scale on the y-axis. Taxa are ordered (left to right, top to bottom) by descending average proportion of reads in salamander samples in which each combination of site and life stage receives equal weight.

Bacterial Proportional Abundances on Salamander Skin (Plot 2 of 5)

Point: Posterior median – Thick line: 50% credible interval – Thin line: 95% credible interval
Hatched range: Environmental proportional abundance

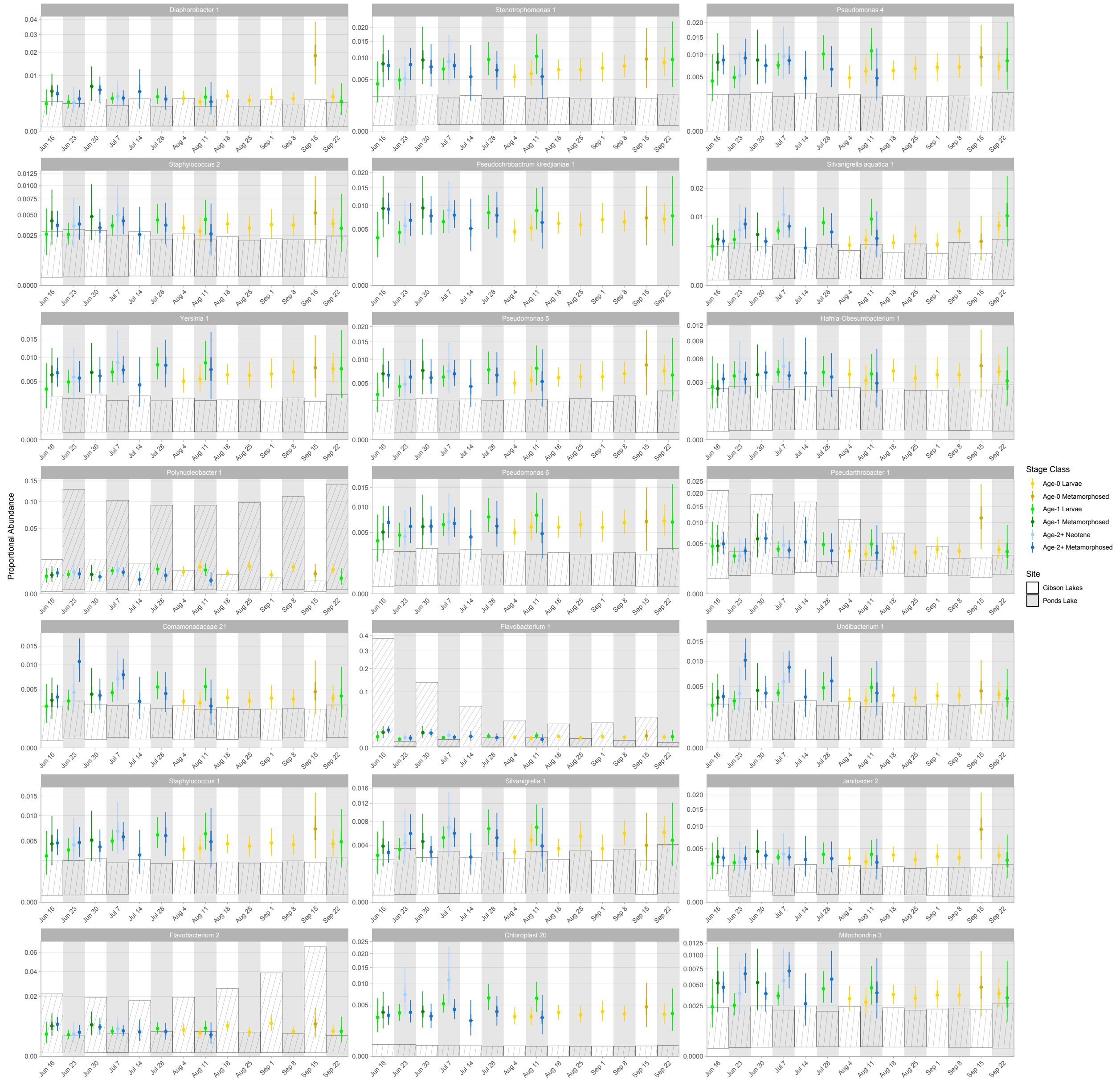

Figure S10. Second plot of proportional abundance predictions from Bayesian Dirichlet-multinomial regression models for the top 100 bacterial taxa. Points, thick lines, and thin lines represent posterior medians, 50% credible intervals, and 95% credible intervals, respectively. Hatched ranges represent environmental proportional abundances. Taxa without hatched ranges were not detected in either water or substrate. Note the square root scale on the y-axis. Taxa are ordered (left to right, top to bottom) by descending average proportion of reads in salamander samples in which each combination of site and life stage receives equal weight.

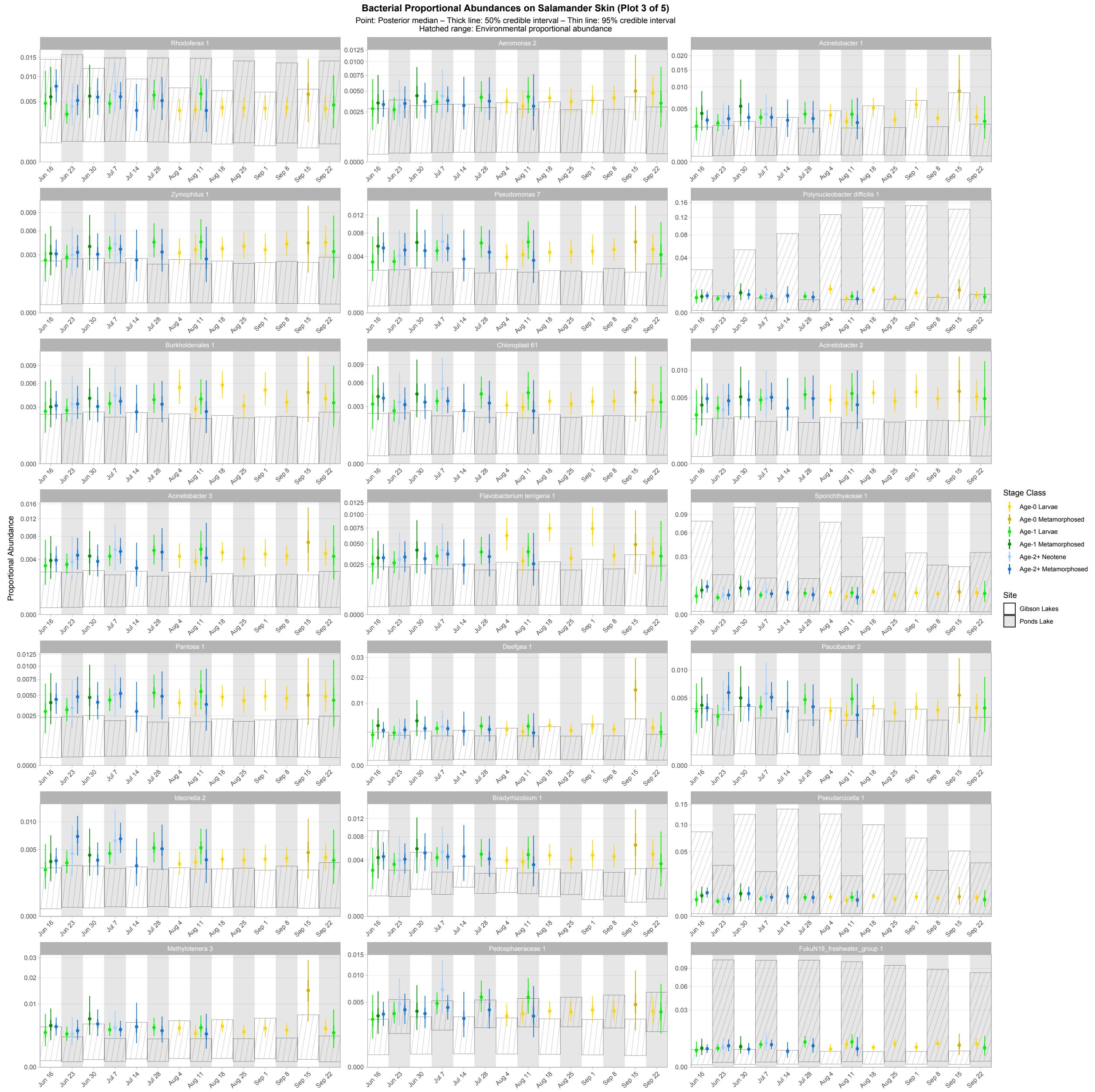

Figure S11. Third plot of proportional abundance predictions from Bayesian Dirichlet-multinomial regression models for the top 100 bacterial taxa. Points, thick lines, and thin lines represent posterior medians, 50% credible intervals, and 95% credible intervals, respectively. Hatched ranges represent environmental proportional abundances. Taxa without hatched ranges were not detected in either water or substrate. Note the square root scale on the y-axis. Taxa are ordered (left to right, top to bottom) by descending average proportion of reads in salamander samples in which each combination of site and life stage receives equal weight.

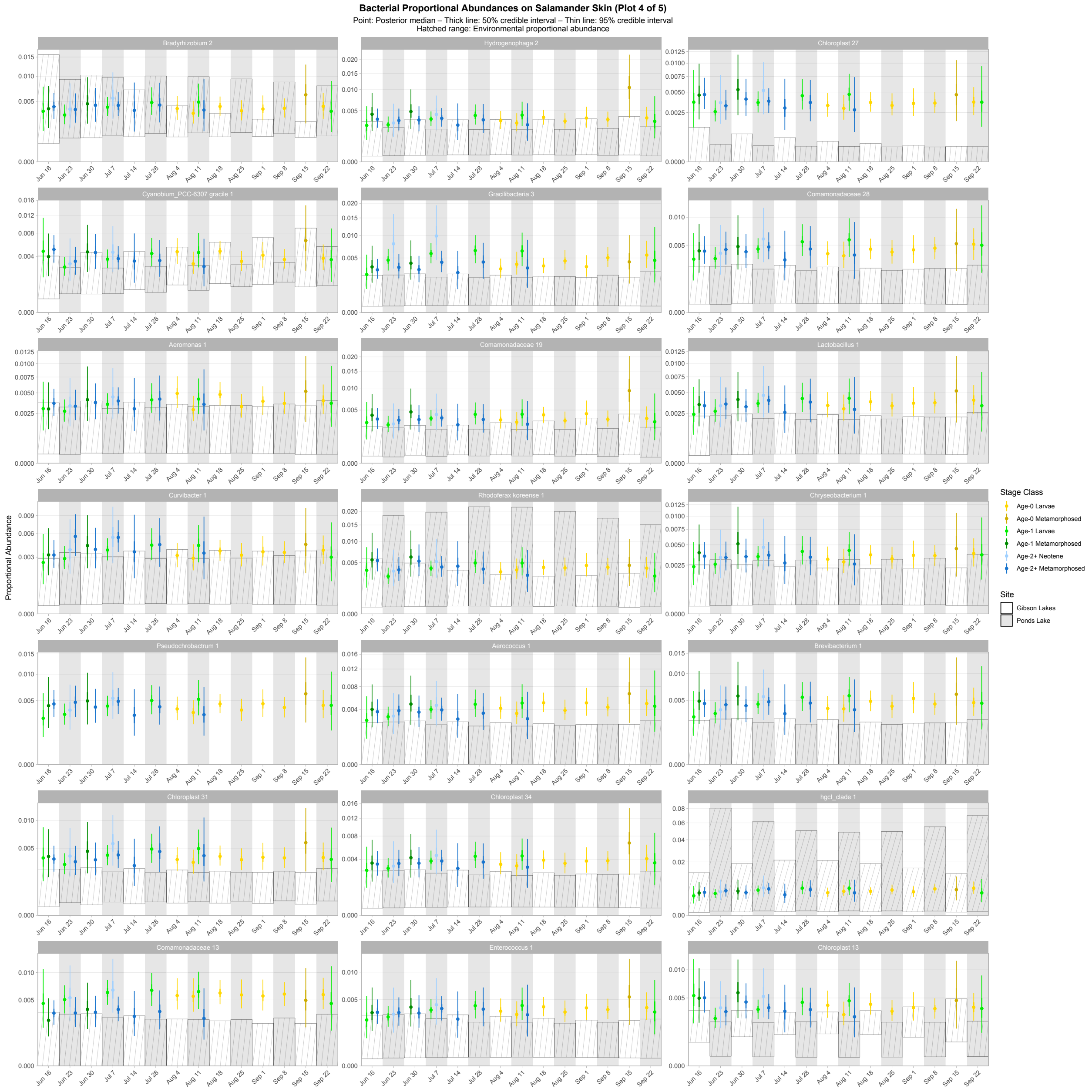

Figure S12. Fourth plot of proportional abundance predictions from Bayesian Dirichlet-multinomial regression models for the top 100 bacterial taxa. Points, thick lines, and thin lines represent posterior medians, 50% credible intervals, and 95% credible intervals, respectively. Hatched ranges represent environmental proportional abundances. Taxa without hatched ranges were not detected in either water or substrate. Note the square root scale on the y-axis. Taxa are ordered (left to right, top to bottom) by descending average proportion of reads in salamander samples in which each combination of site and life stage receives equal weight.

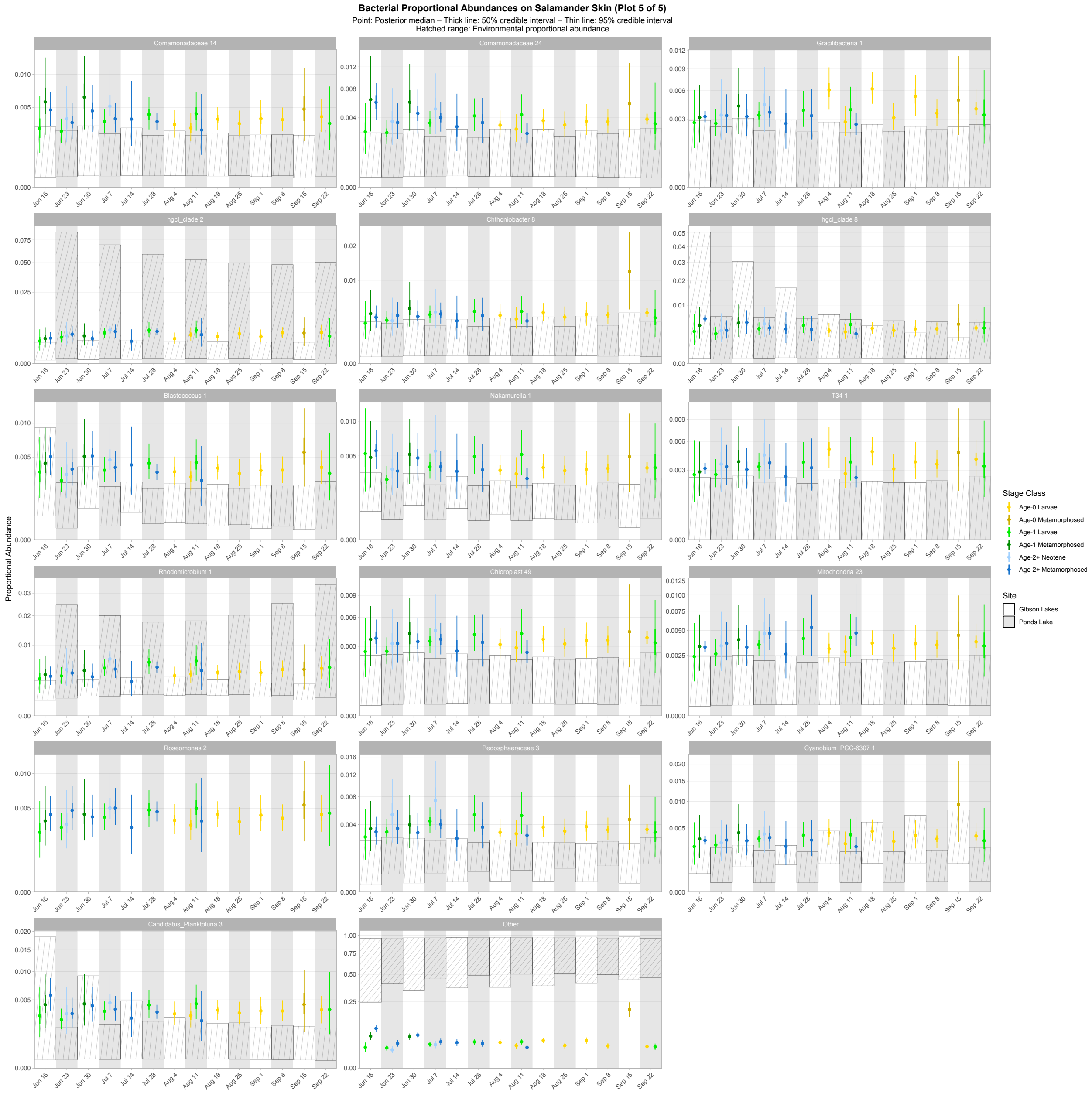

Figure S13. Fifth plot of proportional abundance predictions from Bayesian Dirichlet-multinomial regression models for the top 100 bacterial taxa. Points, thick lines, and thin lines represent posterior medians, 50% credible intervals, and 95% credible intervals, respectively. Hatched ranges represent environmental proportional abundances. Taxa without hatched ranges were not detected in either water or substrate. Note the square root scale on the y-axis. Taxa are ordered (left to right, top to bottom) by descending average proportion of reads in salamander samples in which each combination of site and life stage receives equal weight.

ITS proportional abundance plots

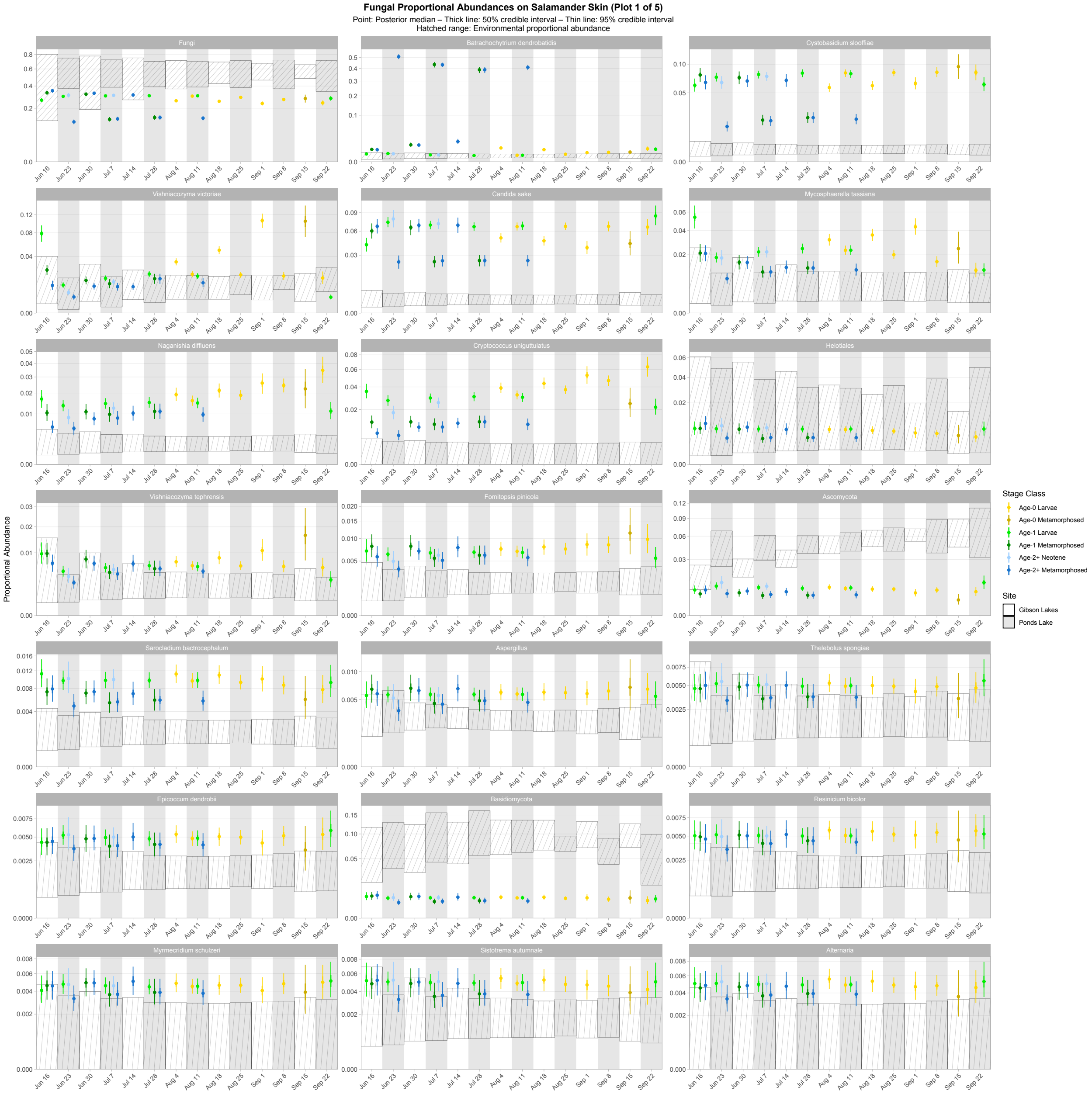

Figure S14. First plot of proportional abundance predictions from Bayesian Dirichlet-multinomial regression models for the top 100 fungal taxa. Points, thick lines, and thin lines represent posterior medians, 50% credible intervals, and 95% credible intervals, respectively. Hatched ranges represent environmental proportional abundances. Taxa without hatched ranges were not detected in either water or substrate. Note the square root scale on the y-axis. Taxa are ordered (left to right, top to bottom) by descending average proportion of reads in salamander samples in which each combination of site and life stage receives equal weight.

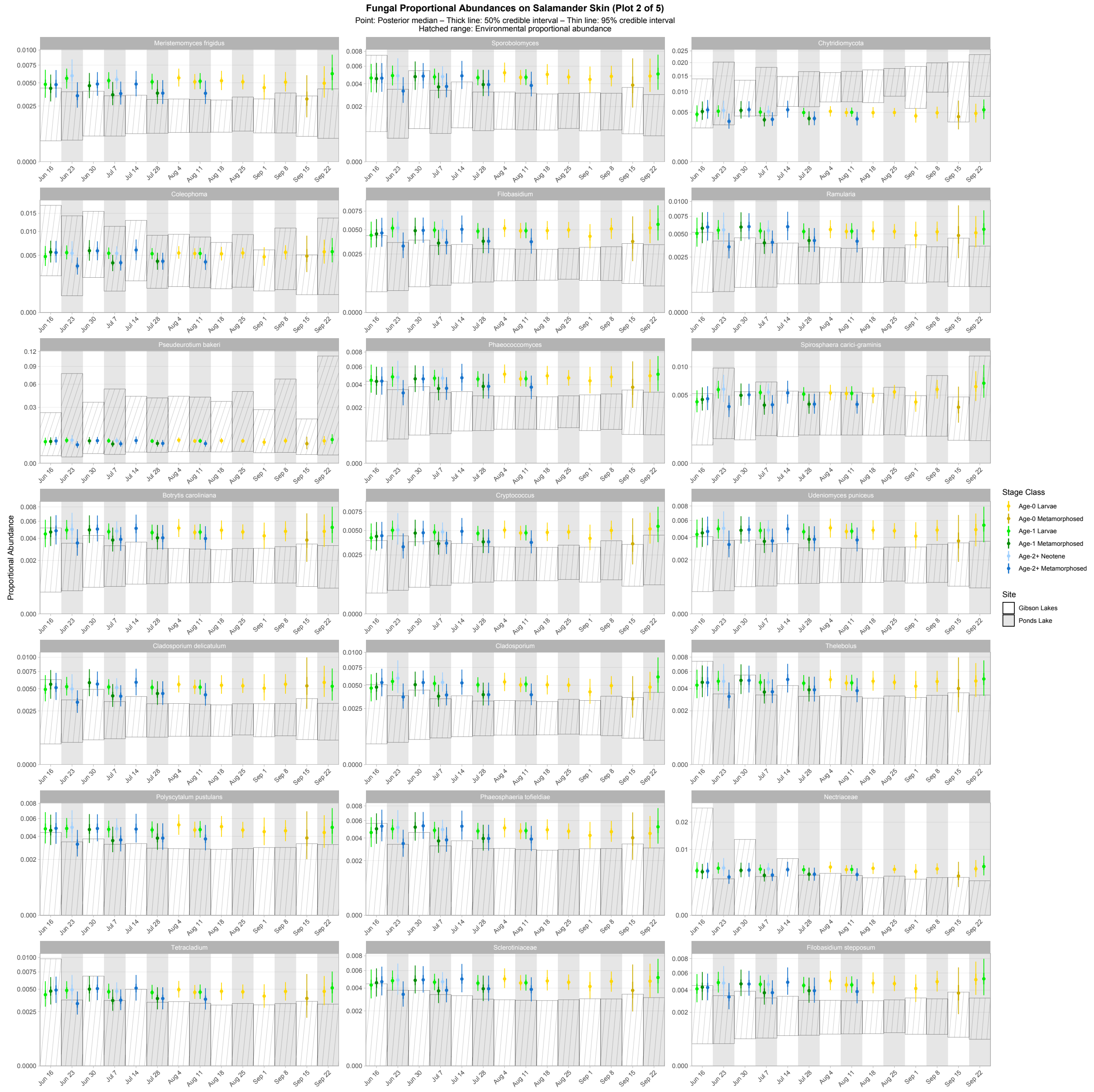

Figure S15. Second plot of proportional abundance predictions from Bayesian Dirichlet-multinomial regression models for the top 100 fungal taxa. Points, thick lines, and thin lines represent posterior medians, 50% credible intervals, and 95% credible intervals, respectively. Hatched ranges represent environmental proportional abundances. Taxa without hatched ranges were not detected in either water or substrate. Note the square root scale on the y-axis. Taxa are ordered (left to right, top to bottom) by descending average proportion of reads in salamander samples in which each combination of site and life stage receives equal weight.

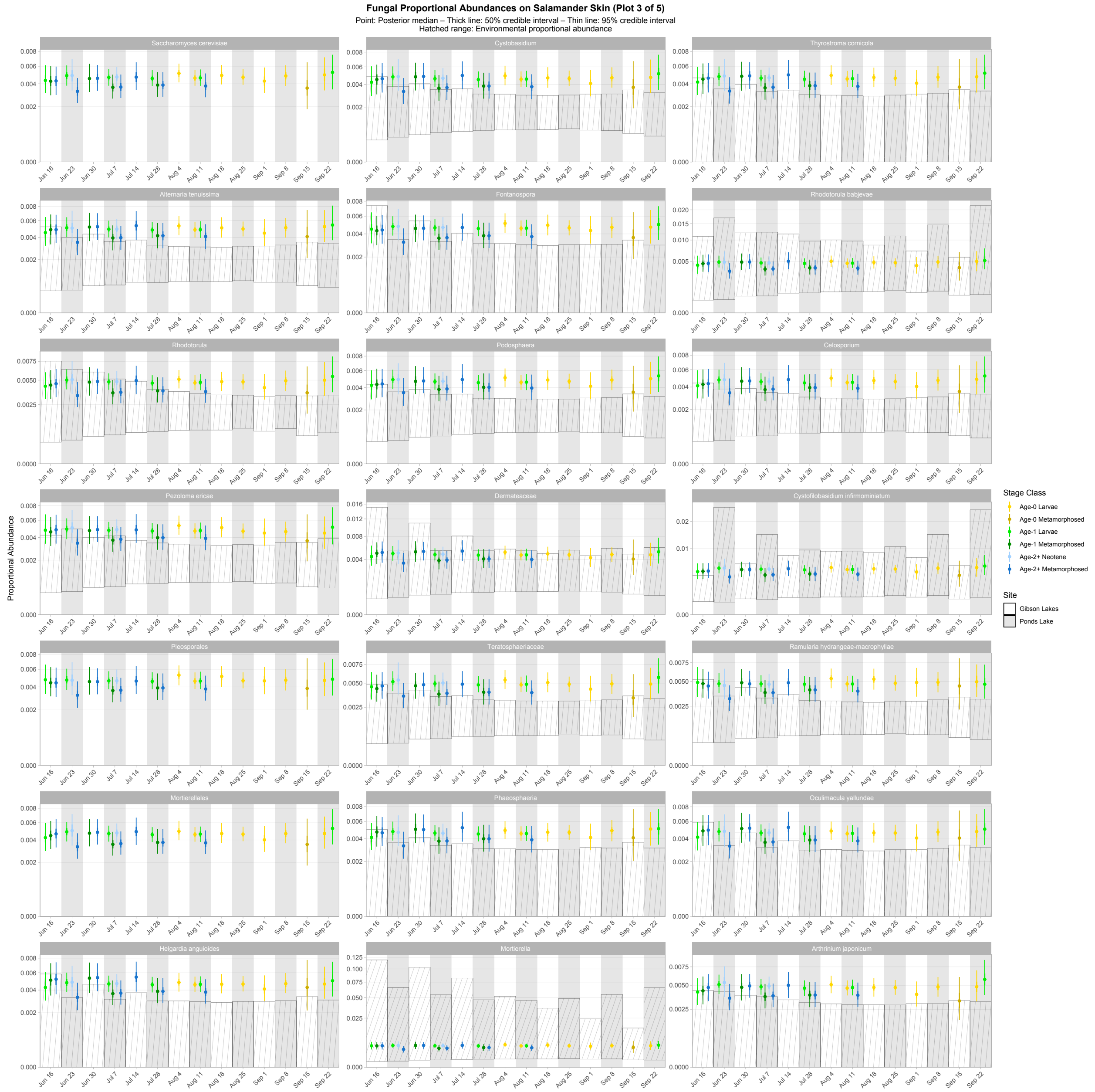

Figure S16. Third plot of proportional abundance predictions from Bayesian Dirichlet-multinomial regression models for the top 100 fungal taxa. Points, thick lines, and thin lines represent posterior medians, 50% credible intervals, and 95% credible intervals, respectively. Hatched ranges represent environmental proportional abundances. Taxa without hatched ranges were not detected in either water or substrate. Note the square root scale on the y-axis. Taxa are ordered (left to right, top to bottom) by descending average proportion of reads in salamander samples in which each combination of site and life stage receives equal weight.

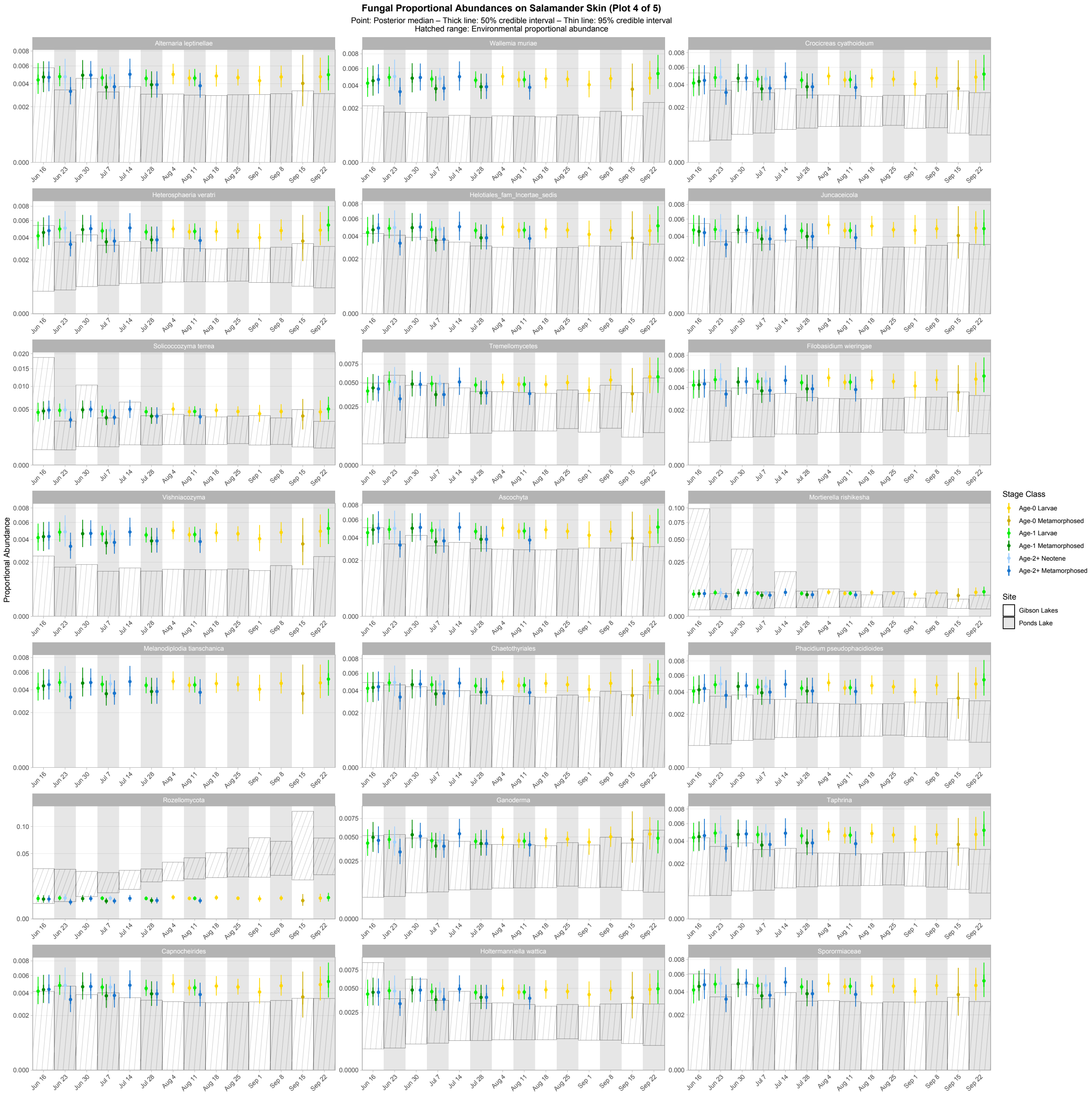

Figure S17. Fourth plot of proportional abundance predictions from Bayesian Dirichlet-multinomial regression models for the top 100 fungal taxa. Points, thick lines, and thin lines represent posterior medians, 50% credible intervals, and 95% credible intervals, respectively. Hatched ranges represent environmental proportional abundances. Taxa without hatched ranges were not detected in either water or substrate. Note the square root scale on the y-axis. Taxa are ordered (left to right, top to bottom) by descending average proportion of reads in salamander samples in which each combination of site and life stage receives equal weight.

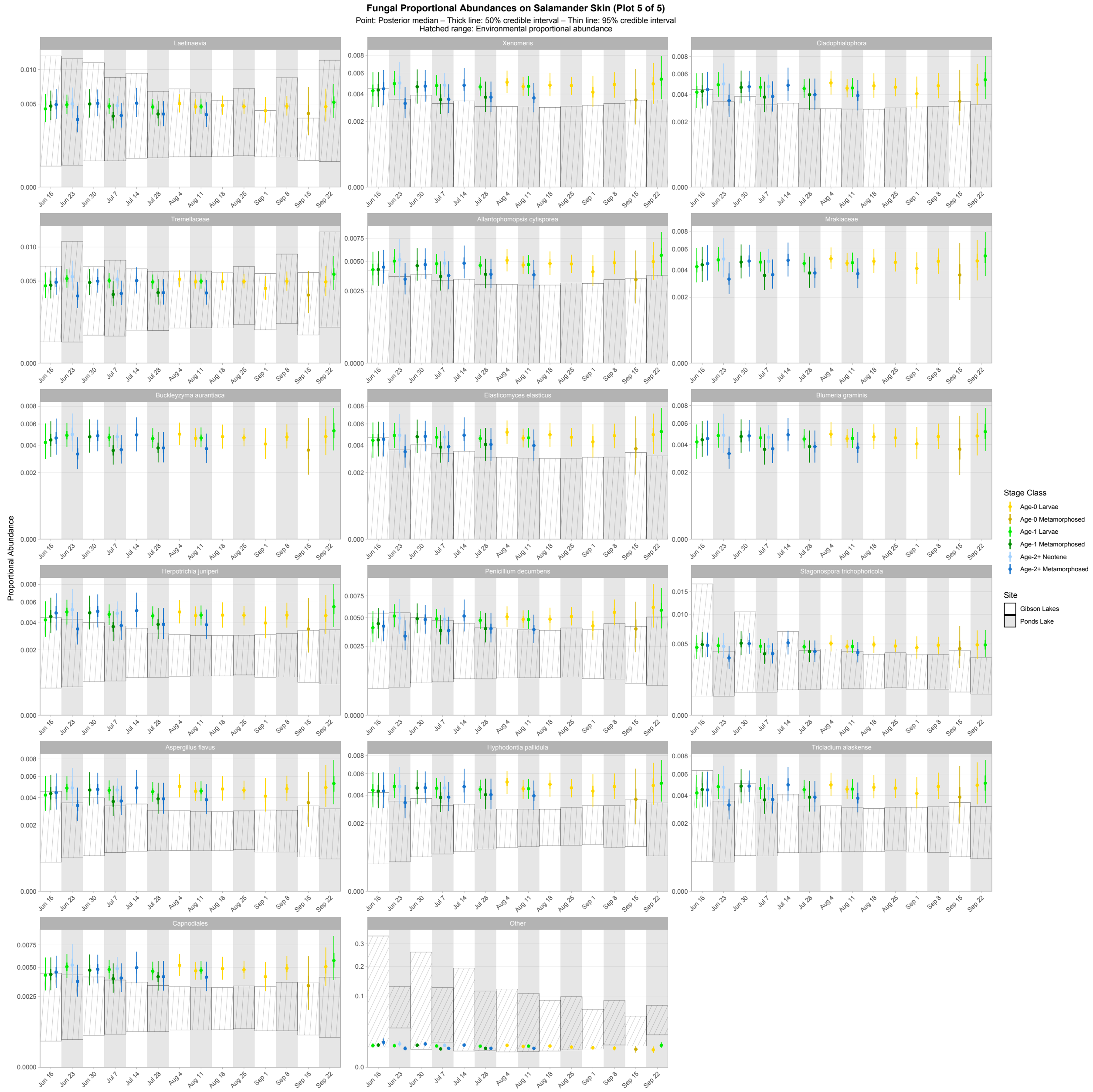

Figure S18. Fifth plot of proportional abundance predictions from Bayesian Dirichlet-multinomial regression models for the top 100 fungal taxa. Points, thick lines, and thin lines represent posterior medians, 50% credible intervals, and 95% credible intervals, respectively. Hatched ranges represent environmental proportional abundances. Taxa without hatched ranges were not detected in either water or substrate. Note the square root scale on the y-axis. Taxa are ordered (left to right, top to bottom) by descending average proportion of reads in salamander samples in which each combination of site and life stage receives equal weight.

Model code

Stan code for Bayesian Dirichlet-multinomial regression model

```
//// Dirichlet-Multinomial Regression Model.

data{

  //// Define variables.
  int<lower=1> NSamples; // Number of samples.
  int<lower=1> NTaxa; // Number of taxa.
  int<lower=1> NStrata; // Number of strata.
  matrix[NSamples,NStrata] StratumMatrix; // Predictor matrix for strata.
  int<lower=1> NPredictors; // Number of non-strata predictors.
  matrix[NSamples,NPredictors] PredictorMatrix; // Non-strata predictor matrix.
  int ReadsMatrix[NSamples,NTaxa]; // Reads response matrix.
  real<lower=0> sd_prior; // Regression coefficient and precision parameter standard deviation
        prior.

}

parameters{

  //// Specify parameters.
  simplex[NTaxa] p[NSamples]; // Each sample's set of modeled proportions.
  vector[NTaxa-1] beta_0_raw; // Intercept raw regression coefficient vector.
  matrix[NTaxa-1,NStrata-1] beta_stratum_raw; // Stratum raw regression coefficient matrix.
  vector<lower=0>[NTaxa-1] sigma2_stratum; // Stratum common variance vector.
  matrix[NTaxa-1,NPredictors] beta_pred_raw; // Non-stratum raw regression coefficient matrix.
  real theta; // Precision parameter.

}

transformed parameters{

  //// Specify transformed parameters.
  vector[NTaxa] beta_0; // Intercept regression coefficient vector.
  matrix[NTaxa,NStrata] beta_stratum; // Stratum regression coefficient matrix.
  vector<lower=0>[NTaxa-1] sigma_stratum; // Stratum common standard deviation vector.
  matrix[NTaxa,NPredictors] beta_pred; // Non-stratum regression coefficient matrix.
  real exptheta; // Exponentiated precision parameter.

  //// Define transformed parameters.

  // Intercept regression coefficients.
```

```
  beta_0[NTaxa]=0; // Set last taxon's intercept to zero.
  for(k in 1:(NTaxa-1)){
    beta_0[k]=beta_0_raw[k]; // Set intercepts of other taxa to the raw betas.
  }

  // Stratum regression coefficients.
  for(j in 1:NStrata){
    beta_stratum[NTaxa,j]=0; // Set last taxon's stratum regression coefficients to zero.
  }
  for(k in 1:(NTaxa-1)){
    for(j in 1:(NStrata-1)){
      beta_stratum[k,j]=beta_stratum_raw[k,j]; // Set stratum regression coefficients of other taxa
          to the raw betas (execpt for the last stratum).
    }
    beta_stratum[k,NStrata]=-sum(beta_stratum_raw[k,1:(NStrata-1)]); // Apply a sum to zero
          constraint for the last stratum.
    sigma_stratum[k]=sqrt(sigma2_stratum[k]); // Calculate stratum common standard deviation as
          the square root of the common variance.
  }

  // Non-stratum regression coefficients.
  for(j in 1:NPredictors){
    beta_pred[NTaxa,j]=0; // Set last taxon's regression coefficients to zero.
    for(k in 1:(NTaxa-1)){
      beta_pred[k,j]=beta_pred_raw[k,j]; // Set regression coefficients of other taxa to the raw
          betas.
    }
  }

  // Create exponentiated precision parameter.
  exptheta=exp(theta);

}

model{

  //// Provide priors.

  // Intercept regression coefficients.
  for(k in 1:(NTaxa-1)){
    beta_0_raw[k]~normal(0,sd_prior);
  }

  // Stratum regression coefficients.
  for(k in 1:(NTaxa-1)){
```

```
    for(j in 1:(NStrata-1)){
      beta_stratum_raw[k,j]~normal(0,sigma_stratum[k]);
    }
  }

  // Stratum common variance.
  for(k in 1:(NTaxa-1)){
    sigma2_stratum[k]~inv_gamma(0.01,0.01);
  }

  // Non-stratum regression coefficients.
  for(j in 1:NPredictors){
    for(k in 1:(NTaxa-1)){
      beta_pred_raw[k,j]~normal(0,sd_prior);
    }
  }

  // Precision parameter.
  theta~normal(0,sd_prior);

  //// State likelihood.

  // Loop through each sample.
  for(i in 1:NSamples){

    // Define local variable.
    vector[NTaxa] eta;

    // Loop through each taxon.
    for(j in 1:NTaxa){
      // Linear model for eta.

        eta[j]=beta_0[j]+beta_stratum[j,1:NStrata]*transpose(StratumMatrix[i,1:NStrata])+beta_p
        red[j,1:NPredictors]*transpose(PredictorMatrix[i,1:NPredictors]);
    }

    // Relate sample eta's to probabilities with the Dirichlet distribution.
    p[i]~dirichlet(softmax(eta[1:NTaxa])*exptheta);

    // Relate probabilities to counts with the multinomial distribution.
    ReadsMatrix[i,1:NTaxa]~multinomial(p[i]);

  }

}
```

JAGS code for Bayesian beta-binomial regression model

```
model{

  ##################
  ### Likelihood ###
  ##################

  # Loop through each sample.
  for(i in 1:NSamples){

    # Relate p_Inhib and total 16S read count to inhibitory read count with the binomial
          distribution.
    InhibReadCount[i]~dbinom(p_Inhib[i],TotalBioReads16S[i])

    # Account for overdispersion on the predictor side with the beta distribution.
    p_Inhib[i]~dbeta(alpha_Inhib[i],beta_Inhib[i])

    # Define alpha and beta parameters of the beta distribution on the predictor side.
    # PropInhib is the estimated proportion of inhibitory reads.
    # phi is an overdispersion parameter.
    alpha_Inhib[i]<-PropInhib[i]*phi
    beta_Inhib[i]<-(1-PropInhib[i])*phi

    # Linear model for proportion of Bd with a logit link. Predictor is on the logit scale.
    logit(PropBd[i])<-beta0+betaInhib*logit(PropInhib[i])

    # Define alpha and beta parameters of the beta distribution on the response side.
    # PropBd is the estimated proportion of Bd reads.
    # phi is an overdispersion parameter.
    alpha_Bd[i]<-PropBd[i]*phi
    beta_Bd[i]<-(1-PropBd[i])*phi

    # Account for overdispersion on the response side with the beta distribution.
    p_Bd[i]~dbeta(alpha_Bd[i],beta_Bd[i])

    # Relate p_Bd and total ITS read count to Bd read count with the binomial distribution.
    BdReadCount[i]~dbinom(p_Bd[i],TotalBioReadsITS[i])

  }

  ##############
  ### Priors ###
  ##############
```

```
  # Provide a uniform prior for the proportion of inhibitory reads in each sample.
  for(i in 1:NSamples){
    PropInhib[i]~dbeta(1,1)
  }

  # Intercept term.
  beta0~dnorm(0,1e-6)

  # Regression coefficient for proportion of inhibitory reads.
  betaInhib~dnorm(0,1e-6)

  # Precision parameter.
  phi~dgamma(0.01,0.01)

}
```

References

Bletz, M. C., Perl, R. G., Bobowski, B. T., Japke, L. M., Tebbe, C. C., Dohrmann, A. B., et al. (2017a). Amphibian skin microbiota exhibits temporal variation in community structure but stability of predicted *Bd*-inhibitory function. *The ISME Journal* 11(7), 1521-1534.

Callahan, B. J., McMurdie, P. J., Rosen, M. J., Han, A. W., Johnson, A. J. A., and Holmes, S. P., et al. (2016). DADA2: High-resolution sample inference from Illumina amplicon data. *Nature Methods* 13(7), 581.

Caporaso, J. G., Lauber, C. L., Walters, W. A., Berg-Lyons, D., Lozupone, C. A., Turnbaugh, P. J., et al. (2011). Global patterns of 16S rRNA diversity at a depth of millions of sequences per sample. *Proceedings of the National Academy of Sciences* 108(Supplement 1), 4516-4522. doi: 10.1073/pnas.1000080107

Carpenter, B., Gelman, A., Hoffman, M. D., Lee, D., Goodrich, B., Betancourt, M., et al. (2017). Stan: A probabilistic programming language. *Journal of Statistical Software* 76(1). doi: 10.18637/jss.v076.i01

Gardes, M., and Bruns, T. D. (1993). ITS primers with enhanced specificity for basidiomycetes-Application to the identification of mycorrhizae and rusts. *Molecular Ecology* 2(2), 113-118. doi: 10.1111/j.1365-294X.1993.tb00005.x

Gelman, A (2008). Scaling regression inputs by dividing by two standard deviations. *Statistics in Medicine* 27(15), 2865-2873. doi: 10.1002/sim.3107

Gelman, A., Hwang, J., and Vehtari, A. (2014). Understanding predictive information criteria for Bayesian models. *Statistics and Computing* 24(6), 997-1016.

Harrison, J. G., Calder, J. W., Shastry, V., and Buerkle, A. C. (2020). Dirichlet-multinomial modelling outperforms alternatives for analysis of microbiome and other ecological count data. *Molecular Ecology Resources* 20(2), 481-497. doi: 10.1111/1755-0998.13128

Harrison, J. G., Calder, J. W., Shuman, B., and Buerkle, A. C. (2021). The quest for absolute abundance: the use of internal standards for DNA-based community ecology. *Molecular Ecology Resources* 21(1), 30-43. doi: 10.1111/1755-0998.13247

Hile, R. (1936). Age and growth of the cisco *Leucichthys artedi* (Le Sueur), in the lakes of the northeastern highlands, Wisconsin. *Bulletin of the U.S. Bureau of Fisheries* 48:211-317. doi: 10.1002/iroh.19380360103

Kruger, A. (2020). Frog skin microbiota vary with host species and environment but not chytrid infection. *Frontiers in Microbiology* 1330. doi: 10.3389/fmicb.2020.01330

Kueneman, J. G., Woodhams, D. C., Van Treuren, W., Archer, H. M., Knight, R., and McKenzie, V. J. (2016b). Inhibitory bacteria reduce fungi on early life stages of endangered Colorado boreal toads (*Anaxyrus boreas*). *The ISME Journal* 10(4), 934-944.

Longo, A. V., Savage, A. E., Hewson, I., and Zamudio, K. R. (2015). Seasonal and ontogenetic variation of skin microbial communities and relationships to natural disease dynamics in declining amphibians. *Royal Society Open Science* 2(7), 140377. doi: 10.1098/rsos.140377

Nilsson, R. H., Larsson, K. H., Taylor, A. F. S., Bengtsson-Palme, J., Jeppesen, T. S., Schigel, D., et al. (2019). The UNITE database for molecular identification of fungi: handling dark taxa and parallel taxonomic classifications. *Nucleic Acids Research* 47(D1), D259-D264. doi: 10.1093/nar/gky1022

Ogle, D. H., Wheeler, P., and Dinno, A. (2020). FSA: Fisheries stock analysis. R package version 0.8.30. https://github.com/droglenc/FSA.

Pagès, H., Aboyoun, P., Gentleman, R., and DebRoy, S. (2019). Biostrings: efficient manipulation of biological strings. R package version 2.54.0.

Parada, A. E., Needham, D. M., and Fuhrman, J. A. (2016). Every base matters: Assessing small subunit rRNA primers for marine microbiomes with mock communities, time series and global field samples. *Environmental Microbiology* 18(5), 1403-1414. doi: 10.1111/1462-2920.13023

Paradis, E., and Schliep, K. (2019). ape 5.0: An environment for modern phylogenetics and evolutionary analyses in R. *Bioinformatics* 35:526-528.

Plummer, M. (2003). JAGS: A program for analysis of Bayesian graphical models using Gibbs sampling. In *Proceedings of the 3rd international workshop on distributed statistical computing* (Vol. 124, No. 125.10, pp. 1-10).

Plummer, M. (2019). rjags: Bayesian graphical models using MCMC. R package version 4-10. https://CRAN.R-project.org/package=rjags.

Price, M. N., Dehal, P. S., and Arkin, A. P. (2010). FastTree 2–Approximately maximum-likelihood trees for large alignments. *PLoS One* 5(3), e9490. doi: 10.1371/journal.pone.0009490

Quast, C., Pruesse, E., Yilmaz, P., Gerken, J., Schweer, T., Yarza, P., et al. (2012). The SILVA ribosomal RNA gene database project: improved data processing and web-based tools. *Nucleic acids research* 41(D1), D590-D596. doi: 10.1093/nar/gks1219

Revell, L. J. (2012). phytools: An R package for phylogenetic comparative biology (and other things). *Methods Ecol. Evol.* 3 217-223.

Sennhenn-Reulen, H. (2018). Bayesian regression for a Dirichlet distributed response using Stan. *arXiv* [Preprint]. doi: 10.48550/arXiv.1808.06399

Sievers, F., Wilm, A., Dineen, D., Gibson, T. J., Karplus, K., Li, W., et al. (2011). Fast, scalable generation of high-quality protein multiple sequence alignments using Clustal Omega. *Molecular Systems Biology* 7(1), 539. doi: 10.1038/msb.2011.75

Stan Development Team (2020). RStan: The R interface to Stan. R package version 2.21.2. http://mc-stan.org/.

Wang, Q., Garrity, G. M., Tiedje, J. M., and Cole, J. R. (2007). Naïve Bayesian classifier for rapid assignment of rRNA sequences into the new bacterial taxonomy. *Appl. Environ. Microbiol.* 73(16), 5261-5267. doi: 10.1128/AEM.00062-07

White, T. J., Bruns, T., Lee, S., and Taylor, J. (1990). Amplification and direct sequencing of fungal ribosomal RNA genes for phylogenetics. *PCR Protocols: A Guide to Methods and Applications* 18(1), 315-322.

Wolodzko, T. (2019). extraDistr: Additional univariate and multivariate distributions. R package version 1.8.11. https://CRAN.R-project.org/package=extraDistr.

Woodhams, D. C., Alford, R. A., Antwis, R. E., Archer, H., Becker, M. H., Belden, L. K., et al. (2015). Antifungal isolates database of amphibian skin-associated bacteria and function against emerging fungal pathogens. *Ecology* 96(2), 595-595. doi: 10.1890/14-1837.1

Zhang, Z., Schwartz, S., Wagner, L., and Miller, W. (2000). A greedy algorithm for aligning DNA sequences. *Journal of Computational Biology* 7(1-2), 203-214. doi: 10.1089/10665270050081478
